# Selecting Superior *De Novo* Transcriptome Assemblies: Lessons Learned by Leveraging the Best Plant Genome

**DOI:** 10.1371/journal.pone.0146062

**Published:** 2016-01-05

**Authors:** Loren A. Honaas, Eric K. Wafula, Norman J. Wickett, Joshua P. Der, Yeting Zhang, Patrick P. Edger, Naomi S. Altman, J. Chris Pires, James H. Leebens-Mack, Claude W. dePamphilis

**Affiliations:** 1 Biology Department, Penn State, University Park, Pennsylvania, 16802, United States of America; 2 Division of Biological Sciences, University of Missouri, Columbia, Missouri, 65211, United States of America; 3 Department of Statistics, Penn State, University Park, Pennsylvania, 16802, United States of America; 4 Department of Plant Biology, University of Georgia, Athens, Georgia, 30602, United States of America; Ghent University, BELGIUM

## Abstract

Whereas *de novo* assemblies of RNA-Seq data are being published for a growing number of species across the tree of life, there are currently no broadly accepted methods for evaluating such assemblies. Here we present a detailed comparison of 99 transcriptome assemblies, generated with 6 *de novo* assemblers including CLC, Trinity, SOAP, Oases, ABySS and NextGENe. Controlled analyses of *de novo* assemblies for *Arabidopsis thaliana* and *Oryza sativa* transcriptomes provide new insights into the strengths and limitations of transcriptome assembly strategies. We find that the leading assemblers generate reassuringly accurate assemblies for the majority of transcripts. At the same time, we find a propensity for assemblers to fail to fully assemble highly expressed genes. Surprisingly, the instance of true chimeric assemblies is very low for all assemblers. Normalized libraries are reduced in highly abundant transcripts, but they also lack 1000s of low abundance transcripts. We conclude that the quality of *de novo* transcriptome assemblies is best assessed through consideration of a *combination* of metrics: 1) proportion of reads mapping to an assembly 2) recovery of conserved, widely expressed genes, 3) N_50_ length statistics, and 4) the total number of unigenes. We provide benchmark Illumina transcriptome data and introduce *SCE*RNA, a broadly applicable modular protocol for *de novo* assembly improvement. Finally, our *de novo* assembly of the *Arabidopsis* leaf transcriptome revealed ~20 putative *Arabidopsis* genes lacking in the current annotation.

## Introduction

Massively parallel second generation sequencing technologies (SGS syn. NGS—next generation sequencing) have substantially reduced the cost of generating transcriptome sequence data, driving a rapid expansion of sequencing data resources[1]. The Sequence Read Archive (SRA) at the National Center for Biotechnology Information (NCBI) is growing at an exponential rate and at the beginning of 2015 the total number of bases exceeded 3.8x10^15^[2]. Although the vast majority of sequence data is dedicated to exploring genomes, transcriptomics is growing fast[3]. This increase correlated with increased availability of high performance *de novo* transcriptome assemblers. Given these developments, there is a growing need for standardization of benchmarks and metrics used to evaluate transcriptome assemblies.

RNA-Seq has been leveraged with *de novo* transcriptome assembly to learn about plant innovations including parasitism[4–7] and C4 photosynthesis[8], plant processes including fruit ripening[9], drought response[10], and flavonoid biosynthesis[11], chemical defenses[12], and the evolution of sex chromosomes[13]. The recent boom of RNA-Seq studies involving *de novo* assembly has motivated innovations in assemblers developed specifically for RNA-Seq data (Velvet[14, 15], Oases[16, 17] (includes Velvet[18]), SOAPdenovo[19–29], SOAPdenovo-Trans[30], CLC[31], ABySS[32], Trinity[5, 13, 33–38]). Comparison of *de novo* transcriptome assembler performance is hindered by lack of widely used standard quality metrics[39] or rigorous evaluation of a comprehensive selection of assemblers with a transcriptome from a high quality reference genome. Reference-dependent metrics have been proposed that include accuracy, completeness, contiguity, chimerism and variant resolution[40] which, in large part, rely upon arbitrary thresholds to inform assembly quality (e.g. the number of genes covered >80%). Often the length statistics reported for *de novo* assemblies reveal that the number of unigenes (singletons, contigs and scaffolds—collectively “unigenes”) is far greater than the expected number of transcripts and that the total length of the assembly (total number of bases) also differs from the expected transcriptome length. Evaluation of an assembly output typically includes: 1) N_50_ (the unigene length at which the cumulative assembled base pairs reaches 50% of the total assembly length), 2) the number of unigenes (n) greater than length *x*, and/or 3) the proportion of sequencing reads that map back to an assembly. While these can be informative statistics for relative comparisons, individually they fall short of adequately informing absolute quality. For instance, a high rate of mis-assembly resulting in chimeras could inflate the N_50_ and length statistics. Similarly, a high rate of non-assembly of low-moderate abundance transcripts may inflate the N_50_ and length statistics.

Estimation of assembly success by reporting the relative frequency of hits to expected sequences (e.g. conserved gene sequences in external databases) by itself can be rather arbitrary and fails to inform how well the assembly represents the data and thus the transcriptome, especially when the search parameters are not consistent from study to study. Reporting the recovery of one or more conserved sets of genes has been used[7, 34, 41–46] as a metric for sampling effort and assembly success, but the extent to which conserved genes serve as a proxy for the whole transcriptome remains unknown. Transcript annotation efforts are largely aimed at reporting the number of unigenes with hits in an external database such as NCBI’s non-redundant protein sequences database[2] (NR), the Kyoto Encyclopedia of Genes and Genomes[47] (KEGG), Swiss-Prot[48], Clusters of Orthologous Groups of proteins[2] (COG) and The Gene Ontology[49] (GO). Sequence comparisons against these databases could elucidate chimeras but there are no established criteria for using alignment metrics to evaluate assembly quality.

Here we describe an in-depth comparison of 99 assemblies (software in Table 1) of the *Arabidopsis thaliana* leaf transcriptome leveraging the 10^th^ generation *Arabidopsis* genome as ground truth. Our analysis focuses on data produced by Illumina sequencing technology for 3 reasons: 1) this technology contributes the majority of new data to NCBI’s sequence read archive, 2) the cost per base provides a clear price advantage over other technologies and 3) many of the current *de novo* assemblers are designed to assemble Illumina data. We also evaluated the accuracy of measurements of expression levels extracted from *de novo* assemblies by interrogating the same RNA samples with NimbleGen Multiplex microarrays and qRT-PCR.

**Table 1:** Summary of assembly software.

Assembler ID	Version	Reference/URL
**Mosaik**	Mosaik Assembler (v1.1.0014)	http://bioinformatics.bc.edu/marthlab/Mosaik
**Trinity**^‡^	Trinity (release 3122011 and release 01132014^§^)	http://trinityrnaseq.sourceforge.net/[50]
**Inchworm**	Trinity (release 3122011)	
**CLC**	CLC Assembly Cell (v3.2)	http://www.clcbio.com/
CLCscaf*^‡^	CLC Assembly Cell + Scaffolding (v4.0.6)	
**Oases**	Oases (v0.1.22)	http://www.ebi.ac.uk/~zerbino/oases/[53]
**Velvet**	Velvet (v1.1.03)	
**SOAPdenovo**	SOAPdenovo (v1.04)	http://soap.genomics.org.cn/about.html[54]
SOAPtrans*^‡^	SOAPdenovo-trans (v1.03)	
**ABySS**	Trans-ABySS (v1.3.0)	http://www.bcgsc.ca/platform/bioinfo/software/abyss[55]
**NG MO**	NextGENe (v2.17)	http://www.softgenetics.com/NextGENe_9.html
**NG IT**		

*Used to assembly Illumina biological replicate 1 only

^**§**^Used to assemble Rice and subsampled Arabidopsis

^‡^ Current versions of these assemblers implement the same core assembly algorithm as those tested here (personal communication Dr. Brian Haas, http://www.clcbio.com/, and http://soap.genomics.org.cn/)

The results of these analyses reveal that Trinity[50], CLC[51] and SOAPdenovo-trans[30, 52] assemblers have superior performance. We analyzed primary assemblies as well as those that were post-processed by a series of steps akin to the Trinity pipeline that yielded improved key assembly metrics. We introduce *SCE*RNA, a collection of versatile post-processing tools and protocols for SGS transcriptome data that improve the quality of *de novo* transcriptome assemblies.

## Results

### Transcriptome data and reference genome

#### Read mapping reference and data summary statistics

The TAIR10 annotation of the *Arabidopsis thaliana* genome was used as the reference for mapping of reads from replicate young-leaf RNA samples. To reduce variation from multiple RNA samples, we generated two large RNA pools (~1 mg) that were used exclusively in all analyses presented here. *Arabidopsis* cDNAs were refined to include only the longest splice variants for detected transcripts (25,512 genes—the “detected gene set”), which includes 82.4% of the 27,206 nuclear, protein-coding *A*. *thaliana* genes. S1 Table contains a summary of sequencing and read alignment statistics for all RNA-Seq libraries used in this study. A normalized Illumina library produced 6.4 Gbp in ~27 million 120 bp paired-end reads. The replicated non-normalized Illumina libraries each produced ~4.2 Gbp represented by ~27 million 76x76 bp paired-end reads.

#### *Arabidopsis* gene tagging and cDNA coverage

**Fig 1:**
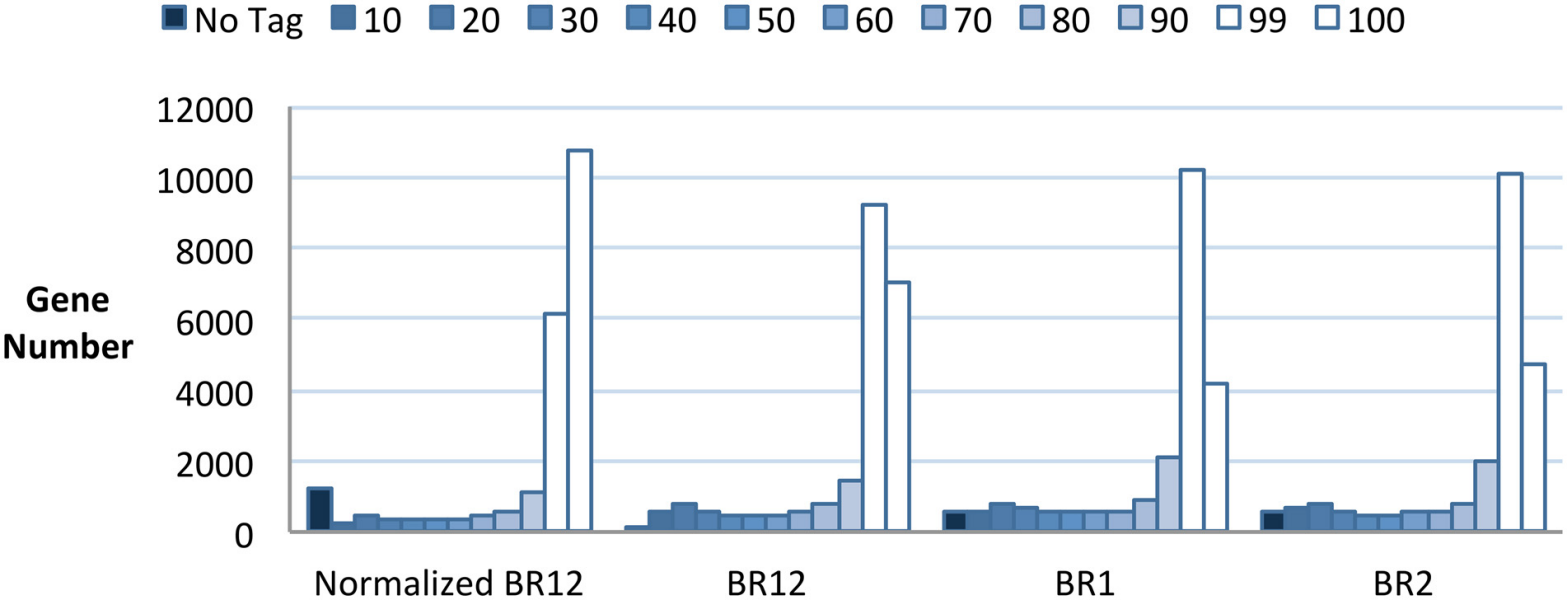
Illumina Sequence coverage of *Arabidopsis* cDNAs. Coverage of all detected genes by sequencing reads. The darkest bar represents the number of detected genes not tagged, and each progressively lighter bar represents genes in a bin with a 5% increase in coverage, with the two lightest bars showing the number of genes covered at >90% and >99%, respectively. Normalized BR12 = Normalized Illumina library from pooled biological replicates 1 and 2, BR12 = combined coverage of Illumina biological replicates 1 and 2, BR1 = Illumina biological replicate 1, BR2 = Illumina biological replicate 2.

The coverage of each *A*. *thaliana* gene (see S2 Table for definitions of assembly metrics) was determined by mapping quality-trimmed reads to the longest splice variant of the detected TAIR10 cDNAs. Fig 1 shows the distribution of cDNA read-level coverage for each of the sequencing data sets in this study. Average cDNA coverage was highest in the Normalized Illumina library at 79.7%. The average coverage from the non-normalized Illumina libraries was 76% (±0.21), or 80.7% when combined. The greatest average cDNA coverage was obtained from the combination of the two non-normalized Illumina libraries. Although the normalized Illumina transcriptome had the greatest proportion of high coverage cDNAs (at 99–100% coverage) it also lacked reads for a large number of transcripts that were detected in one or more of the other libraries (Fig 1). In contrast, the combined non-normalized datasets (BR12) captured reads for nearly every gene in the detected gene set.

We predicted the sequencing statistics of the non-normalized Illumina biological replicate one (BR1) data set using ESTCalc at http://fgp.huck.psu.edu [56]. This simulation indicated that BR1 would provide tags for all expressed genes. We found that the non-normalized BR1 library (4.2 Gbp of sequence data) generated tags for 96.2% of the detected gene set. Doubling the sequence data amount with Illumina BR2 tagged an additional 966 genes, bringing the total to 99.9% of detected genes tagged. Since mean transcript coverage was not substantially increased in the BR12 combined dataset, BR1 was deemed sufficient to represent the transcriptome for most practical purposes and unless noted, was used for all analyses described below.

#### Assembly reference definition and data choice for *de novo* assembly

The TAIR10 cDNA reference-based assembly (with Mosaik-*SCE*RNA–see methods) of BR1 contained unique assembled unigenes that represented 20,176 cDNAs (79.1% of the detected gene set). 17,723 (87.8%) of these transcripts were covered >50%. 5,336 (20.9%) of the detected cDNAs were not represented in the Mosaik assembly, indicating insufficient cDNA coverage (e.g. low expression) to support even a reference-based assembly. When we included a second data set of equivalent size and quality (Illumina BR2), the number of Mosaik unigenes increased to 20,840 (81.7%). The number of assembled transcripts covered >50% increased to 18,596 (89.2%), while the number of missing genes decreased to 4,672 (18.3%).

An important distinction between the cDNA reference and *de novo* assembly reference is between detection and reconstruction, respectively. While we *detected* 25,512 genes, the number of reconstructed genes (>50% coverage) is closer to 18,000, as indicated by the number of genes represented in our reference-based transcriptome assemblies of BR1 with Mosaik.

### Evaluation of *de novo* assembly via a reference assembly standard

#### Quality metrics and terms for comparison of *de novo* assembler outputs

We developed a comprehensive set of quality metrics for transcriptome assembly (Table 2) that includes reference-dependent and reference-independent metrics. These metrics were used to evaluate so-called “primary” assemblies (most basic output of a given assembler) and “post-processed” assemblies that represent refined assembly outputs (S2 Table). We have also included a select set of informative analyses of *de novo* assemblies generated with updated versions of CLC (CLCscaf), SOAPdenovo (SOAPtrans) and Trinity (see Table 1 and Materials and Methods).

**Table 2:** Summary of assembly quality metrics used in this study.

Assembly Metric	Description
**Assembled**	
**Sequence Count**	Number of assembled sequences—i.e. unigenes
**Median Length**	Median length of unigenes
**Maximum Length**	Maximum length of unigenes
**N**_**50**_ **Length**	50^th^ percentile unigene length
**N**_**50**_ **Mbp**	50^th^ percentile mega base pairs
**N-content**	Number of ambiguous base pairs
**RNA-Seq SCC**	Spearman’s Rank Correlation Coefficient with Mosaik assembly
**RNA-Seq PCC**	Pearson’s Correlation Coefficient with Mosaik assembly
**Mappable Reads**	Number of quality trimmed reads that map to an assembly (also expressed as %)
**Mismatch Error (%)**	Number of base call errors (% of bases called incorrectly)
**Alignment Gap Rate**	Alignment gap rate—missing bases
**Error**	(% of unigenes with alignment gap errors)
**Type I Coverage**	
**Gap Error—Case I**	Number of internal coverage gaps (noncontiguous assembly)
**Type I ISO Error –**	Insufficient Overlap—number of instances of non-assembly with
**Case II**	alignment overlap ≤(*kmer– 1)*
**Type II Error–Case III**	Conflict Overlap—number of instances of non-assembly with alignment overlap >(*kmer– 1)*
**Type II Error**	Ambiguity error—assembled sequence has “best hits” to multiple genes (Cases I-V see S1 Fig)
**No Hit**	Number of “detected genes” not represented by an assembled sequence
**Coverage**	Percentage of cDNA covered by an assembly, not limited to a single assembled sequence, cumulative
**Normalized Bit**	Normalized Quality Metric—bit score normalized for sequence
**Score—BS**	length, indicator of long and accurate unigenes—penalties: mismatch -2, gap -5
**Sequenced**	Normalized Sequencing Depth Metric—The number of sequenced
**Fragment/bp—SFB**	fragments (orphans or pairs) that map to a unigene, normalized for sequence length

#### Primary assembly statistics

Primary assemblies are the most basic, or minimal, output of a given assembler (Table 3). Compared to the Mosaik assembly, the primary assembly statistics display variable patterns of performance. For instance, all of the assemblies have a greater number of assembled unigenes than the Mosaik assembly, and many are larger (N_50_ Mbp), suggesting fragmented and duplicated transcripts. The N_50_ lengths are all below Mosaik’s 1,838 bp, indicating *de novo* vs. reference-guided assembly results in fewer full-length sequences, or an inflation of small subsequences. Some assemblies (SOAPdenovo, Velvet, ABySS) garner a small proportion of mapped reads, indicating that these *primary* assemblies may be less representative, highly fragmented, or especially incomplete compared to the Mosaik standard. The assembly with the number of unigenes most similar to Mosaik is NG IT. This assembly has good statistics (see unigene count, N_50_ length) but is the second smallest assembly and middle of the pack in terms of RNA-Seq correlations (RNA-Seq SCC and PCC with Mosaik, see Table 3), suggesting a less complete assembly. Yet, the NG IT primary assembly garners the greatest proportion of mapped reads. The CLC primary assembly is one of the larger assemblies (N_50_ Mbp), but has the lowest N_50_ length and the most unigenes while having outstanding RNA-Seq correlation statistics. Together this indicates the CLC assembly is highly fragmented yet highly representative of the input data. The Inchworm assembly has excellent length statistics and good RNA-Seq correlations, yet it has >50,000 unigenes, which is ~2.5 times the number of unigenes found in the Mosaik assembly. These conflicting patterns suggest that the output of each assembler is quite variable and that simple length and read-mapping statistics do not sufficiently describe assembly quality. Recent variants of CLC (CLCscaf), SOAPdenovo (SOAPtrans) and NG MO (NG IT) are improvements, and show that additional steps beyond those implemented with basic De Bruijn graph methods have value.

**Table 3:** Assembled sequence and RNA-Seq statistics for assemblies of Illumina biological replicate 1 (BR1).

	Assembled Sequences Count	Median Length	Max. Length	N_50_ Length	N_50_ Mbp	N-content	RNA Seq SCC	RNA Seq PCC	% Reads Mapped
**Primary Assemblies**									
**Mosaik**	20,930	1,326	16,339	1,838	15.57	1,771,804*	1	1	71.7
**Inchworm**	51,896	435	15,057	1,622	21.83	0	0.95	0.93	81.0
**CLC**	161,183	135	15,057	578	22.04	7,022	0.96	0.95	65.0
**CLCscaf**	42,265	313	12,532	1,421	14.73	47,268	0.95	0.93	68.7
**Velvet**	37,357	629	13,061	1,582	17.85	12,748	0.93	0.91	35.4
**SOAPdenovo**	44,537	289	15,247	1,322	14.09	304,230	0.87	0.87	24.9
**SOAPtrans**	37,469	426	21,355	1,557	15.11	515,984	0.93	0.92	65.9
**ABySS**	28,362	601	14,818	1,474	12.58	1,026	0.92	0.89	34.8
**NG MO**	30,880	232	7,391	877	7.12	0	0.88	0.81	75.8
**NG IT**	24,137	602	12,307	1,326	10.51	0	0.93	0.89	82.6
**Post Processed Assemblies**									
**Mosaik-S**	20,178	1,361	16,339	1,848	15.4	1,707,111*	1	1	71.0
**Inchworm-S**	34,812	584	15,057	1,560	15.76	0	0.95	0.93	80.2
**Trinity-ICB**	30,086	543	15,057	1,552	13.25	0	0.90	0.87	75.5
**CLC-S**	81,734	153	15,057	1,114	16.63	179	0.96	0.94	66.6
**CLCscaf-S**	32,290	509	12,532	1,511	13.7	1,809	0.95	0.93	68.5
**Velvet-S**	26,707	729	13,061	1,561	13.4	147	0.91	0.88	34.1
**Oases-VO**	22,503	722	13,061	1,579	11.36	122	0.82	0.78	28.1
**SOAPdenovo-S**	32,515	472	15,175	1,427	12.86	10,040	0.84	0.81	25.4
**SOAPtrans-S**	27,260	662	21,355	1,622	13.23	402,496	0.91	0.89	63.7
**ABySS-S**	24,930	717	14,818	1,507	12.1	15	0.91	0.87	34.1
**NG MO-S**	27,971	255	7,406	912	6.85	0	0.88	0.81	73.5
**NG IT-S**	22,783	640	12,307	1,344	10.24	0	0.93	0.89	82.0

Unigenes shorter than 100 bp were removed. N-content refers to the number of bases in unigenes that were ambiguous. Trimmed and filtered reads were mapped to the respective assemblies and percentages were calculated by dividing by the total number of reads from BR1 that mapped to TAIR10 cDNAs. RNA-Seq correlations used respective Mosaik assemblies as a reference. SCC = Spearman’s rank correlation coefficient and PCC = Pearson’s correlation coefficient.

*includes gaps in cDNA reference coverage

#### Post-processed assembly statistics

**Fig 2:**
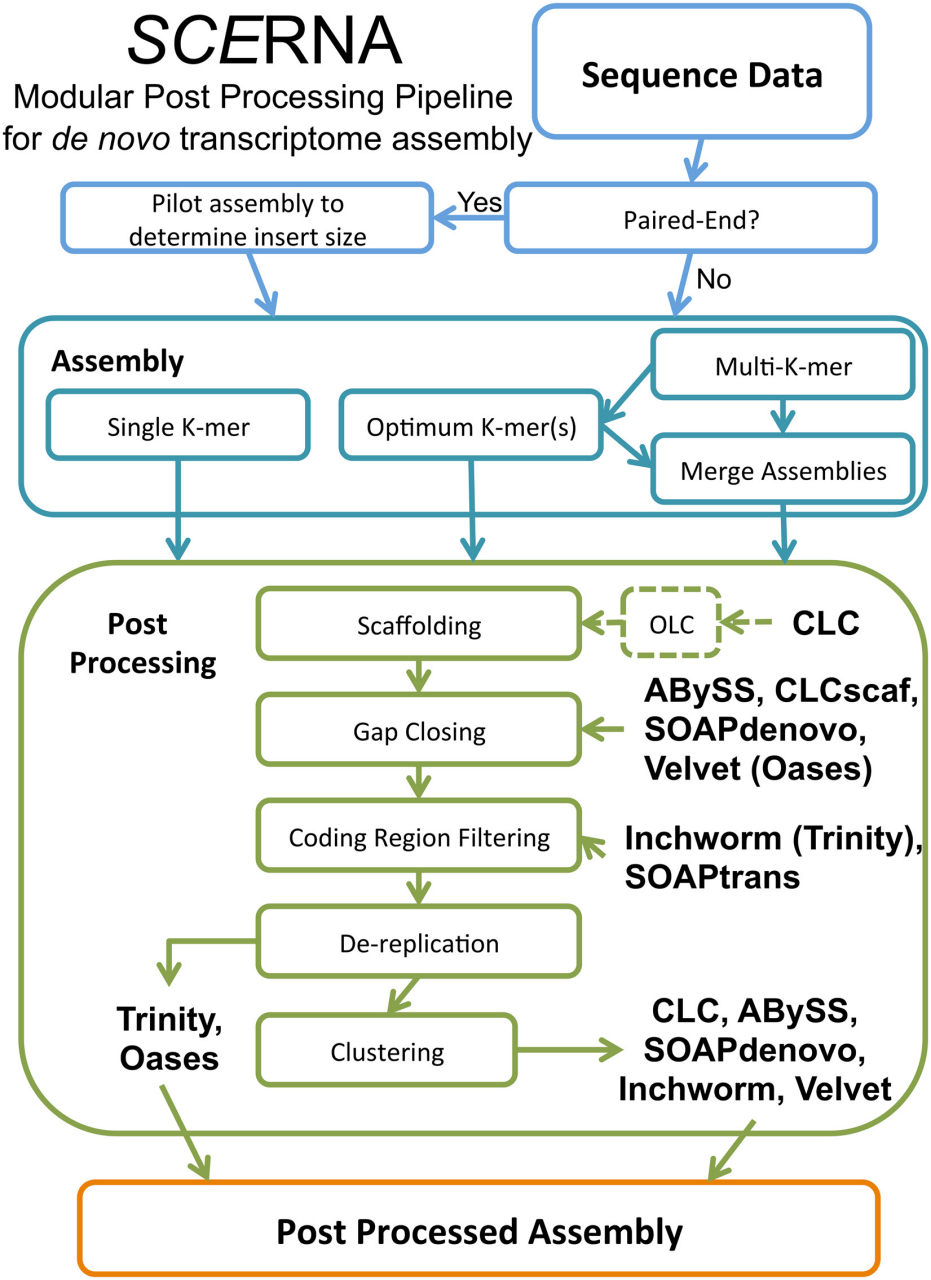
*SCE*RNA Flowchart. *SCE*RNA stands for Scaffolding and Error correction for *de novo* assemblies of RNA-Seq data. This collection of post-processing tools allows flexible implementation at various steps post assembly and with multiple assemblers and data types.

The goal of post-processing is to improve key aspects of an assembly (e.g. length-statistics and unigene counts) without diminishing other important features such as the proportion of mappable reads (e.g. for *de novo* RNA-Seq). In Table 3 the landscape of assembly statistics appears much more even after refinement by post-processing with the Velvet-Oases, Inchworm-Chrysalis-Butterfly (Trinity), or our modular *SCE*RNA (Fig 2) post-processing tools. The similarity is greater when length cutoffs are imposed (S2 Fig), revealing comparatively large differences between the assemblies that are contained in short rather than long unigenes. The number of unigenes was reduced in all assemblies by *SCE*RNA (Table 3), and the length statistics are generally improved, some dramatically so. Even the Mosaik reference-based assemblies were improved with SCERNA post-processing. The RNA-Seq correlations were not substantially changed nor were the proportions of mappable reads. For a comprehensive summary of post-processing effects on all *Arabidopsis* assemblies see S1 File. All assemblies are improved by post-processing, though as expected, none of the *de novo* assemblies are as good as Mosaik-S assemblies of the same data.

### Evaluation of *de novo* assembly via a genome (cDNA) standard

#### Error rates

The error statistics of each primary and post-processed assembly reveal the occurrence of fragmented, incomplete or incorrect assemblies. By estimating various types of error we can begin to explain the differences of each *de novo* assembly compared to the *A*. *thaliana* cDNA reference. Base call error rates were <0.5% in all post-processed assemblies and base call error rates were improved (or nominally changed) by post-processing (S1 File). The proportion of unigenes with alignment gaps (to TAIR10 cDNAs) ranged from 0.001% for Mosaik-S to 0.005% for Inchworm-S, Velvet-S and CLC-S.

We reported two key error types that inform assembly failures. Type I errors[57] indicate failure to assemble (fragmentation) and Type II errors[57] indicate annotation ambiguity (with a subcategory, Case I, that is most indicative of chimerism) (S1 Fig). The number of transcript assembly gaps between 2 unigenes (Type I Case I) ranged from 16,931 for Mosaik-S to 8,964 in Oases-VO. Type I Case II counts (overlap ≤*kmer*-1) were very low in Mosaik-S at 56 and ranged from 5,637 in CLC-S to 1,227 in ABySS-S. Fragmentation that occurs despite an overlap >*kmer*-1 (Type I Case III) was also low in Mosaik-S at 256 and higher in the *de novo* assemblies ranging from 7,819 in CLC-S to 1,381 in Oases-VO.

Type II error occurs when a unigene cannot be unambiguously assigned to a single reference cDNA. This may indicate that a unigene matches two closely related loci, or that an assembly error has occurred resulting in a chimera. We first examined pairs of closely related genes because these may be likely to be misassembled due to high sequence similarity. We plotted the Ks values for gene pairs (reciprocal best hits) found in each assembly plus those found in our detected gene set and TAIR10 (S3 Fig). The Mosaik-S assembly plot showed a lack of pairs with low Ks values compared to the plot for the detected gene set, indicating that *closely related* gene pairs were preferentially excluded. To determine if the difference was due to sampling bias (in which one mate of the pair is expressed at a level sufficient for assembly and the other is not) or due to limitations of assembly (highly similar reads from closely related gene pairs are ambiguous) we examined the read count statistics for the 2,080 gene pairs lacking from the Mosaik-S plot relative to the detected gene set plot. For the majority of missing gene pairs (1,607, ~75%) there were insufficient reads for assembly, or only sufficient unique reads (SFB ≥0.1; Table 2) for one mate of the pair, and these genes tended to have higher Ks values among the missing genes (S4 Fig). This is consistent with previous work indicating that older paralogous gene pairs (higher Ks) are less likely to be co-expressed than younger paralogous gene pairs (lower Ks) [58–62]. 473 expressed gene pairs (EGP) had sufficient read depth for assembly and provided us with a list of co-expressed genes with high sequence similarity.

The instance of successful assembly (BS >1.5; See Table 2) of both mates of the EGPs was very low for Mosaik-S at only 0.8% (4 EGPs, S5 Fig). Roughly half of the EGPs were represented by both mates in the Mosaik-S assembly, though ~74% of these were very short (25% length). Generally, the *de novo* assemblies contained about the same number of high quality pairs with Inchworm-S and Velvet-S at 1.5% to NG MO-S and Trinity-ICB at 0.6%. The primary difference was in the total number of high quality (>1.5BS) unigenes. All *de novo* assemblers produced more high quality EGP unigenes than Mosaik-S. All together, this produced a reciprocal pattern of “hit/no hit” (S5 Fig) of EGP genes in the *de novo* assemblies. Between 66–76% of the EGPs were represented by only one mate of the pair in all *de novo* assemblies. This pattern was far less pronounced in the Mosaik-S assembly with multiple low quality unigenes that represent more pairs, though fewer total EGP genes. This complex pattern shows that *de novo* assemblers tend to assemble higher quality unigenes representing one mate, while Mosaik-S assembles lower quality unigenes that represent more pairs. Taken together with the very low Type II error rates for most of the assemblies, this analysis of the closely related genes strongly suggests that the instance of true chimeric unigenes is very low. Thus it seems that loci whose shared sequence is *less than* the read length are resolved accurately while traversing the graph with reads, and loci whose shared sequence is *greater than* the read length seem to resolve with only a single accurate unigene (one of the pair), not a chimera.

We next examined the global occurrence of Type II errors and subdivided Type II errors into 5 cases (S1 Fig). In our analysis Type II Case I represents a special case that was most reliable in identifying chimeric unigenes compared to cases II-V. In Case I, the matching regions (e.g. two distinct loci) do not overlap, indicating a potential false join, whereas in cases II-V the matching regions overlap, indicating ambiguity between two loci. We excluded Type II errors reported for adjacent genes based upon a detailed analysis of Type II Case 1 errors in BR1 assemblies (See S2 File). We verified that adjacent genes were accurately co-assembled (one unigene with 2 full length open reading frames (ORFs)) by aligning unigenes to the *Arabidopsis* genome. All cases of Type II Case 1 error reported for adjacent genes in assemblies of BR1 were simply co-linear or overlapping genes with excellent full-length alignments to the *Arabidopsis* genome (S2 File). These are cases of co-assembly and do not represent assembly failure, but rather limitations of the data (i.e. not strand specific) and the relative position and orientation of genes in the genome. Furthermore, post processing will easily report two distinct open reading frames (ORFs) for such co-assembled genes, therefore removing any ambiguity. Type II Case 1 errors of non-adjacent genes were often ambiguous alignments to closely related or duplicated genes (S2 File) and were not chimeric. In fact, unambiguous cases of chimeric unigenes were exceedingly rare, with a substantial portion of Type II Case 1 errors reported consisting of what appear to be trans-spliced transcripts (S2 File).

The Type II error rates for post-processed assemblies of BR1 were all 0.0–0.07% except the highly fragmented CLC-S assembly at 0.26% (S1 File, and S3 Table). The Trinity-S Type II Case I error count was 4, the count for Mosaik-S was 3 and NGMO-S had only 1. The highest number of Case 1 assembly errors in post-processed assemblies of BR1 was in CLC-S at 56, yet this was still only 0.07% of all unigenes in the assembly. The proportion of Type II Case I errors were consistently less than half of all Type II errors, with many much lower. This indicates a rate of chimeric assembly that is very low across all assemblies and an order of magnitude lower than a previous estimate[30]. Our annotation strategy relied on BLASTn alignments to TAIR10 cDNAs and the majority of Type II errors are likely due to ambiguous unigene alignments rather than erroneous assemblies (S2 File).

#### cDNA coverage statistics in *de novo* assemblies

The average *sequencing read*-level cDNA coverage of the detected gene set in BR1 was 75.8%, while the average *unigene*-level cDNA coverage of the Mosaik assembly of BR1 was 65.3%. While we estimated coverage of cDNAs (mature mRNA transcripts) in an attempt to learn about the accuracy and efficiency of reconstruction of the input RNAs, it is important to note that the coverage of the coding sequence (CDS) in each transcript is very likely to be greater than, or equal to, the cDNA (mature transcript) coverage. As expected, all primary and post-processed *de novo* assemblies yielded poorer coverage statistics than the respective TAIR10 cDNA reference-based Mosaik assembly. The differences among all *de novo* assemblies were not dramatic and illustrate a subtle gradation in terms of completeness (S6 Fig). Inchworm-S and CLC-S had the best unigene-level coverage statistics, yet were only slightly better in coverage than other assemblies which still yielded many thousands of unigenes that, cumulatively, covered reference transcripts >75%.

### The complex assembly landscape

#### Integration of quality metrics

**Fig 3:**
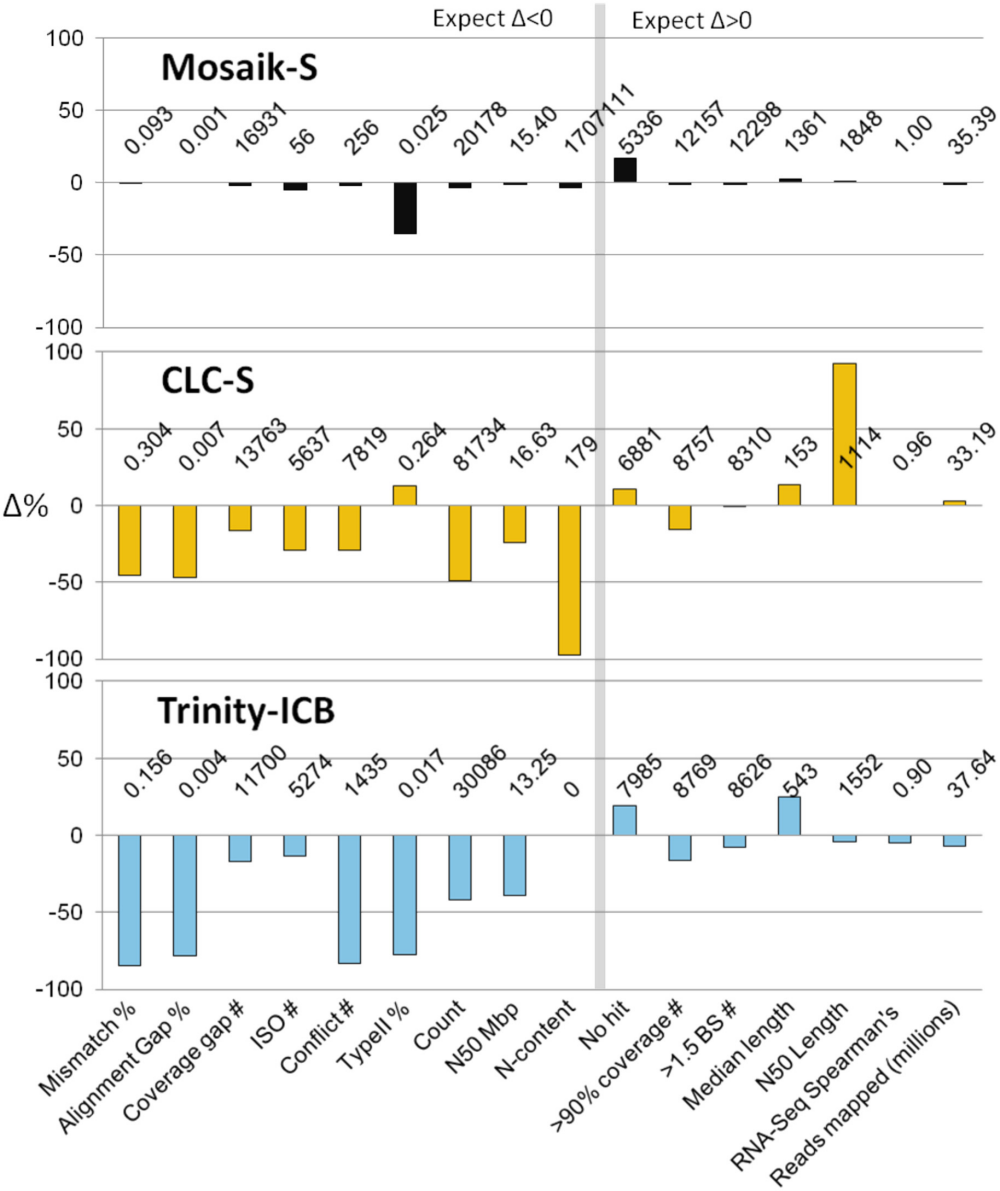
Post-processed assembly delta plot, showing the effect of post-processing in several assembly quality categories. The histogram shows the magnitude (% change) and direction of change in each category for the Mosaik-S, CLC-S and Trinity-ICB assemblies of Illumina biological replicate 1. The post processed values in each category are printed above the x axis for each assembly. A vertical line separates categories where an assembly improvement would result in a decrease in the respective measure (“Expect Δ<0” categories, left of line) or an increase in the respective measure (“Expect Δ>0” categories, right of line).

The beneficial effects of post-processing tools like those integrated into Velvet-Oases, Trinity and *SCE*RNA (Fig 2) are seen in all assembly metrics presented thus far. In Fig 3 we summarize the effect of *SCE*RNA post-processing on the Mosaik reference assembly and CLC, plus the effect of the Trinity pipeline on the intermediate output generated with Inchworm. The direction of change varies for certain categories. For instance, we expect that successful post-processing should reduce error rates and unigene numbers, but increase length statistics. These changes would indicate error correction and consolidation of information resulting in a smaller, more concise, lower error and more contiguous assembly. A summary of the effect of post-processing on assemblies is presented in S1 File.

The Mosaik assembly is generated by aligning reads to the reference set of cDNAs, thus the effect of post processing should be minimal because each unigene is reference checked during assembly. Very complete yet highly fragmented assemblies like CLC should benefit greatly from post-processing, while high performance transcriptome assemblers like those implemented in the Trinity suite should show more modest gains during post-processing. The effect on the Mosaik assembly is minimal with the exception that the Type II error rate is reduced to 0.025% and the number of genes missing from the assembly was slightly increased. The effect on the CLC assembly was pronounced with substantial improvement of error rates, yet small changes in length statistics and unigene count. The changes for the Trinity assembly from the intermediate Inchworm output were more modest, and generally showed improvement in all categories.

#### Visualizing the complex assembly landscape

Despite a rigorous examination of numerous assembly quality metrics, none completely capture the key differences among the assemblies. Assemblers like CLC produce very comprehensive, yet highly fragmented transcriptome assemblies. Others like Trinity produce concise assemblies by excluding portions of the transcriptome that are difficult to resolve, as evidenced by the lower error rates and relatively greater number of missing genes. It is easy to make comparisons and see the effect of post-processing in individual categories, yet the fate of each gene needs to be considered to draw conclusions about transcriptome-wide assembler performance.

To track the fate of each gene in an assembly, we integrated our read mapping data (sequenced fragments/basepair—SFB) and BLAST-based sequence identification (bit score/basepair—BS) to reveal how the quality of *de novo* transcriptome assembly changes across the dynamic range of expression in the *A*. *thaliana* young leaf transcriptome. Our approach selects a single unigene with the highest BLASTn alignment bit score to a given TAIR10 cDNA, normalizes this score by cDNA length and then plots it against the normalized read depth (SFB). The ideal BS (alignment bit score normalized for sequence length) is 2.0 for a perfectly matched alignment. However, the BS for even a perfectly matched alignment may be slightly less than 2.0, due to variations in nucleotide base frequency, and thus the likelihood of transitions at a given position[63]. For example, a unigene that aligns to a 1000 bp transcript with 2 BS and 0.2 SFB is represented in the assembly by a unigene identical to the reference, and 200 sequenced fragments (pairs and/or orphans) map to the unigene. In this example, the average depth of sequence is roughly 30x, though we caution against the use of “depth of sequence” as a descriptor of transcriptome data sets, since read depth across genes and even across the length of individual reference transcripts varies by orders of magnitude[64].

**Fig 4:**
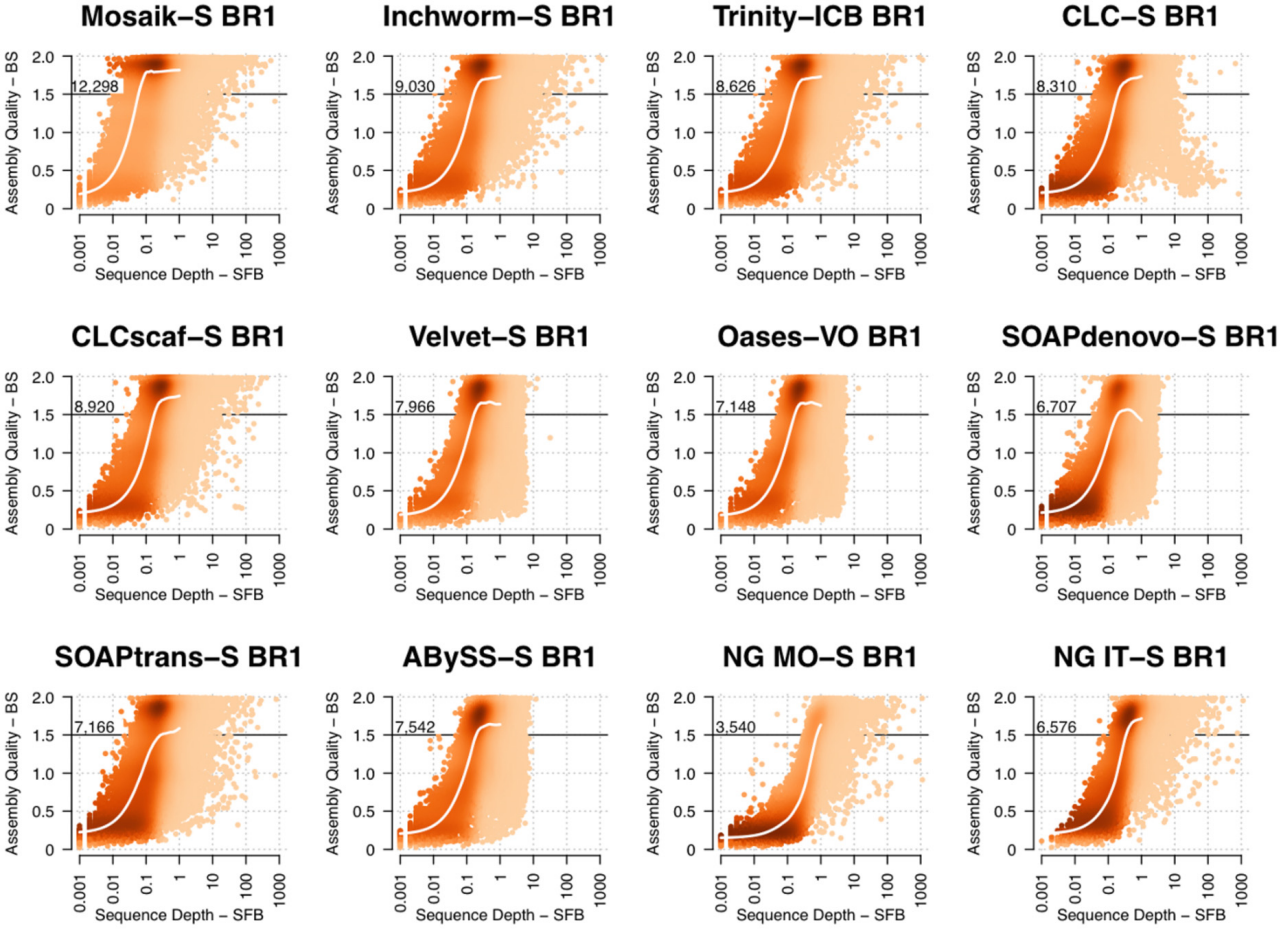
The quality of unigenes as a function of sequencing depth for Illumina biological replicate 1. The units for “Assembly Quality” are Normalized Bit Score (BS, maximum of 2) and the units of “Sequence Depth” are Sequenced Fragments/bp (SFB). The number printed in the plot area is the number of unigenes with normalized bit score above 1.5. A BS of 1.5 is an arbitrary threshold, yet represents long and accurate assemblies (75% length, high accuracy), and is used to illustrate the difference in the high-density region seen in most plots near BS 1.75–2.

In Fig 4 the results of this integrated approach are presented for all post-processed assemblies of BR1. Lowly expressed genes are represented in all assemblies by low quality (i.e. incomplete) unigenes. The accumulation of reads results in a rapid increase in unigene quality from 0.05 to 0.1 SFB for the Mosaik-S assembly, indicating that for an average gene (1000 bp), 50 76x76 bp paired-end Illumina reads is a practical lower limit for full length and accurate reference-based assembly.

The read depth threshold for high quality unigenes is greater for *de novo* assembly. With the exception of the NG MO-S assembly, the read use efficiency at lower sequencing depths was more or less equivalent among assemblers, with a sequencing depth between 0.1 and 1 SFB routinely producing long and accurate unigenes (Fig 4). To further reveal read-use characteristics of each assembly, a white trend line is plotted in each graph in Fig 4. This trend line shows the normalized read depth inflection point at which an assembly accumulates more contiguous and accurate unigenes. Except for NG MO-S, the trend of increasing quality as read depth increases is highly similar, with the most dramatic differences seen above 10 SFB. This analysis shows that most *de novo* assemblers rapidly accumulate long and accurate unigenes at 0.1–0.2 SFB, indicating that for an average 1,000 bp transcript, 100–200 reads (76x76 bp paired end) is a practical lower limit for successful *de novo* assembly. Generally, for genes in the 2^nd^ and 3^rd^ quartile of expression level, the assemblies are indistinguishable, yet the differences in the 1^st^, and especially the 4^th^, quartile of expression level are dramatically different.

Surprisingly, increasing sequence depth does not result in high quality assembly for all genes. This counterintuitive pattern is illustrated in Fig 4 as a persistence of *lower* quality unigenes at a normalized read depth above 0.1 SFB. The assemblies CLC-S, Oases-VO, Velvet-S, SOAPdenovo-S and ABySS-S all fail to assemble highly expressed transcripts at >10 SFB (>10,000 reads for 1,000 bp transcript), while more robust assemblers like Inchworm-S, Trinity-ICB, CLCscaf-S, SOAPtrans-S, NG MO-S and NG IT-S continue to efficiently produce unigenes for highly expressed transcripts, though some of these are of lower quality. This surprising pattern is also seen in primary assemblies (S3 File) and is not a result of post-processing.

These results lead to the unexpected finding that the most highly expressed genes may be entirely missing from some *de novo* assemblies (Velvet-S, Oases-VO, SOAPdenovo-S, ABySS-S). In contrast the Inchworm-S, Trinity-ICB, CLCScaf-S, SOAPtrans-S, NG IT-S and NGMO-S are resistant to assembly failure at high read depth. NGMO-S is inefficient, yet the implementation of an iterative approach (NG IT-S, designed to control for extreme sequencing depth) substantially improves assembly efficiency and contiguity (Fig 4). That the CLC-S assembly failed at high sequencing depth, but still has an excellent RNA-Seq correlations and high reference cDNA coverage suggests that many of the fragmented transcripts are from highly expressed genes.

To illustrate the challenge of distilling tremendous variation into concise unigenes representing a single locus, we aligned unigenes from 4 assemblies of BR1 (Inchworm, Trinity-ICB, CLC and CLC-S) with the genomic DNA, cDNA and CDS of AT1G31330.1, which encodes the highly expressed Photosystem I Subunit F (S7 Fig). In this extreme case the SFB of this gene in BR1 was 128 (>121,000 reads). The Inchworm primary assembly produced two unigenes that matched the AT1G31330.1 cDNA. The post-processed Trinity-ICB assembly produced a single perfect unigene that extended the 3’ and 5’ UTR’s by 72 bp and 74 bp, respectively. In contrast, the primary CLC assembly produced 623 distinct unigenes that aligned to the AT1G31330.1 cDNA. Even after post-processing, the CLC-S assembly contained 96 unigenes that aligned to AT1G31330.1. The alignments of CLC unigenes reveal numerous single base-pair and structural differences including a unigene that contains the intron (S7C and S7D Fig). Depending upon research goals, it may be desirable to examine the numerous differences for AT1G31330.1 displayed in CLC assemblies or, alternatively, extract a single perfect unigene from another assembly for downstream analysis.

### Is normalization a useful strategy to improve transcriptome assemblies?

Our analysis of the *de novo* assemblies reveals that the dynamic range of transcript abundance is a primary hurdle to full-length and accurate transcript assembly, often resulting in missing or fragmented transcripts. Normalization via Duplex Specific Nuclease (DSN) is designed to enzymatically reduce the frequency of the most abundant transcripts and should directly address assembly errors arising from extreme sequencing depth for highly expressed genes. An important distinction between DSN normalization and e-normalization (like that offered by Trinity[50]) is that the former reduces the abundance of highly expressed transcripts, while the latter is a strategy to reduce memory requirements during graph construction by reducing the frequency of highly abundant k-mers. Since the failure of *de novo* assemblies seems to be driven by extreme read depth it is unlikely that e-normalization will impact Type II error rates. Manual digital normalization, like that implemented in NG IT[4], improves assembly but is extremely labor intensive and therefore not practical.

The normalized Illumina transcriptome provided the highest average coverage for any single dataset (Fig 1), yet also lacked any reads for >2000 of our detected genes, more than double the number missing from BR1 or BR2. This was unexpected since the process of normalization removes highly abundant transcripts thus *increasing* the likelihood of detecting less abundant ones. The result should be a *more* diverse library along with an increase in average transcript coverage. We hypothesized that this apparent dichotomy was due to the removal of lowly expressed genes along with highly expressed and very similar relatives; this could result from sloppy complementary base-pairing leading to digestion by the DSN during normalization. To test this we first identified two distinct gene sets, the Ultra-Conserved Orthologs[65] (UCOs, http://compgenomics.ucdavis.edu/) and a subset of our EGPs, which we termed the Closely Related Genes (CRGs—the 300 most closely related EGPs, Ks <0.2). We then correlated the read counts from the normalized Illumina library and the average read counts from the combined non-normalized Illumina libraries and highlighted the UCOs and CRGs (S8 Fig). The results of this analysis do not support our hypothesis that the basis for aberrant removal of transcripts is due to high sequence similarity of CRGs since read counts for CRGs, compared to the UCOs and all other detected transcripts, are not substantially affected in the normalized library. Despite the aberrant removal of transcripts from sequencing libraries, the increase in average cDNA coverage (Fig 1) indicates that normalization was successful in its primary purpose.

**Fig 5:**
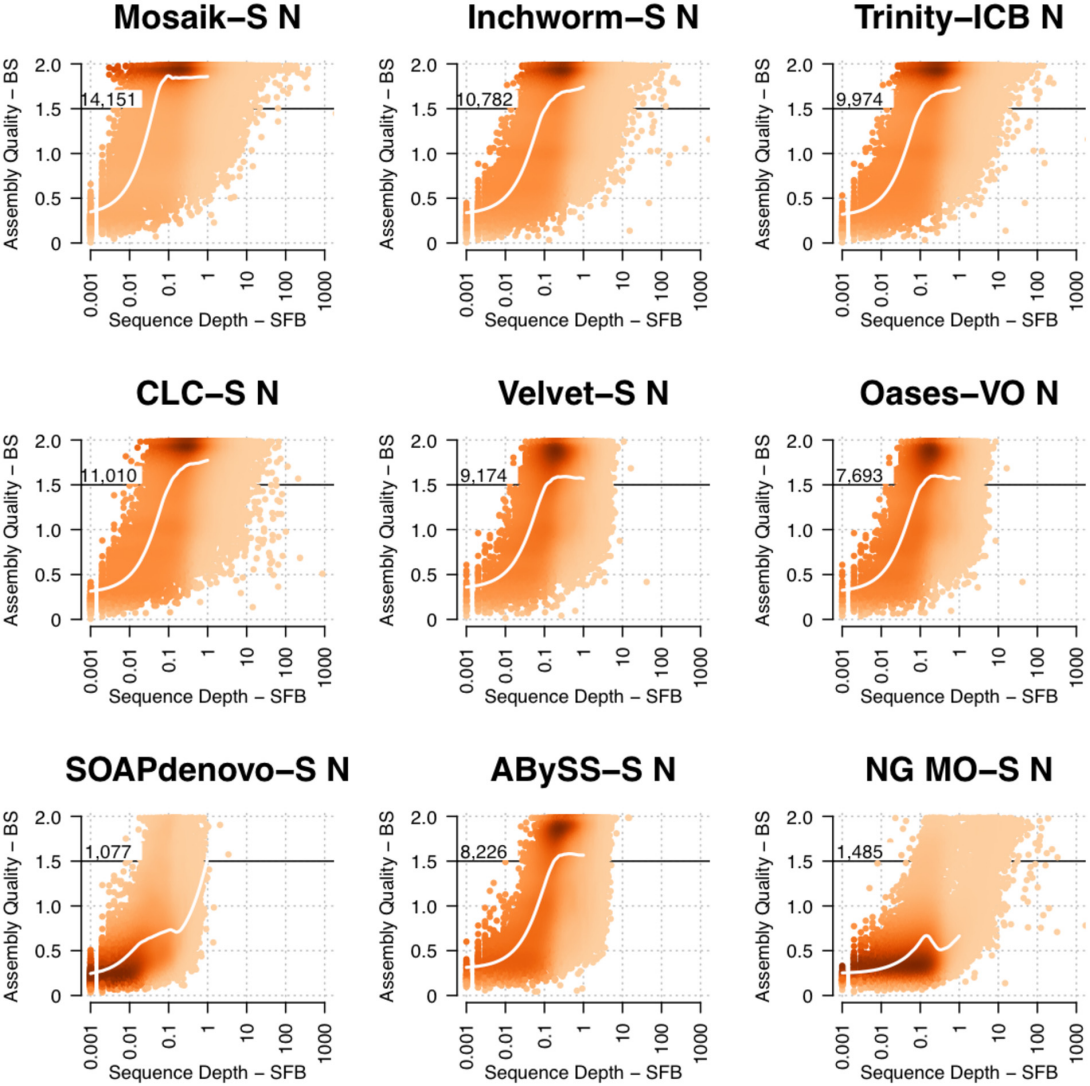
Normalization improves the recovery rate of highly expressed genes (compare to Fig 4). The units for “Assembly Quality” are Normalized Bit Score (BS) and the units for “Sequence Depth” are Sequenced Fragments/bp (SFB). The number printed in the plot area is the number of unigenes with normalized bit score above 1.5. A BS of 1.5 is an arbitrary threshold and is used to illustrate the differences in the high-density region seen in most plots near BS 1.75–2.

Since we verified that normalization was successful in increasing cDNA coverage (Fig 1 and S8 Fig), it stands to reason that *de novo* assembly of these data should improve since removal of excess reads directly addresses assembly failure at very high levels of expression (SFB >10, Fig 4). The assembly of normalized read data does increase the number of full length and accurate transcript assemblies (Figs 1 and 5, S8 Fig, S1 File) by ~15% in the Mosaik and Trinity assemblies, and by a larger margin in the CLC assembly (~25%). Similar gains were observed in Oases and ABySS assemblies, though NG MO and SOAPdenovo assemblies were notably poorer, due possibly to structural changes in the data following the removal of SMART adapter sequences (see Materials and Methods). SOAPdenovo was the only assembler considered here that *required* untrimmed input data, yet removal of the SMART adapters from was essential and could not be avoided. The structural data requirement of SOAPdenovo is a drawback since the user cannot tune the assembly based on quality trimming.

### Evaluation of *de novo* assembly without a genome reference

We have evaluated *de novo* assemblies leveraging the *Arabidopsis* genome as ground truth and provide the framework to evaluate assemblies and test the impact of various parameter adjustments. However appealing *de novo* assembly is for organisms with a reference genome, the power to study non-model organisms is undeniable. Using what we have learned about assembly quality with the *Arabidopsis* genome, we can now select the most informative reference-independent metrics for assessing assembly quality in organisms that lack a reference genome.

#### Read Titration Analysis

**Fig 6:**
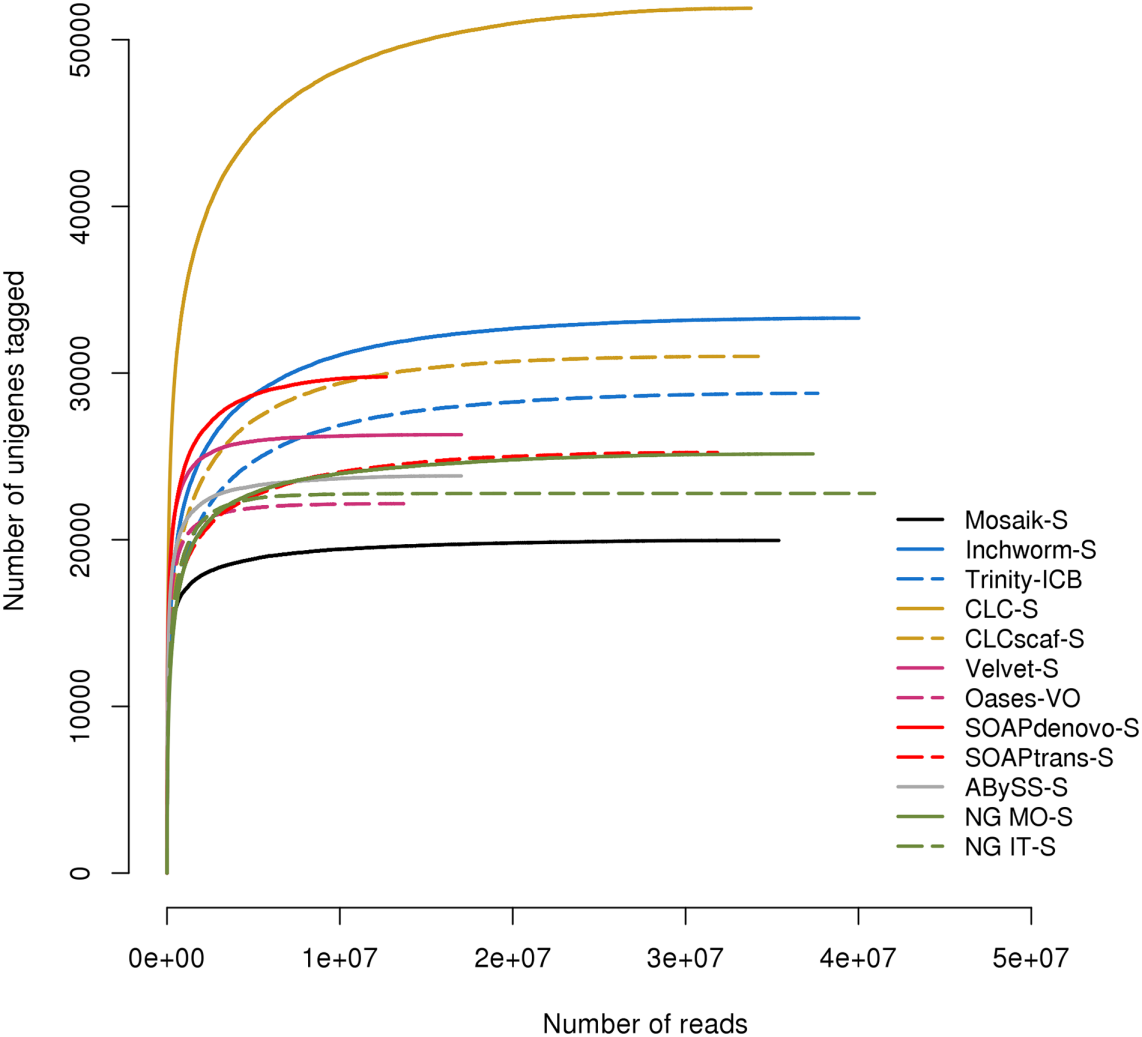
Read titration analysis. Reads were mapped to post-processed assemblies of biological replicate 1. The number of reads (X axis) that map to an assembly are an indicator of assembly completeness and quality, while the incidence of new tags (Y axis) indicates how completely the assembly reflects the diversity present in the read data.

In principle, if an assembly is accurate and complete (i.e., it represents all of the raw data), then a large proportion of those reads should map back to the assembly. In addition to the proportion of reads that map back to an assembly, a measure of completeness, in terms of number of genes detected, is necessary. To evaluate the degree of read saturation present in a *de novo* assembly, we calculated gene accumulation curves for each post-processed assembly of BR1 (Fig 6). This analysis calculates the rate of gene detection (estimated using the number of unique unigenes observed) as a function of sampling effort (number of sequence reads). Analogous to the species accumulation curves used to estimate species richness in biodiversity inventories, similar approaches have been used to evaluate gene capture in transcriptome studies for systems without a reference genome sequence[7, 34, 41, 46]. This approach allows us to qualitatively and quantitatively assess whether we have sequenced to sufficient depth to capture all of the genes present in a sample.

The read titration analysis shows that superior assemblies (like Trinity-ICB) are representative of the read data and capture a large amount of sample diversity before approaching saturation, at which point a large proportion of the data are sampled (Fig 6). It is clear that NG IT-S, NG MO-S, Inchworm-S, Trinity-ICB, CLCscaf-S and SOAPtrans-S reach saturation at ~110–150% of the expected gene count, while CLC-S has more than 3 times the expected number of sequences. We don’t expect a typical plant transcriptome to contain 60,000 genes, easily excluding CLC-S since the proliferation of unique sub-sequences causes an inflation of “unique tags” in the read titration analysis. Nor do we expect that only a small fraction of reads will map to a high quality *de novo* assembly, excluding Velvet-S, Oases-VO, ABySS-S and SOAPdenovo-S since they garner a very small proportion of reads before reaching saturation. However the plot of NG IT-S is quite similar to the Mosaik-S assembly, closer in fact than Inchworm-S, which we know to be a better assembly. This is a reflection of the highly accurate but inefficient and exclusive nature of NG IT-S assemblies. The unigenes that NG IT-S produces are very accurate and tend to require more reads for assembly while at the same time excluding conflict and many lowly expressed genes, effectively lowering the assembly diversity.

The relative sharpness of the inflection point informs assembly quality in that a more contiguous assembly will have a sharper inflection point (e.g., Mosaik-S). This is due to rapid detection of new unigenes in the assembly, that are quickly exhausted, and a switch to re-sampling high coverage unigenes. In assemblies that fail to reconstruct highly expressed genes, like Velvet-S, the inflection point is sharper due to the absence of highly expressed genes that lowers assembly unigene diversity. On the other end of the spectrum, CLC-S assemblies reconstruct highly expressed genes as a fragmented collection of unigenes resulting in the more gradual detection of unique, though highly expressed unigenes.

The results of this type of analysis will reveal which assemblies represent the read data well, while at the same time providing a measure of assembly diversity. In theory, a good assembly should produce a number of sequences that reflect the estimated transcript number. However, the choice between leading assemblers is not clear, as in the case of NG IT-S, NG MO-S, Trinity-ICB, Inchworm-S, CLCscaf-S and SOAPtrans-S. To make a more informed choice we should not only estimate the number of expected sequences, but also look for specific genes (e.g. broadly conserved genes) we expect to find.

#### Ultra Conserved Orthologs (UCOs) as a proxy for the whole transcriptome

**Fig 7:**
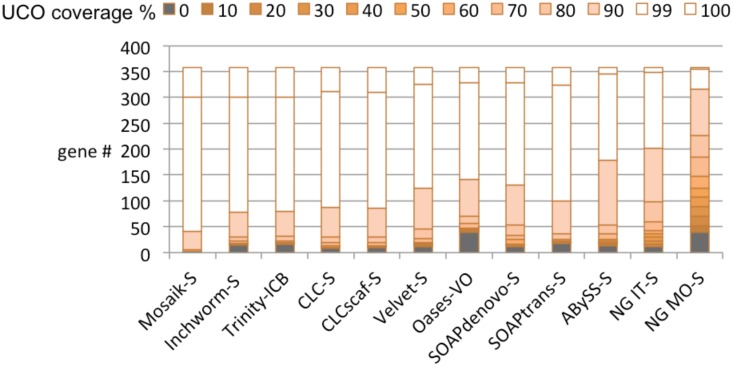
Ultra-conserved orthologs (UCO) coverage in post-processed assemblies of BR1. The use of UCOs as a proxy for the transcriptome assembly helps to identify leading assemblies when considered with the read titration curve analysis. When considered together, Trinity-ICB, which was the clear leader in our reference-based analyses, is also selected as the leader by the reference-*independent* metrics.

Our read level analyses suggest that UCOs are among the ~60% of genes expressed at moderate levels in the *A*. *thaliana* young leaf transcriptome (S8 Fig). The presence of UCOs in a *transcriptome* is an indicator of data completeness[41, 46]. Therefore, it follows that assembly of UCOs can be an indicator of *de novo* assembly quality since moderately expressed genes with low sequence similarity to other genes are likely to be assembled and represented by single high quality unigenes. The Mosaik-S assembly contained high quality unigenes (>BS1.5) for ~85% of UCOs and the leading *de novo* assemblers, Inchworm-S, Trinity-ICB. CLC-scaf and SOAPtrans-S all captured ~75% of UCOs with high quality unigenes (Fig 7). Combined with the read titration analysis we can now exclude NG IT-S and NG MO-S since the exclusive and inefficient nature of these assemblers results in an inferior complement of UCOs; NG IT-S lacks >100 UCOs covered at >90% and ~45 UCOs covered at >99% compared to the leading assemblies, while NG MO-S lacks even more. The results of these two genome reference-independent analyses clearly identify Inchworm-S, Trinity-ICB, CLCscaf-S and SOAPtrans-S as the superior assemblies, which is concordant with our ranking based upon the 10^th^ generation *A*. *thaliana* reference genome based analyses.

### A practical data volume threshold for *de novo* transcriptome assembly

**Fig 8:**
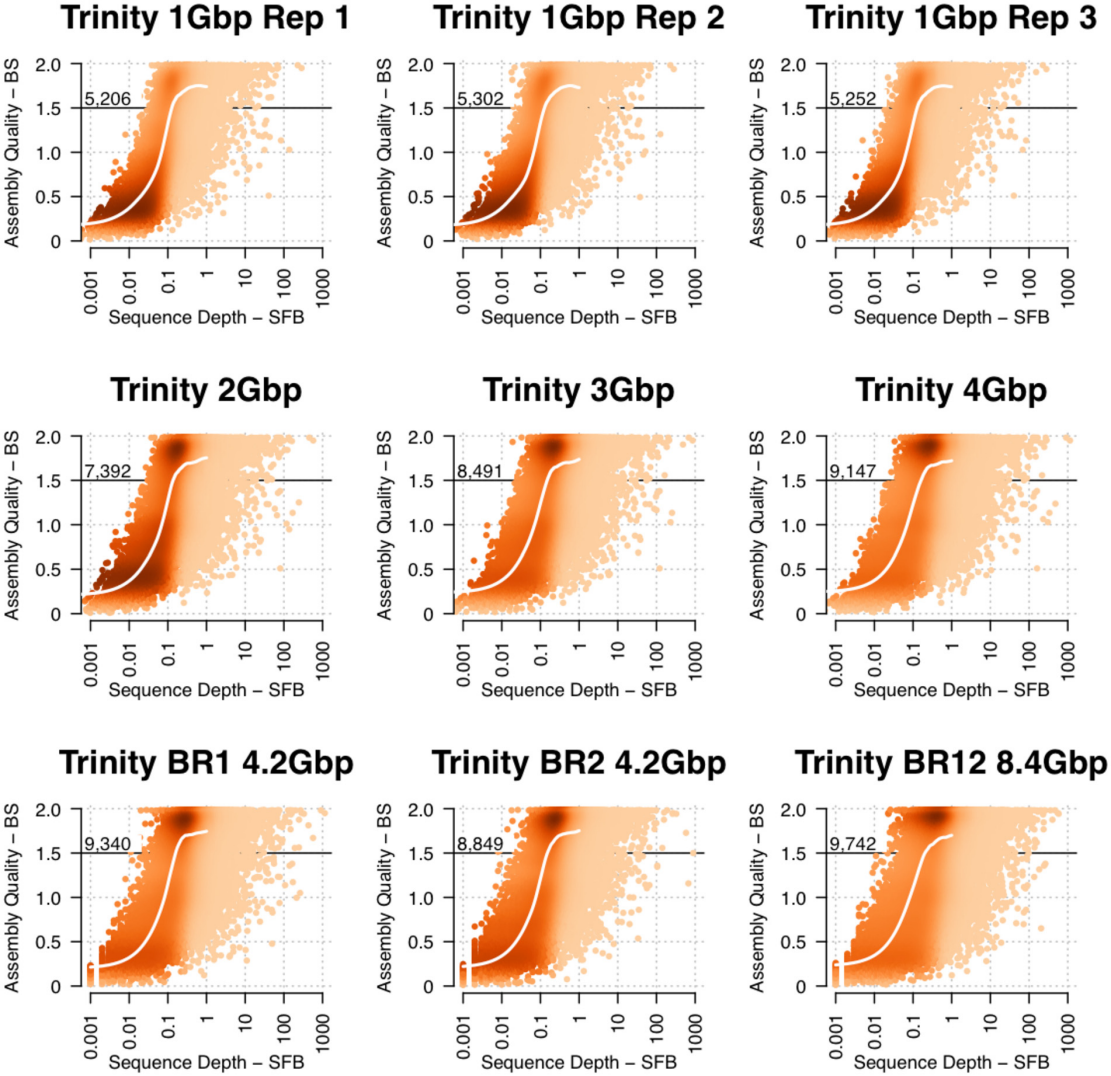
4 Gbp is a practical target volume for *de novo* transcriptome assembly. Illumina biological replicate 1 was subsampled to produce datasets of 1, 2, 3, and 4 Gbp. Replicate subsamples at 1 Gbp (top row) show reproducible results. Increasing the data by 1 Gbp to 2, 3, and 4 show diminishing increases in well assembled (>1.5BS) genes. The 4 Gbp subsampled assembly showed highly similar results to the biological replicate datasets. Doubling the data volume (BR12) produced a small increase in well assembled genes (>1.5BS) accompanied by a small increase in Type I mis-assembly. This analysis indicates 4 Gbp is a practical target data volume for *de novo* plant transcriptomes.

The *SCE*RNA processed Inchworm assembly of both biological replicates (BR12) of *A*. *thaliana* young leaf produced only marginally more (3.6%) unigenes above 1.5 BS than the assembly of one biological replicate (BR1), despite doubling the amount of sequencing data (from 4.2 to 8.4 Gbp). This indicates that for a majority of *A*. *thaliana* transcripts, 4.2 Gbp is near or above the threshold for successful assembly. Thus we subsampled BR1 to generate datasets of approximately 1, 2, 3 and 4 Gbp to explore the effect of sequencing depth on *de novo* transcriptome assembly using Trinity. Our subsampling method generated consistent replicate subsamples (n = 3) at ~1 Gbp that resulted in similar assembled transcript content (~95% of the detected transcripts shared) and a similar number of transcripts reconstructed at >1.5BS (5253±25 in each replicate—Fig 8).

The number of transcripts with >1.5 BS assembled from datasets of 1 Gbp, 2 Gbp, 3 Gbp, 4 Gbp, and 8 Gbp was 5253.3 (average of three replicates), 7392, 8491, and 9147, and 9742, respectively. The average number transcripts >1.5BS from BR1 and BR2 is 9,095, which is concordant with the 4 Gbp subsampled assembly. The number of unigenes >1.5 BS increased by 6.6% when we doubled the data volume from 4.2 Gbp to 8.4 Gbp. These gains are also accompanied by an increase in transcripts with *low* BS values, indicating an increase in Type 1 errors.

### Validation of *de novo* assembly in Rice

To evaluate leading assemblers and validate our assembly metrics in a more challenging plant transcriptome (larger and more complex[66–68]), we retrieved publically available (from ftp://ftp.ddbj.nig.ac.jp/) young leaf transcriptome data for *Oryza sativa* spp. Japonica cv. Nipponbare[69] that was structurally similar to our *A*. *thaliana* young leaf transcriptome data. A large proportion (76.5%) of the raw reads mapped back to the Rice cDNAs. Our subsampled assembly analyses in *Arabidopsis* predicted that the rice data set, which was 1.4 Gbp of quality filtered data, would be sufficient to reconstruct a substantial fraction of the rice young leaf transcriptome, allowing us to gauge the efficiency and accuracy of leading assemblers with select informative analyses.

Our rice assemblies showed patterns similar to the *Arabidopsis* assemblies (compare S9 Fig with Fig 8). Consistent with our subsampled assemblies in *Arabidopsis*, the small rice dataset was insufficient to reconstruct as much of the rice transcriptome as the full size (i.e. 4.2 Gbp) *Arabidopsis* transcriptome data. The difference in the number of genes above 1.5BS in the reference-based assembly vs. the best *de novo* assembly for rice was 4,186. The same comparison with the *Arabidopsis* transcriptome data revealed a smaller gap of 3,268 genes, but considering the smaller and less complex nature of the *Arabidopsis* transcriptome, this is not surprising. Importantly, the Type II error rates were low and only slightly higher (S3 Table) than those estimated in the assemblies of the *A*. *thaliana* young leaf transcriptome. Together, these analyses indicate that the superior assemblers for *Arabidopsis thaliana* young leaf transcriptome also perform well on the larger, more complex young leaf transcriptome of *Oryza sativa*.

### Microarray and RNA-Seq analysis of the *A*. *thaliana* young leaf transcriptome

We reasoned that we could improve the coefficient of gene expression between Microarray and RNA-Seq approaches by masking the RNA-Seq signal from all but microarray probe regions. We improved the Pearson’s correlation coefficient (PCC) between classic RNA-Seq (reads mapped to TAIR cDNAs) and the NimbleGen Multiplex Microarray from R^2^ = 0.817 (average Log_2_ RPKM vs. average Log_2_ Array intensity) to R^2^ = 0.853 (average Log_2_ reads/probe vs. average Log_2_ Array intensity). These results are an improvement over previous studies[64, 70] and consistent with Malone and Oliver[71], albeit with fewer RNA-Seq replicates. However, only ~2.5% of the Illumina reads map to the array probe sequences, excluding a majority of the read data.

The PCC between experiments using TAIR10 cDNAs and the Mosaik-S assembly revealed high similarity (R^2^ = 0.74) with the TAIR10 reference, which captures more reads (S10 Fig); this is expected because the TAIR reference is more complete. We correlated read counts from all *de novo* assemblies against a reference-based assembly of the same data (Table 3 and S1 File). The PCC between reads mapped to Inchworm-S vs. TAIR10 was good (R^2^ = 0.64), and the PCC between reads mapped to the Mosaik-S and Inchworm-S assemblies was better at R^2^ = 0.86 (S10 Fig).

Classic RNA-Seq and the NimbleGen Microarray reported very different expression levels for some genes. After verifying the probe sequences (checked against current annotations) and excluding organellar genes, we used the correlation analysis to choose candidates for qRT-PCR analysis. 12 genes (3 each in 4 categories of agreement and disagreement–S11 Fig) were chosen for analysis by qRT-PCR. Gene expression estimates for the well-correlated candidates were consistent across methods, especially within the dynamic range of the array (S12 Fig). Outside of the dynamic range of the array, qRT-PCR and RNA-Seq agreed (S12 Fig). Alternatively, the poorly correlated candidates were either cases of false positives on the array (2 of 3) or were erroneous signals from poorly annotated genes. For detailed results of the follow-up analyses on the 6 poorly correlated candidates, see S4 File.

### Transcriptome evidence for new *Arabidopsis* genes

The *A*. *thaliana de novo* assemblies include unigenes that do not align with annotated *A*. *thaliana* cDNA sequences. To determine the origin of these putative transcripts we used MEGAN[72] to classify these unigenes. A threshold alignment quality (bit score) of 125 was determined by plotting the frequency of hits to plants vs. the frequency of non-assignment (S13 Fig). At this threshold a substantial number of unigenes remain unassigned due to low quality alignments or low sequence complexity (S14 Fig). 357 unigenes from all post-processed assemblies of BR1 had best hits to fungal genes (S14 Fig) indicating low levels of contamination in the transcriptomes of the greenhouse-grown plants. The genus *Arabidopsis* was represented by 61 unigenes (collectively from all post-processed assemblies of BR1), which align to 20 unique sequences in NCBI’s non-redundant protein sequences database (see S5 File). Of these 20, none are represented by TAIR10 cDNAs. They are classified non-exclusively as follows: 14 align to the *A*. *thaliana* genome, 9 align to predicted (*ab intio*) *A*. *thaliana* mRNA transcripts, 15 align to *A*. *thaliana* ESTs (two of 15 are not present in the *A*. *thaliana* genome sequence), and one aligns to an *Arabidopsis lyrata* ribosomal protein (see S5 File). This collection of *Arabidopsis* unigenes represents evidence for new genes with concordant evidence in *A*. *thaliana* databases at NCBI[2]. If a more stringent alignment quality threshold is imposed, the remaining unigenes are classified primarily as fungi or into the genus *Arabidopsis* (S15 Fig) and the number of new genes detected falls by 50%.

## Discussion

### *De novo* assembler selection

The top performing assemblers were reassuringly good, and in many aspects competitive with the *A*. *thaliana* reference based Mosaik assemblies. Two of the top performers, Trinity[50] and SOAPdenovo-trans[30, 52], are open source, while the third, CLCscaf[51], must be purchased, yet it offers a more intuitive and user-friendly graphical user interface option combined with many other useful features. Although we did not examine computational speed in this study, no substantial differences were noticed between the top three performers (CLCscaf, Trinity and SOAPtrans) with our dataset. The run time and memory footprint of some of the assemblers evaluated here have been explored [73–74], and the computational intensity can vary greatly between data sets. A distinguishing feature of Trinity is the ability to estimate transcript isoforms. However, given the structural limitations of the data (read length and library fragment length << transcript length) the estimation is really of alternative splice *junctions* because short read data cannot capture complete transcript variants. Additionally, because expressed gene sets from different organisms can vary substantially in their gene duplication, isoform, heterozygosity, and repeat profiles, studies similar to this one in non-plants would have merit, especially as it relates to evaluating assemblies in non-model systems.

Our extensive list of assembly quality metrics falls into two categories 1) reference-dependent and 2) reference-independent. With a carefully controlled set of quality metrics, assembly parameters, and evaluation criteria we were able to identify superior assemblies in our reference-dependent evaluations. Once we identified the superior assemblies, we were able to choose reference-independent metrics that identified the same superior assemblies. Generally, when the assemblers were ranked based on a single metric, a subtle gradation in performance was revealed, rather than substantial differences. Using a wide range of assemblers, we observed the surprising result that all but the best *de novo* transcriptome assemblers (Trinity, SOAPtrans, CLCscaf) failed to assemble *highly expressed genes*. This observation indicates that both insufficient *and* excessive sequencing depth can cause assembly failure.

**Fig 9:**
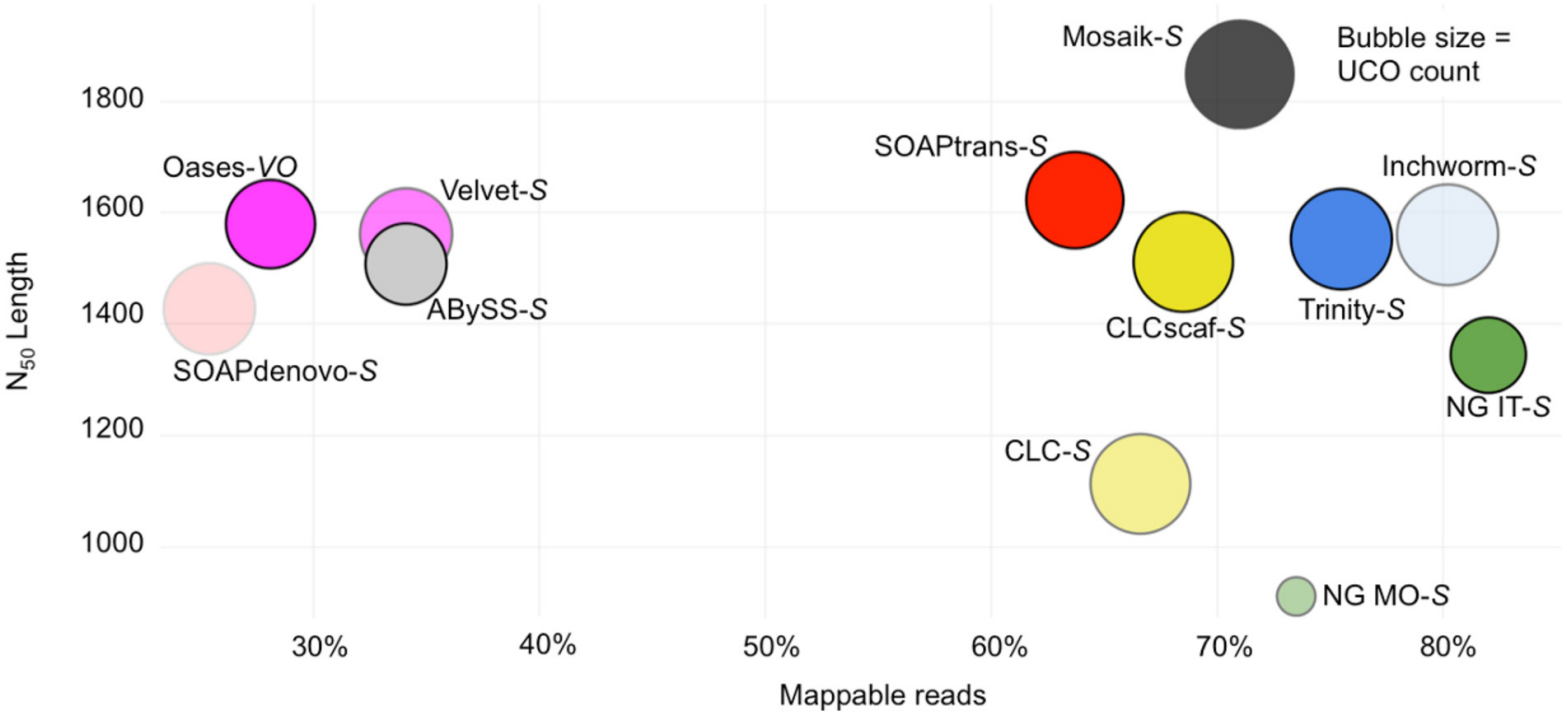
Integration of our reference-independent metrics shows the top 3 are closest to Mosaik-*S*. Considering the N_50_ length, proportion of mappable reads, and UCO recovery we recapitulate the reference-dependent ranking of *de novo* assemblers.

Examination of each reference based quality metric invariably led to questions about the useful combinations or relative power of each. To that end, we developed an analytical approach that reports unigene quality as a function of sequencing depth for each *Arabidopsis thaliana* transcript. Length statistics, which can inform unigene contiguity, are integrated with the frequency of all other errors (e.g. mismatches, gaps, insertions/deletions, and Type I and II errors). This method penalizes chimeric assemblies since the normalized score (BS) for an alignment to a reference cDNA will be low; only the matching portions will align leaving the remainder of the unigene unmatched. Finally, by considering the sequencing depth for each unigene, we can visualize the spectrum of sequencing depths and correlate that with highly accurate and contiguous unigenes to see how assembly quality changes over the dynamic range of gene expression. This key analysis (Fig 4) allowed a definitive ranking of assemblers that we recapitulated with reference-independent metrics (see Fig 9).

### Comments on the top 3 assemblers: CLCscaf-S, Trinity-ICB and SOAPtrans-S

Generally the statistics were fairly even among CLCscaf-S, Trinity-ICB and SOAPtrans-S assemblies of in terms of assembly size (~13.5 Mbp), N_50_ (~1550) and unigene count (~30,000). The differences among CLCscaf-S, Trinity-ICB and SOAPtrans-S were generally minor but indicate that SOAPtrans-S was more exclusive yet more accurate, and CLCscaf-S and Trinity-ICB more inclusive, possibly by more efficient resolution of variation in the transcriptome read data. One assembly does get an honorable mention along with a note of caution—NG IT-S. On the surface this assembly appeared to be very similar to our reference assembly controls with Mosaik. Yet after looking beyond the good N_50_ statistics, high proportion of mappable reads and other signs of a superior assembly we dug deeper to find that the assembly lacked many lowly expressed genes. It also lacked full-length unigenes (<99% cDNA coverage) for ~100 UCOs compared to the superior assemblies Together this indicates low read-use efficiency in NG IT-S and highlight the pitfalls of focusing on too few metrics of assembly quality. The substantial improvement over the NG MO-S assemblies resulted directly from manual removal of reads for highly expressed genes, supporting our conclusion that excessive sequencing depth poses a primary hurdle to *de novo* assembly.

Importantly, these leading assemblers displayed low Type II error rates. Our results show that previous estimates of error, specifically Type II error or instance of chimeric assemblies[30, 75], were artificially high, often by an order of magnitude or more. Part of this is due to the use of a loose definition of “chimera” in prior studies [29,70], but also to the use of a reference genome from a different rice cultivar that contributed to an inaccurate count of chimeric unigenes[30]. In the present study, we reserve the term chimera for clear cases of erroneous assembly of two distinct loci. We classified unigenes by aligning them to the *Arabidopsis* genome, and found that a majority of Type II errors were simply cases of alignment ambiguity (matches to multiple genome locations), where Xie et al.[30] broadly mis-classified these as chimeric. In fact they represent limitations of annotating closely related genes by aligning unigenes to a reference sequence, especially one from a *related* organism.

### Which genome reference-*independent* metrics identify the leading assemblers?

The reference-independent metrics that were most useful to identify superior assemblies were selected based on reference-based evaluations. Only the *combination* of 1) a high proportion of reads that map to an assembly 2) efficient recovery of conserved genes, 3) expected N_50_ length statistics, and 4) *more* unigenes than expected transcripts, is indicative of superior state-of-the-art assemblies (Fig 9).

Inconsistent with the expectation that superior assemblies produce the expected number of unigenes, superior assemblies produced *more* unigenes than the expected transcript number (~18,000). This is due to Type I assembly errors for very lowly *and* very highly expressed genes, both of which inflate the number of unigenes and depress N_50_ length statistics. Imposing length cutoffs to estimate N_50_ lengths reveals consistent and high values for leading assemblers. So, paradoxically, superior assemblies have more unigenes than transcripts since the large dynamic range of gene expression poses assembly challenges at *both* extremes. The reduction in Type I error rates by adding more data (Trinity-ICB assemblies of BR1 vs BR12) was small and accompanied by a concurrent increase in Type II error rates. This indicates that the 4.2 Gbp of BR1 was sufficient to reconstruct a majority of the transcriptome and approaches a point of diminishing returns as more data is collected.

An important reference-independent analysis was the read titration plot (see Fig 6). This analysis displays two important metrics that are useful to identify superior *de novo* assemblies: 1) proportion of mappable reads, and 2) unigene count. More subtle qualitative information is contained in the *shape* of the curve, with relative sharpness indicating relative assembly contiguity. The unigene number and proportion of mappable reads are easy to extract from other analyses, yet the read titration plot allows the researcher to examine *relative* transcriptome wide changes during iterations of assembly. For instance, the *relative* difference between two assemblies may be unigene contiguity, rather than unigene number or mappable reads. In the absence of excellent reference sequences to verify improved unigene contiguity, the read titration plot can reveal this improvement without a reference. It is important to note that absolute contiguity is more difficult to estimate without reference sequences, especially when assembly errors that reduce the effective transcriptome complexity can also indicate increased contiguity. Therefore, careful consideration of this integrated analysis with the other metrics is critical.

A strategy that has been implemented to evaluate *de novo* assembly is the detection of broadly expressed, highly conserved, single copy genes[7, 34, 41, 46]. A scan for genes expected to be present in the transcriptome (i.e. ultra conserved orthologs—UCOs or conserved single copy genes[58]) can inform assembly efficiency and completeness. In our comparison, a strong indicator of assembly quality was UCO coverage which, in leading assemblies, tagged >70% of the UCOs with unigenes covering >90% of the reference cDNA. As an exclusive indicator UCO coverage was not ideal since assemblies that fail at extreme transcript abundance may still assemble a large majority of UCOs since these genes tend to be moderately expressed (i.e. see the assembly Oases-VO BR1 in Figs 7 and 9).

We did not attempt to generate a single score by integrating the above 4 metrics. Reduction of these into a single dimension would require weighting of each metric. Because research goals vary, and the ability to leverage *de novo* transcriptome assembly may depend on differential weighting of these metrics, we instead have emphasized which aspects of assembly each metric informs to promote goal-specific assembly optimization.

### Are Strategies for Sequencing and Assembly Improvement Warranted?

We have shown that post-processing of assemblies is a critical step in generating superior assemblies. The Trinity and Velvet-Oases pipelines, and our collection of modular post-processing tools called *SCE*RNA, improved *de novo* assemblies in key categories while minimizing negative effects, such as increased Type II error rates (i.e. chimeric unigenes). *SCE*RNA had minimal effects on Mosaik assemblies indicating that high quality assemblies would not be compromised. The most dramatic improvement in our analysis was the implementation of the Iterative NextGENe assembly method[4] which brought NextGENe from a last place ranking to fifth place. This approach was labor intensive, but directly addressed assembly failure for highly expressed genes.

A common approach to improve the detection rate for lowly expressed transcripts is normalization. Our comparison revealed that the normalized Illumina library had increased average transcript coverage (Fig 1, S6 Fig and S3 File) and assembly quality (Fig 5 and S3 File) reflecting that abundant transcripts were reduced in frequency. However, we also observed that genes were erroneously removed during normalization. This, together with the cost of normalization, the loss of gene expression information, and the high value of replicated RNA-Seq data suggests the benefit of normalization is eclipsed by the benefits of deeper sampling and more biological replicates. If the sequencing goal is to discover the largest possible number of genes from an organism and obtain full length assemblies of even highly expressed genes[7], this is one time when the addition of whole-organism (i.e. mixture of multiple tissue samples) normalized libraries may provide a significant *addition* along with non-normalized libraries. This approach adds a transcriptome in which highly expressed transcripts are reduced in coverage, thereby increasing the chances for assembly of these transcripts.

It should be possible to avoid extremes of coverage by targeting specific tissues or “batch assembling” data to avoid assembly failures at extreme sequencing depths, yet this would require a novel meta-assembly strategy. Furthermore, in recent reports, dramatic gene expression differences were seen across fine tissue types (obtained by laser microdissection) in the developing maize leaf[76], tomato fruit[9], and even parasitic plant haustoria[5]. These global differences in gene expression likely extend to gene expression patterns of duplicated genes and UCOs. Even though recently diverged genes (i.e. lower Ks) tend to be co-expressed while older duplicate genes (greater Ks) have evolved divergent expression patterns[58–62], these dramatic cell type differences may indicate a challenge to these observations. Also while UCOs tend to be broadly and moderately expressed in whole organ samples like our young leaf samples, increased sampling granularity may influence the recovery of UCOs if their expression profile is highly cell-specific.

Thus, in the absence of complementary data types, it may be possible to improve assembly by isolating difficult-to-assemble transcripts by fine sampling. Similar to the addition of normalized libraries to decrease reads for highly expressed genes, high sample granularity increases the number transcripts with data amounts in the optimum range (SFB ~0.1–1.0) for successful assembly. The loss of low abundance transcripts from a fine granularity approach would be mitigated by assembly of all the data together; an approach that effectively increases the coverage of low abundance transcripts. However, implementation of a multiple assembly strategy creates the need for a meta-assembly approach to merge or cluster unigenes from the same locus, or a selection strategy to supplement the easily assembled transcripts with those that are more challenging to assemble.

From strictly a cost-per-base perspective, Illumina sequencing is attractive. However, longer read technologies (e.g. Sanger, 454, PacBio, Oxford Nanopore) are attractive since they provide complementary data structure to capture entire transcripts. Recent reports have shown that excellent *de novo* assemblies can be generated by leveraging existing sequence resources with fresh SGS data, like those that exist for wheat[77] and tomato[9]. However, we recognize many plant genomes are first examined by a pilot Illumina transcriptome (e.g. NSF’s AToL initiative[78] and the 1000 Plants initiative[79]), thus researchers must rely solely on the SGS data created in these studies.

Important situations where long reads should improve the assembly quality are in the assembly of highly expressed genes and closely related genes (CRGs). We examined CLC assemblies of a combination of 454 and Illumina (S1 File) to see if structurally different data improved assembly. Compared to a CLC assembly of Illumina-only, assembly metrics were nominally improved; a similar improvement was achieved by implementing the overlap layout assembler CAP3 with only Illumina data. Importantly, our hybrid assemblies were inferior to the leading Illumina-only assemblies, which include CLCscaf-S. Surprisingly, the *de novo* assemblies generally outperformed the *Arabidopsis* genome based assemblies (using Mosaik) by reconstructing a greater proportion of high quality (>1.5 BS) mates of CRGs.

The global pattern of CRG pair expression was examined by Ks plot analysis (S3 Fig) revealing that most CRGs detected by read mapping (S3 Fig “Detected gene set”) were not sufficiently expressed for assembly of both mates (S5 Fig). In the *de novo* assembly plots the abundance of pairs with very low Ks (the negative exponential on the left side of the plot) likely results from very similar subsequences or transcript isoforms. The plot of ABySS Multi-K-S (S3 Fig), the largest and consequently the most redundant assembly, is an extreme example of this. The post-processing tools in the Velvet-Oases and Trinity pipelines outperform *SCE*RNA in this regard, yet the assembly that was most improved by *SCE*RNA (CLC) shows a plot which is nearly identical to the reference based assembly. Since *SCE*RNA was optimized for improving CLC assemblies, which were the most complete but most fragmented assemblies, this result suggests that post-processing may benefit from platform specific tuning. The Ks analysis is useful as an indicator of relative assembly quality, but the variable detection rate of differentially expressed genes imposes limitations for using the Ks plot as an indicator of absolute quality. Indeed, a meta-assembly strategy where sufficiently diverse transcriptomes are sampled to capture virtually all transcripts may enhance the utility of this type of analysis.

### Assembly validation in *Oryza sativa*

*Arabidopsis thaliana* is an ideal system for our in depth *de novo* transcriptome assembly evaluation. However, in the universe of plant transcriptomes it poses fewer challenges than some since the *Arabidopsis* transcriptome is relatively small and low complexity. Notably, the Type II error rates were only slightly higher in the rice assemblies compared to the *Arabidopsis* assemblies. Curiously the genome reference-based Mosaik returned ~5x more unigenes with Type II errors. This may be due to the inability to align reads unambiguously to closely related genes or may result from the relatively poorer 7^th^ generation rice genome annotation compared to the 10^th^ generation *Arabidopsis thaliana* genome. Overall, these select analyses in rice show that leading assemblers have similar efficiency and fidelity when used for the more complex rice transcriptome.

### *De novo* RNA-Seq

RNA-Seq has emerged as a powerful tool for gene expression analysis, yet the availability of a high quality read mapping reference is essential. We have shown that by masking the signal for RNA-Seq to only the cDNA probe binding regions the two methods of expression analysis yield strongly comparable results providing a stronger link to legacy gene expression data. Additionally, we have shown that high quality *de novo* assemblies can serve as an effective reference for RNA-Seq. For *de novo* RNA-Seq, Trinity offers a balance of completeness, contiguity, and accuracy with the exclusive feature of inferring cluster variants (i.e. transcript isoforms).

Our follow-up analysis of the 12 candidates (S11 and S12 Figs and S4 File) revealed that in each case when microarray and RNA-seq values disagreed, the RNA-Seq values were concordant with qPCR, indicating microarray errors. This analysis reveals that a potentially useful way to test the accuracy of genome annotations, as well as the probe set on an array, is to correlate the analog array probe signal with the digital signal from RNA-Seq masked for all but the probe sequences. Even though our sample size was small, we were able to identify problematic probe sequences and identify potential annotation errors in the best plant genome.

### Conclusion

*de novo* assembly of the *Arabidopsis thaliana* young leaf transcriptome has shown that, compared to a reference guided assembly, reconstruction of the transcriptome is reassuringly good. The leading assemblies were Trinity-ICB (and the highly similar Inchworm-S), CLCscaf-S and SOAPtrans-S and generally the unigenes produced by each were highly accurate while assembly errors were largely restricted to fragmentation and non-assembly. The reference based transcriptome assembly metrics revealed that a large dynamic range of expression poses assembly challenges at both extremes. Superior assemblies accurately reconstruct transcripts efficiently at low transcript coverage depth and continue to generate accurate assemblies at high coverage depth. Paradoxically, the superior state-of-the-art assemblies routinely produced more unigenes than expected transcripts due to assembly failure at both extremes of expression. Type I error rates, which are due to insufficient coverage of lowly expressed genes and, presumably, to conflict and error in extreme coverage depths, are a dominant form of assembly error. The rates of all other types of error, including chimeras, were low. The reference-independent metrics we examined, when considered together, can inform absolute assembly quality. Existing prior knowledge about the characteristics of a given transcriptome, e.g. gene number, are important to assessing assembly quality and each plant transcriptome has unique differences. Also, highly tissue specific sampling will influence the transcriptome that is captured compared to a tissue pool that consists of more cell types. The characteristics of a superior transcriptome assembly include: 1) a high proportion of mappable reads (e.g. >65%), 2) unigenes that number roughly ≥150% of the expected transcript count, 3) expected N_50_ (e.g. 1200), and 4) high recovery of Ultra Conserved Orthologs. Only this *combination* of reference-independent metrics was useful to identify superior assemblies.

The minimum coverage required for successful assembly is notoriously hard to estimate since transcript abundance spans several orders of magnitude. Indeed, a minimum depth for assembly of highly expressed genes is easy to reach in a very modest (i.e. 500 Mbp) dataset while the required depth of coverage for successful assembly of lowly expressed genes may be practically unreachable. Our analyses suggest that 4–5 Gbp is a good minimum target for a representative plant transcriptome, since substantial gains are made at 1 Gbp increments from 1–4 Gbp, but beyond that (i.e. ~8 Gbp in BR12) the returns diminish in terms of yield of additional transcripts, though more data may be required for larger transcriptomes, as seen in rice. It is very important to note that more data can simultaneously improve the assembly of lowly expressed genes while increasing the frequency of mis-assembly of highly expressed genes. The intractiblity of sequencing each transcript in the sweet spot may require custom digital normalization schemes combined with meta-assembly of tissue specific data, or “batch assembly” of transcriptome data. Such an analytical strategy, in combination with effective use of 3^rd^ generation sequencing technologies, could address the remaining weaknesses of state-of-the art *de novo* assemblers in a world with exponentially growing transcriptome data.

## Materials and Methods

### *Arabidopsis* growth conditions

*Arabidopsis thaliana* Col-0 was grown in the Penn State Biology department greenhouse (http://www.bio.psu.edu/general/greenhouse). Seeds were not stratified and sown in a lawn at a density of approximately two seeds/cm^2^. The plants were grown for 21–28 days in Metro-mix 360^™^ (SunGro) in late September through early October. Hundreds of young leaves ranging from 7–12 mm in length were harvested at the petiole-blade junction with fine forceps and flash frozen in liquid N_2_. Tissue from two biological replicates (each sampled from four trays), grown tandem under similar conditions, was harvested and then stored at -80°C.

### Isolation of total RNA

Frozen leaf tissues were macerated in the presence of liquid N_2_ in an RNase free, pre-chilled mortar and pestle. Tissue was processed in ~200 mg portions using the RNAqueous^™^ Midi large scale phenol-free total RNA isolation kit (Ambion) according to the manufacturer’s instructions with the following exceptions: 1) lysis buffer solution was made fresh for each isolation, 2) Plant RNA Isolation Aid (Ambion) was added to each lysis buffer prep in a 1:8 ratio by volume. Total RNA was assessed on the Agilent Bioanalyzer using the RNA 6000 Nano kit (Agilent) with the Plant Total RNA assay. High quality total RNA samples (28s/18s ratio ≥1.7; RIN ≥8; A_260_/A_280_: ≥1.8) from individual biological replicates were pooled. Biological replicates were never mixed except prior to normalized library construction.

### RNA precipitation and concentration

To further purify and concentrate RNA samples they were divided into ~100 μg portions and were precipitated by adding 0.1 volumes RNase-free 3M NaOAc pH 5.2, and three volumes 100% reagent grade Ethanol and incubated at -80°C overnight. Precipitated total RNA was collected by centrifugation at 14000 x G at 4°C for one hour and the supernatant was discarded. The resulting pellet was washed twice with ice-cold 100% ethanol with a five minutes spin at 14000 x G following each wash and the supernatant was discarded each time. The pellet was allowed to air dry for 60 seconds and was resuspended in 100 μL RNase free water. Multiple precipitated samples from each biological replicate were pooled by replicate and mixed thoroughly before being stored at -80°C.

### DNase treatment

Total RNA was treated with 2 U amplification grade DNase (Invitrogen) in 100 μg aliquots in a total volume of 100 μL with a final buffer concentration of 1x and 40 U of RNase OUT^™^ (Invitrogen) at 25°C for 30 min. RNA was isolated from the reaction using an RNeasy^™^ (Qiagen) mini kit following the manufacturer’s instructions, with the following exceptions: 1) 350 μL of buffer RLT was added directly to the DNase reaction, 2) RNA elution was performed in two steps using 30 μL of RNase free water each time. Total RNA was re-assessed on the Agilent Bioanalyzer using the RNA 6000 Nano kit (Agilent) with the Plant Total RNA assay following DNase treatment.

### Paired-end mRNA-Seq library construction

*Arabidopsis thaliana* total RNA was assessed on an Agilent Bioanalyzer using the RNA 6000 Nano Kit (Agilent) with the Plant Total RNA Nano assay. Only high quality (28s/18s ratio:≥1.7; RIN ≥8; A_260_ /A_280_: ≥1.8) RNA was used to prepare Illumina Paired End mRNA libraries according to the mRNA-Seq Sample Prep Guide (Illumina, 1004898 Rev. D) following the manufacturer’s instructions. The Illumina RNA-Seq libraries were assessed on the Agilent Bioanalyzer using the DNA 1000 kit (Agilent) with the DNA 1000 assay.

### Paired-end sequencing on the Illumina Genome Analyzer IIx

Sequencing of the Illumina PE mRNA-Seq libraries was done in the McCombie lab at the Cold Spring Harbor Laboratory, Cold Spring Harbor, NY USA. Each non-normalized library was sequenced in one lane following a paired-end (76x76 bp) sequencing cycle protocol. Data are publicly available with accession number SRP065775.

### Normalized Illumina paired-end mRNA-Seq library construction

20ug of high quality (28s/18s ratio: ≥1.7; RIN ≥8; A_260_/A_280_: ≥1.8), DNase treated RNA from each biological replicate was thoroughly mixed. Pooled samples were provided to Chris Pires at the University of Missouri, Columbia for cDNA synthesis (Evrogen Mint dscDNA synthesis kit), normalization (Evrogen Trimmer Kit cDNA normalization kit) and sequencing on a single lane following a paired-end (120x120 bp) sequencing cycle protocol and is publicly available (accession number SRP065775).

### Poly-A RNA enrichment for 454 sequencing

RNA samples were enriched for Poly-A RNA using the Poly(A) Purist^™^ mRNA purification kit (Ambion) according to the manufacturer’s instructions. Poly-A enriched RNA was assessed on the Agilent Bioanalyzer using the RNA 6000 Nano kit (Agilent) with the mRNA assay.

### cDNA synthesis for 454 sequencing

A separate cDNA synthesis step was used for libraries prepared for 454 sequencing and for rtPCR. cDNA was synthesized using the JGI cDNA synthesis protocol version 1.0 (http://my.jgi.doe.gov/general/index.html) with the following modifications: 1) multiple reactions were pooled for phenol:chloroform extractions, 2) an additional chloroform extraction following the phenol:chloroform extraction at step 3 was done, 3) Roche 454 GS20 library preparation specific steps (4–7) were omitted. cDNA was evaluated on a Agilent Bioanalyzer using the DNA 7500 kit (Agilent) with the DNA 7500 assay. The expected, and observed, yield by mass of cDNA from poly-A selected RNA was 50–75%.

### Roche 454 FLX cDNA library construction

High-quality cDNA showing a mean length of ~1500 bp or greater was used to prepare 454 FLX libraries according to the Roche 454 GS DNA Library Preparation Kit (Roche, 04852265001) following the manufacturer’s instructions. The 454 GS FLX libraries were assessed on the Agilent Bioanalyzer using the DNA 7500 kit (Agilent) with the DNA 7500 assay.

### Roche 454 FLX sequencing

Sequencing of the Roche 454 FLX cDNA libraries was done at the Penn State Genomics Core Facility—University Park, PA USA. Each library was sequenced on ¼ plate.

### Eurofins MWG Operon 454 GS-FLX Titanium normalized transcriptome

20ug of high quality (28s/18s ratio: ≥1.7; RIN ≥8; A_260_/A_280_: ≥1.8), DNase treated RNA from each biological replicate was thoroughly mixed and provided to Eurofins MWG Operon. RNA quality was verified post shipment with the Shimadzu MultiNA microchip electrophoresis system (Shimadzu). Poly-A RNA was prepared from the total RNA. First-strand cDNA synthesis was primed with random hexamers. 454 adapters A and B were ligated to the 5' and 3' ends of the double stranded cDNA (ds-cDNA). cDNA was amplified with PCR (16 cycles) using a proof-reading enzyme. Normalization was carried out by one cycle of denaturation and reassociation of the cDNA. Reassociated ds-cDNA was separated from the remaining (normalized) single-stranded cDNA (ss-cDNA) by passing the mixture over a hydroxylapatite column. After hydroxylapatite chromatography, the ss-cDNA was amplified with 10 PCR cycles. Adapter ligated ds-cDNA fragments were resolved on a preparative agarose gel. Fragments ranging from 500–700 bp were excised and an aliquot of the size fractionated (~500–700 bp) cDNA was analyzed by capillary electrophoresis with the Shimadzu MultiNA microchip electrophoresis system (Shimadzu). The library was sequenced on ½ plate.

### Micro Array hybridization and analysis

*Arabidopsis thaliana* total RNA was provided to the Penn State Genomics Core Facility—University Park, PA. Total RNA samples were amplified and labeled for 2-color hybridization and the labeled *A*. *thaliana* aRNA was hybridized to the *Arabidopsis thaliana* 4x72K Array (A4511001-00-01) (protocol: http://www.huck.psu.edu/facilities/genomics-core-up/faq/nimblegen-microarrays-procedure). Arrays were scanned using the MS 200 Microarray Scanner (NimbleGen) and the MS 200 Data Collection Software (NimbleGen) according to the NimbleGen Array User’s Guide (V3.2, NimbleGen). Background correction of the array images was done using NimbleScan (NimbleGen) and RMA normalization was done on the raw signal intensities from technical replicates using NimbleScan (NimbleGen). Log_2_ transformed, background-corrected and normalized signal intensities were used as estimates of gene expression in subsequent comparisons.

### qRT-PCR candidate gene selection

Gene candidates were chosen based on a correlation of the NimbleGen *Arabidopsis thaliana* 4x72K Array intensities and the average PE Illumina mRNA-Seq read numbers mapped at high stringency to NimbleGen array probe sequences (no mismatches and alignment with the full probe 60mer). Well correlated examples (n = 3) that spanned the dynamic range of log transformed reads counts from Illumina were chosen to corroborate the estimates of expression reported by the array and RNA-Seq. Features that extend beyond the range of the array (n = 3) that should follow the well-correlated trend were chosen as the other 3 “well correlated” candidates. Poorly correlated candidates (n = 6, with n = 3 high in array, low in RNA-Seq and n = 3 vise versa) were also chosen for interrogation by qPCR. Candidates were selected by choosing the most poorly correlated estimates of gene expression that met the following criteria: 1) gene models were not obsolete in subsequent iterations of the *Arabidopsis* genome (then current TAIR9), 2) complete probe set must match perfectly to the then current TAIR9 gene model, 3) gene must be nuclear, protein-coding transcript, 4) candidate must fall within log2 array intensity of 4 to13, and 5) the then current TAIR9 annotation did not include known splice variants.

### qRT-PCR

We searched for candidates in each category (S11 Fig) that had verified (still accurate from TAIR6 to TAIR9) probe sequences. The 6 candidates for the well-correlated group (black arrows S11 Fig) were easily chosen and were the first 6 examined. Alternatively, the search for candidates in the poorly correlated group (red arrows S11 Fig) extended to ~30 candidates before any were chosen due to updated or obsolete annotations, and each of these had only 2 probes compared to 3 for the well-correlated gene set. *Arabidopsis thaliana* total RNA was provided to the **Penn State Genomics Core Facility—University Park, PA.** Primers and probes for the 12 candidates were designed using Primer Express (v2.0, Applied Biosystems). For qRT-PCR analysis high quality, DNase treated RNA was reverse-transcribed with the High Capacity cDNA Reverse Transcription kit (Applied Biosystems) following the manufacturer’s instructions. Relative quantification by real-time PCR was determined by adding 10 or 20 ng of cDNA to 2X TaqMan Universal PCR Master Mix (Applied Biosystems) in a volume of 20 μLs. Primers were added at a concentration of 400nM and the TaqMan probe, labeled with a 5' FAM and a 3' Black Hole Quencher (Biosearch Tech, Novato, CA), at 200nM. The amplification protocol consisted of 10 min at 95°C, followed by 40 cycles of 15 sec at 95°C and one min at 60°C in the 7300 Real-Time PCR System (Foster City CA).

### qPCR data analysis

Average crossing point (Ct) values for each gene and PCR efficiency, calculated by E = 10^(-1/slope)^, [80] was used in the E^ct^/E^ct^ calculation [81] to determine the transcript abundance relative to the reference gene AtActin (AT3G18780) in both biological replicates. We made two key assumptions for this analysis, 1) the cDNA population accurately represents the poly-A RNA population from which it was made (standard assumptions for micro-array and RNA-Seq) and 2) for the TaqMan assay the number of amplicons at the crossing point is the same for each reaction.

### Informatics

#### Sequencing

The *Arabidopsis thaliana* young leaf transcriptome normalized and non-normalized libraries were sequenced with both Roche’s 454 GS-FLX and Illumina’s GAIIx. 454 sequence fragments were clipped with version 0.2.8 of sff_extract[82] at the recommended clipping points by the 454 software. Low-quality bases (<Q20) were trimmed from the ends of 454 single-end and Illumina paired-end reads using the *quality_trim* program of CLC Assembly Cell version 3.2[51] requiring additionally that the remaining read must be >50% original length with each remaining base having >Q20. SnoWhite[83] (version 1.1.4), a sequence-cleaning pipeline, was used to remove normalization adapter (sub-)sequences from the normalized read files. SnoWhite was originally implemented for 454 data, and requires sequences in fasta format (and optionally base qualities in base-quality fasta format). Since Illumina sequences are typically provided in fastq format, Biopython[84] was used to convert the Illumina-fastq to standard-fastq, and then the resultant standard-fastq to fasta and base-quality fasta format before clipping adapters. After running Snowhite the read files were converted back to fastq format using Biopython, paired-end read files were reconstructed and single-end read files were written (for orphaned reads) using a custom script.

#### Reference Read Mapping

A reference-based assembly was created for detailed comparison with the *de novo* assemblies (below). All reads were aligned to TAIR10[85] cDNA representative gene models (sequences for the longest CDS at each locus) using version 1.1.0014 of Mosaik Assembler (http://bioinformatics.bc.edu/marthlab/Mosaik) with the recommended aligner settings. Mosaik alignments were converted to bam format and *CoverageBed* program in Bedtools[86] was used to compute the depth and breadth of coverage, and to determine the number of TAIR10 gene sequences tagged. 25,512 TAIR10 gene models were tagged by at least one read in any of the data sets and are here after referred to as the “detected gene set” in *Arabidopsis thaliana* young leaf.

#### Transcriptome assembly

The trimming of low-quality bases and adapter clipping can compound insert size estimation by read mapping. To estimate insert sizes required for subsequent analysis steps, test assemblies were run for all three Illumina paired-end reads libraries BR1, BR2 and NORM using CLC Assembly Cell (Version 3.2). Then all paired reads were uniquely mapped to each lllumina library and its respective large contigs (≥600 bp) using version 0.12.7 of Bowtie[87]. This enabled estimation of the average insert size for each library using custom PERL script “get_insert_sizes_from_bowtie_aln.pl” (see S6 File). The insert sizes for biological replicate one (BR1), biological replicate two (BR2), and normalized (NORM) libraries were 134 bases (sd = 15.06), 140 bases (sd = 18.15), and 217 bases (sd = 19.45) respectively.

#### Reference assembly

Mosaik Assembler was used to resolve paired-end read alignments, filter out duplicate alignments, and assemble reference sequences from previously created Mosaik reference read alignments. Reference scaffolds were created from all Mosaik reference assembly ACE files for each assembly by clipping flanking reference bases of the consensus sequence, substituting intervening reference bases of the consensus sequence with nucleotide ambiguity code N, and filtering out sequences shorter 100 bases (not including scaffolding Ns). Reference based primary assembly statistics are shown in S1 File.

#### *De novo* assembly

The following *de novo* assembly tools were used and evaluated in this study: ABySS[55] (version 1.3.0), CLC Assembly Cell[51] (version 3.2) and CLC Assembly Cell with Scaffolding (version 4.0.6 beta), Oases[18] (version 0.1.22) with Velvet[53] (version 1.1.03), SOAPdenovo[52] (version 1.04) and SOAPdenovo-trans[30] (version1.01), Trinity[50] (release 03122011, includes Inchworm; release 04132014 for subsampled assemblies of *Arabidopsis* and assemblies of *Oryza*), and NextGENe[88] (version 2.17). *de novo* assemblies (see S2 Table) were performed with default settings whenever possible except for the minimum contig/scaffold cutoff of 100 bases, *k-value* of 31 (except for the Trinity pipeline which is only compatible with 25-mers), and scaffolding turned on whenever applicable. ABySS, Oases (Velvet), SOAPdenovo, SOAPdenovo-trans and CLC Assembly Cell + Scaffolding produced scaffolds, while CLC Assembly Cell and Trinity (Inchworm) produced contigs. All assemblies were performed using trimmed reads except for SOAPdenovo non-normalized assemblies, which became more fragmented as result of trimming. Trimmed normalized reads were used in SOAPdenovo assembly because the untrimmed normalized reads were contaminated with adapter (sub-)sequences. To investigate the effect of a mixture of 454 and Illumina reads a 454-Illumina-hybrid assembly was performed using reads from BR1 with CLC Assembly Cell. For the NextGENe Maximum Overlap (NG MO) assemblies we used NextGENe to quality filter the raw fastq data to remove reads with a median quality score of less than 22, trim reads at positions that had 3 consecutive bases with a quality score of less than 20, and remove any trimmed reads with a total length less than 40 bp. The quality-filtered data was assembled *de novo* using the Maximum Overlap assembler in NextGENe. The NextGENe Interative assemblies (NG IT) were done as described in Wickett et al. 2011[4]. We attempted to improve the BR1 hybrid 454 + Illumina CLC assembly using the overlap consensus assembler CAP3[89], using a minimum overlap length of 30 (*k-value* -1) and at least 97% identity, to merge contigs with significant overlap that had not been assembled into contiguous sequences (due either to single base mismatches in the reads or path ambiguity in the graph). Illumina BR1 reads were also assembled over a range of *k*-*values* (25–74) using ABySS (ABySS Multi-K) and these assemblies were merged to remove redundancy as described in Robertson G, Schein J, et al. 2010[90].

#### Subsampled assemblies

We generated 3 replicate subsamples of 1 Gbp using the SubSampleFastq program (https://github.com/dylanstorey/SubSampleFastq) to test if randomly subsampled sets of the same size would provide consistent assembly metrics. After validating the subsampling scheme, we randomly generated subsamples of approximately 2 Gbp, 3 Gbp and 4 Gbp of paired-end fastq reads from BR1 to explore the effect of sequencing depth on de novo transcriptome assembly.

#### Assembly post-processing

We developed a suite of post-processing tools we call *SCE*RNA (Scaffolding Clustering Error correction of RNA-Seq data–S6 File). These assembly post-processing tools address specific aspects of an assembly with the goal of generating high confidence sequences for downstream analysis. *de novo* assemblies had different entry points into the post-processing pipeline depending upon the output of each assembler (Fig 2). Paired-end information of Illumina reads was used to scaffold and extend CLC Assembly Cell and NextGENe contigs with SSPACE[91] (version 1.0) because assembly algorithms of these two assemblers do not have built-in scaffolding functions and thus tend to produce more fragmented sequences (CLC now has a built in Scaffolding feature—designated herein CLCscaf see S2 Table). A minimum of 5 links (read pairs) were required to join two contigs into a scaffold and at least 30 bases of overlap (*k-value* -1) and a minimum of 20 reads were required for contig extension. Similar parameters were set for the assembly programs with built-in scaffolding capabilities, whenever possible. In order to bridge some of the gaps introduced in transcripts during scaffolding, paired-end reads were utilized with SOAPdenovo’s *GapClose*r program (overlap length set at 30 bases (*k-value* -1)) to either close or reduce the size of gap opening in all *de novo* assemblies except for the gapless transcripts produced by Trinity. ESTScan 2.0[92] using HMM models built with *A*. *thaliana* was used to identify translatable sequences. USEARCH[93] (version 4.0) was used to de-replicate (create non-redundant) *de novo* assemblies before the terminal clustering steps of *SCE*RNA. Using UCLUST, a global alignment-clustering algorithm of USEARCH, scaffolds (or contigs where applicable) were clustered at 97% identity for each *de novo* assembly into non-redundant sets. The longest sequence in each cluster was selected as the best representative for a given putative locus in the final post-processed assembly. Additionally, the longest translatable sequences-per-cluster were selected from non-redundant natively clustered Oases (Oases-VO) and Trinity (Trinity-ICB) assemblies for comparison with assemblies clustered with UCLUST.

#### Completeness of Coverage

A commonly used criterion to assess the optimality of a *de novo* assembled transcriptome of species that has previously determined gene models is how well it recapitulates the models[94]. Using BLASTn[95], with an e-value threshold of 1e-10, each assembly was aligned (using cluster representatives) to the set of 25,512 detected genes as determined by read mapping (described above). Only the most significant locus (least e-value) for each assembled sequence was recorded (with the exception of alignments for Type II error rate evaluation, where two most significant loci recorded). Using custom scripts the alignments were parsed to compute the breadth of sequence coverage, mismatch and gap-opening rates, Type I and Type II error rates across gene models[57]. For each reference gene model, the breadth of sequence coverage was determined by computing the percentage of bases covered by unigenes from each assembly. The mismatch and gap-opening rates for each assembly was determined by computing the ratio of mismatches to total aligned bases and the ratio of gap-openings to total aligned bases respectively.

#### Type I error estimation

Contigs corresponding to the same locus, but which fail to assemble together were classified as Type I errors. We quantified these error rates in each assembly by determining regions with no overlap (i.e. gap between scaffolds not spanned by unigenes, Case I), insufficient overlap regions (overlap < *k-value*, Case II), and regions with conflicting overlaps (≥*k-value—1*, Case III) in BLASTn alignments of unigenes for each locus as (see S2 Fig)

#### Type II error estimation

Mis-assemblies (Type II errors) in each assembly were quantified by determining the ratio of all ambiguously aligned sequences to all reference-aligned unigenes. We determined potential mis-assembled unigenes in the BLASTn alignments if, with at least 99% sequence identity, different segments of an assembled sequence align to at least two non-adjacent annotated gene loci (= “genes”) (Case I), an assembled sequence aligns equally to two genes (Case II), an assembled sequence aligns to a gene model and its subsequence aligns to a different gene (Case III), an assembled sequence whose alignments to two gene overlap with more than 80% of the sequence length (Case IV), and an assembled sequence whose alignments to two genes overlap with at most 80% of the sequence length (Case V) as illustrated in S2 Fig.

We verified that excluding adjacent genes was appropriate by manually examining all unigenes with Type II errors (hits to adjacent *and* non-adjacent loci) in the BR1 CLCscaf primary assembly (See S2 File). With BLASTn we aligned each unigene to the *Arabidopsis* genomic sequences to verify that adjacent genes were accurately co-assembled (i.e. 1 unigene with 2 intact, accurate, and correctly oriented ORFs). After excluding poor alignments (<90% identify, >1e-10 e-value) the extensive manual curation of these alignments revealed that, predominantly, Type II Case 1 errors identified chimeras, whereas Cases II-V did not (S2 File). Thus, using the manually curated learning data, we designed and ran a custom Perl script (S2 File) to parse *Arabidopsis* genomic alignments of all Type II Case 1 BR1 post-processed unigenes to identify chimeras. These results agreed very well (R^2^ = 0.87) with our original estimates of Type II Case 1 errors. In fact, our algorithm to identify Type II errors reports an overestimate of true chimeric unigenes.

#### Assembly Read Mapping Evaluation

The proportion of reads that can map back to an assembly is a useful criterion for the quality assessment of a given *de novo* assembly. Bowtie was used to map Illumina paired-end reads back their respective assemblies retaining only one best alignment for each read. The alignments were then parsed to determine the number of reads that mapped to each assembled sequence, and subsequently used the read mapping results for RNA-Seq expression analysis[64, 70] and generation of gene accumulation curves[41].

#### Read count estimation

The number of reads that mapped to unigenes (scaffolds or contigs) in an assembly were binned for sequences corresponding to a single locus based on BLASTN results to TAIR10. The cumulative log-transformed read counts for each *de novo* assembly and the reference-based assembly for that data set were correlated using the Spearman’s Rank

#### Read Titration Accumulation curves

To generate the gene accumulation curve for a transcriptome assembly, reads were mapped back to the assembly using Bowtie[87] retaining only one best alignment for each read as one would typically do in an RNA-Seq experiment. The frequency distribution of mapped reads in the assembly was used (i.e. number of reads per scaffold) to calculate the rate of new gene detection by randomly sampling reads without replacement and recording the total number of scaffolds detected. Data points were recorded every 1000 reads samples and this process continued until all reads mapping to the assembly examined. The total number of mapped reads and assembled scaffolds was recorded as the end point. This procedure was automated using a modification of the script published by Der et al.[41].

#### Transcript length distribution and assembly size

The length distribution of assembled transcripts and the total size of the assembly (Mbp) reconstructed in a *de novo* assembly is another criterion that can be use to evaluate a *de novo* assembly. Short unigenes were filtered from all assemblies in 100 bp intervals from 100 bp to 600 bp, and we then determined N_50_ length and captured mega base pairs at each interval for each assembly.

#### Ks distribution curves

Ks distribution curves were done as described in Jiao et al. 2011[96].

#### Quality versus Depth plots

The BLASTn results (see above “**Completeness of Coverage**”) and read counts (see above “**Assembly Read Mapping Evaluation**”) were used to determine how well each detected transcript was reconstructed in each assembly. For a given locus, the bit score of the best BLASTn alignment produced from a given assembly was normalized by cDNA length producing the value for “BS” (normalized bit score). The expression signal for each detected gene was determined by summing reads from all unigenes that mapped to that locus. The read counts were then normalized by cDNA length to produce the value for “SFB” (sequenced fragments per base pair). This approach excluded alignments of equal or lower quality (≤bit score) than the best alignment for each locus. In this way we simulate the ability to identify a “best hit” when working without a genome reference and can report the ability of each assembler to reconstruct each locus into a single, contiguous and error free unigene. These two values were plotted against each other to show how each assembly accumulates accurate and contiguous unigenes as a function of sequencing depth. The density scatter plots were generated with custom R[97] scripts.

#### AT1G31330.1 reference alignments and assembly

Using the “**Completeness of Coverage**” (see above) BLASTn results, Trinity and Trinity-S BR1 unigenes that had hits to AT1G31330.1 were aligned using the “Multiple Align” function in Geneious5.6.4 [98] with the “Geneious align” option selected. The more numerous collection of CLC and CLC-S BR1 unigenes that had hits to AT1G31330.1 were assembled using the genomic DNA sequence of AT1G31330.1 as a reference. The coding and cDNA sequences were aligned to the assembly using the “Multiple Align” function in Geneious with the “Consensus align” function. Highlighting in each alignment shows agreements with the consensus sequence for each alignment.

#### MEGAN analysis and alignment stringency cutoff

Unigenes that failed to be assigned to a TAIR10 cDNA were queried against NCBI’s non redundant protein sequences database[2] (NR) using BLASTx (e-value 1e-10, tabular output format). Taxon IDs from NCBI’s Taxomony Browser[99] were appended to the tabular BLAST output with a custom Perl script (v5.12.3). The tabular BLASTx output plus Taxon IDs was imported into MEGAN[72] (Min. support = 1) with Min. Score values ranging from alignment bit scores of 25–250. The frequency of plant (Viridiplantae with subcategories *Arabidopsis* and Other Green Plants) vs. non-assignment was plotted to determine the optimum bit score cutoff that would retain the greatest number of plant hits while maximizing the frequency of non-assignment. The bit score thresholds of 125 and 175 were set as two useful thresholds for classification of *de novo* assembled unigenes. 125 allows roughly double the number of plant hits as 175. The 175 threshold is stricter in that the frequency of non-assignment has flattened out while at the same time excluding many alignments to plant sequences.

## Supporting Information

S1 Fig Summary diagram of assembly error types. Type I assembly reports cases of incomplete assemblies where a given transcript is not assembled into a single sequence (Case I = gap, Case II = Insufficient overlap). Type I error can also consist of failure to bring contigs together (Case III) with sufficient overlap, presumably due to conflict. Type II error reports cases where portions of unigenes have good alignments to >1 TAIR10 cDNAs. Case I more strongly suggests chimerism that Type II cases II-V. Cases II-V essentially report ambiguity in annotation.
(TIFF)

S2 Fig As minimum sequence length cutoffs are imposed the assembly landscape becomes more even. The effect on the N_50_ of assembled sequence length and N_50_ of Mbp of assembled sequence resulting from sequence length cutoffs (imposed at 100–600 bp) for the post-processed assemblies of Illumina biological replicate 1.
(TIFF)

S3 Fig Closely related genes in the “detected gene set” are not efficiently recovered. Gene pairs were identified by a reciprocal best BLASTn hit. Gene number is on the y axis, Ks value of pairs in on the x axis. Equivalent best-fit model components are identified by similar color. “TAIR10” pairs were identified from the comprehensive *Arabidopsis* cDNA collection. The “Detected gene set” pairs were identified from the detected gene set (at lest one tag from any sequencing data set). The remaining plots are of pairs identified from the indicated *de novo* assembly with sequences less than 300 bp removed.
(TIFF)

S4 Fig Gene pairs with higher Ks are less likely to be co-expressed. The frequency of pairs with increasing Ks values were plotted revealing that pairs with lower Ks values were more likely to have expression sufficient (BS >0.1) for assembly of both pairs. Yet pairs with higher Ks values were more likely to have one mate with reads *insufficient* for assembly.
(TIFF)

S5 Fig The recovery of EGPs follows a pronounced hit/no hit pattern. Bit Score (BS) frequency histogram and assembly summary table for Expressed Gene Pairs (EGPs). 473 gene pairs present in the Ks plot of the “Detected gene sets” (see S3 Fig) were absent in the Ks plot of the Mosaik assembly of BR1. For each assembly of BR1 the BS of each mate (946 genes) was plotted. Below the plot is a summary table of the fate of the 473 gene pairs absent in the Mosaik assembly that were present in the “Detected gene set” list.
(TIFF)

S6 Fig The coverage of *Arabidopsis* cDNAs shows a subtle gradation of assembly completeness. Unigenes were aligned to detected gene cDNAs to determine coverage, which was expressed as the percent of cDNA bases covered by assembled sequence. The darkest bar is 0% or “No Hit” and each progressively lighter bar is a bin containing genes covered in 10% increments, with the last two bars representing the number of genes covered at >90% and >99%, respectively.
(TIFF)

S7 Fig Alignment comparison of CLC and Trinity (Inchworm) unigenes representing the AT1G31330.1 transcript. The Sequence order from the top in each alignment (A-D) is gDNA (with a single intron—colored gray), cDNA, CDS and then unigene(s). **A)** Alignment of AT1G31330 reference sequences and the Inchworm BR1 unigenes (x2) annotated as AT1G31330. **B)** Alignment of AT1G31330 reference sequences and the Trinity-ICB BR1 unigene sequence annotated as AT1G31330. **C)** Alignment of AT1G31330 reference sequences and the CLC BR1 unigenes (x623) annotated as AT1G31330. **D)** Alignment of AT1G31330 reference sequences and the CLC-S BR1 unigenes (x96) annotated as AT1G31330. For this highly expressed gene, Trinity is able to distill extensive variation into a single perfect unigene, whereas subsequences with minor differences (often single nucleotides) are maintained as numerous unigenes in the CLC primary and post-processed assemblies, including unigenes that seem to contain introns (middle portion of C and D).
(TIFF)

S8 Fig Normalization does not preferentially remove closely related gene pairs. Scatter plot of read counts to the detected gene set of the Illumina biological replicates 1 and 2 and the normalized Illumina data set. The log_2_ read counts +1 (to avoid taking the log of zero) for each gene were calculated for the Illumina Normalized data set and the Combined (BR12) data set. The “detected gene set” are plotted in gray. The Ultra Conserved Orthologs (UCO, http://compgenomics.ucdavis.edu/.) are plotted in green. The closely related genes set (CRG) are plotted in red.
(TIFF)

S9 Fig The quality of unigenes as a function of sequencing depth for *O*. *sativa*. Publicly available data was retrieved from NCBI’s Sequence Read Archive and assembled with leading the reference based Mosaik and 3 leading *de novo* assemblers. While the data were insufficient to reconstruct a majority of the rice young leaf transcriptome, the inflection point at which higher quality transcripts accumulate is similar to that of the *Arabidopsis* BR1 dataset, indicating that the performance of leading assemblers is similar for rice and *Arabidopsis*.
(TIFF)

S10 Fig Gene expression correlations. **Array BR1 vBR2**: correlation of background corrected, normalized array intensities for biological replicates 1 and 2. **Illumina BR1 v BR2**: correlation of log_2_ read counts (reads +1) from the Illumina biological replicates 1 and 2 mapped to TAIR10 cDNAs. **Array v Ilumina**: correlation of log_2_ read counts (reads +1) mapped at high stringency to the set of array probes and the average, background corrected, normalized array intensities from biological replicates 1 and 2. **Mosaik v Illumina**: correlation of average log_2_ read counts (reads +1) from biological replicates 1 and 2 mapped to the Mosaik-S assembly and the detected gene set. **Trinity-S v Illumina**: correlation of average log_2_ read counts (reads +1) from biological replicates 1 and 2 mapped to the Trinity-S assembly and the detected gene set. **Inchworm-S v Mosaik-S:** correlation of log_2_ read counts (reads +1) from biological replicate 1 mapped to the Trinity-S assembly and the Mosaik-S assembly. Pearson’s R is displayed in the upper left corner of each plot.
(TIFF)

S11 Fig Summary of the 12 candidates chosen for a follow-up analysis by qRT-PCR. The arrows on the plot show the candidates that were chosen for this analysis. The “Probe–cDNA position” columns shows where on the reference cDNA the MicroArray probes hybridized. Generally, the poorly correlated candidates also had a poorer probe set, which may also have contributed to the aberrant signal on the array.
(TIFF)

S12 Fig For well correlated genes all estimates of gene expression show excellent agreement. **(see** S11 Fig**)** Fold difference in expression relative to AtActin (AT3G18780.1) was determined for candidates indicated. Those within the linear portion the Array vs. RNA-Seq correlation with each method as appropriate (S11 Fig). Well correlated qRT-PCR candidates are indicated solid black arrows (S11 Fig). qRT-PCR candidates which extend beyond the range of the array but followed the linear trend are indicated by dashed black arrows (S11 Fig).
(TIFF)

S13 Fig Threshold alignment score of 125 is sufficient to exclude erroneous hits and classifying plant genes. The increase of non-assignment from alignment scores of 125 to 175 is minimal yet the instance of hits to plant genes is also decreased from alignment scores of 125 to 175. Depending on the desired outcome, alignment scores >125 can be used with confidence to exclude erroneous classification while classifying more plant genes.
(TIFF)

S14 Fig MEGAN classification of unigenes that do not align to *Arabidopsis* TAIR10 cDNAs. The classification was determined for unigenes that aligned to sequences in NR with a bit score >125.
(TIFF)

S15 Fig MEGAN classification of unigenes that do not align to *Arabidopsis* TAIR10 cDNAs. The classification was determined for unigenes that aligned to sequences in NR with a bit score ≥175.
(TIFF)

S1 File This file contains statistics for primary and post processed assemblies of BR1, BR2, BR12, and NORM datasets. The change in each category is indicated in a shaded field with a delta sign, i.e. **Mosaik-S Δ.**
(XLSX)

S2 File This archive contains the results of the follow-up Type II error analysis. Only Type II Case 1 errors reliably identified true chimeras (see Training_data_BR1_CLCscaf.xlsx and S2_File_illustrations.pptx). Our follow-up analysis confirms that adjacent loci are co-assembled accurately and are not chimeric unigenes. Typically only a fraction of Type II Case 1 errors are true chimeras.
(ZIP)

S3 File The quality of assembled sequences as a function of sequencing depth for all Illumina assemblies. The units for “Assembly Quality” are Normalized Bit Score (BS, maximum of 2) and the units of “Sequence Depth” are Sequenced Fragments/bp (SFB). The number printed in the plot area is the number of assembled sequences with normalized Bit Score above 1.5. A BS of 1.5 is an arbitrary threshold, yet represents long and accurate assemblies, and is used to illustrate the difference in the high density region seen in most plots near BS 1.75–2. For instance, a transcript that was reconstructed to 75% of the reference cDNA length with no errors would result in an alignment with a BS of 1.5. Since the rate of base call errors, alignment gaps and Type II errors are low, the BS reports primarily alignment length.
(PPTX)

S4 File The poorly correlated candidates (disagreement between the microarray and RNA-Seq) were either cases of false positives on the array (2 of 3) or were erroneous signals from poorly annotated genes. This file details additional efforts to understand the reasons for disagreement between the two analyses. See also S10, S11 and S12 Figs.
(DOCX)

S5 File This files contains a table that summarizes alignment statistics for putative "new" Arabidopsis genes. The 5 columns contain database specific matches, though only the hits to nr were used for MEGAN analysis. Hits in other databases are described as "concordant" evidence in the manuscript text.
(XLSX)

S6 File This archive contains the *SCE*RNA protocol. Necessary scripts are included and URLs for components (or alternatives) are also included. Instructions and scripts for generating plots (e.g. Fig 4) are also included.
(ZIP)

S1 Table Sequencing and alignment statistics for the normalized and non-normalized libraries used in this study. *Percentage of raw reads aligned.
(TIFF)

S2 Table List of suffixes, abbreviations and gene lists. (TIFF)

S3 Table Type II error rates of *Arabidopsis* BR1 assemblies and the Rice young leaf transcriptome assemblies are similar. See S1 Fig for an error diagram. Unigenes from all assemblies are aligned to reference sequences with BLAST to allow for an unbiased estimation of Type II error.
(TIFF)
