# Supplementary figures and images for: Selecting Superior De Novo Transcriptome Assemblies: Lessons Learned by Leveraging the Best Plant Genome
Source: PLoS One. 2016 Jan 5;11(1):e0146062. doi: 10.1371/journal.pone.0146062 (PMC4701411; doi:10.1371/journal.pone.0146062)

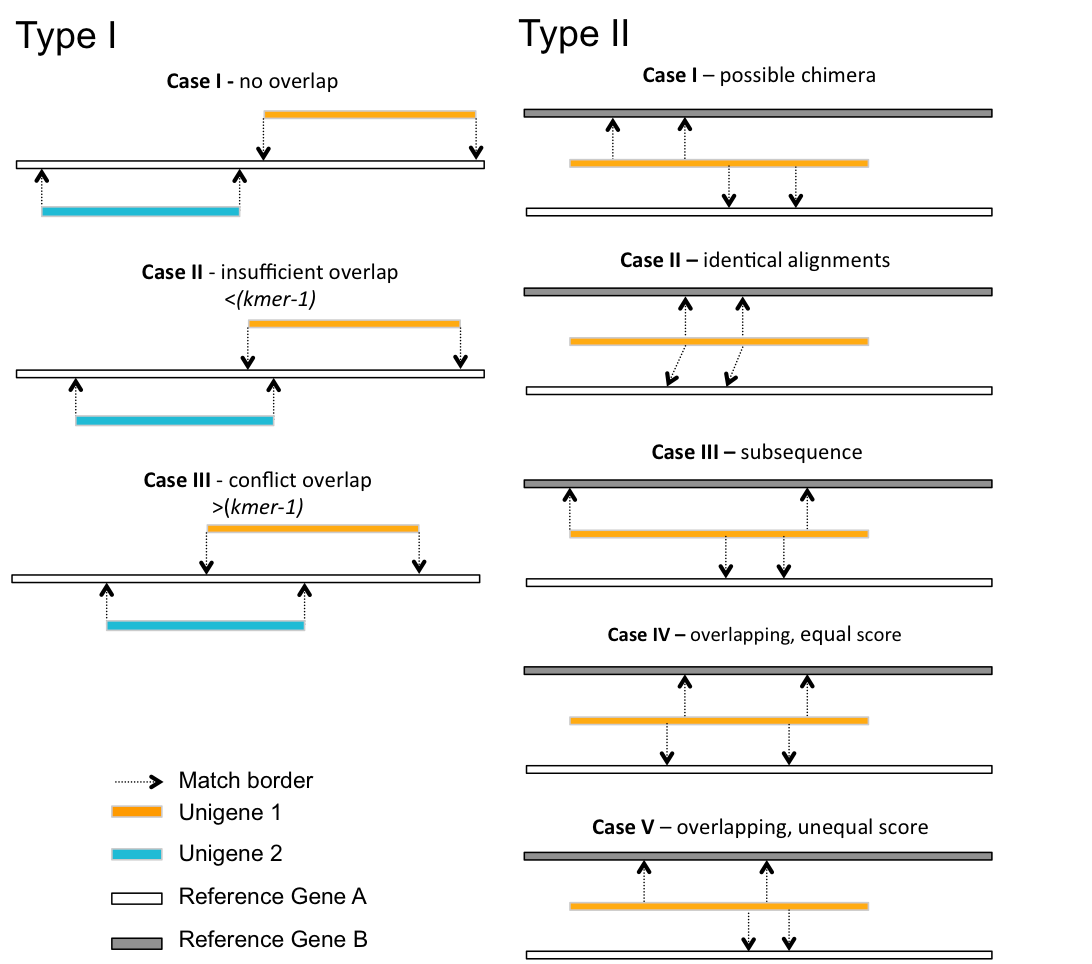

Supplement: S1 Fig — Type I assembly reports cases of incomplete assemblies where a given transcript is not assembled into a single sequence (Case I = gap, Case II = Insufficient overlap). Type I error can also consist of failure to bring contigs together (Case III) with sufficient overlap, presumably due to conflict. Type II error reports cases where portions of unigenes have good alignments to >1 TAIR10 cDNAs. Case I more strongly suggests chimerism that Type II cases II-V. Cases II-V essentially report ambiguity in annotation. (TIFF) [file pone.0146062.s001.tiff]

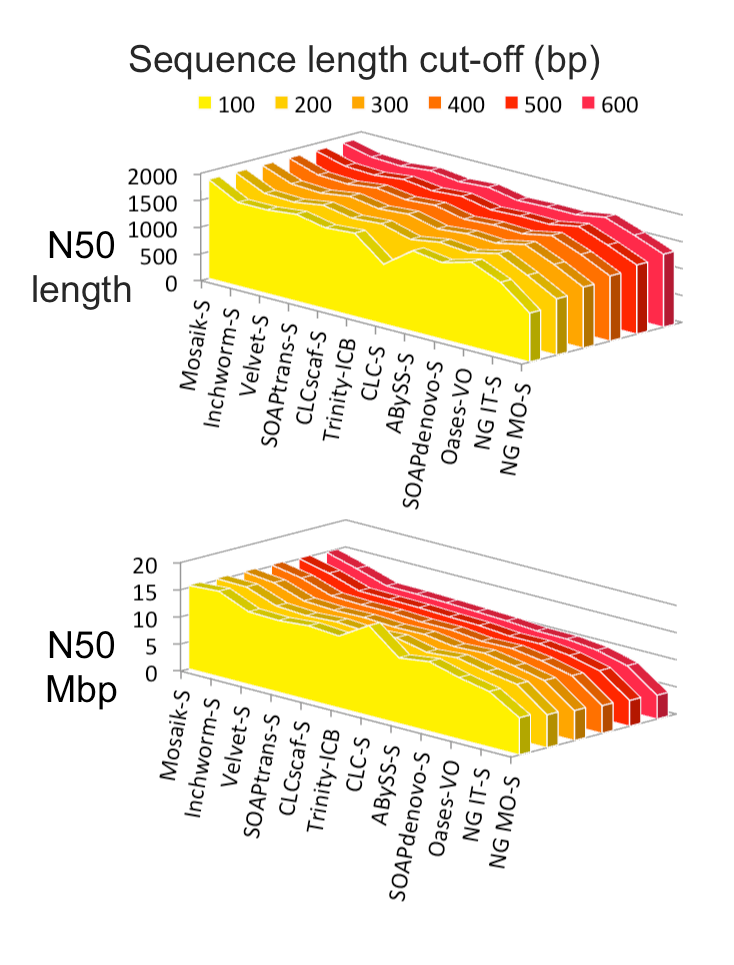

Supplement: S2 Fig — The effect on the N50 of assembled sequence length and N50 of Mbp of assembled sequence resulting from sequence length cutoffs (imposed at 100–600 bp) for the post-processed assemblies of Illumina biological replicate 1. (TIFF) [file pone.0146062.s002.tiff]

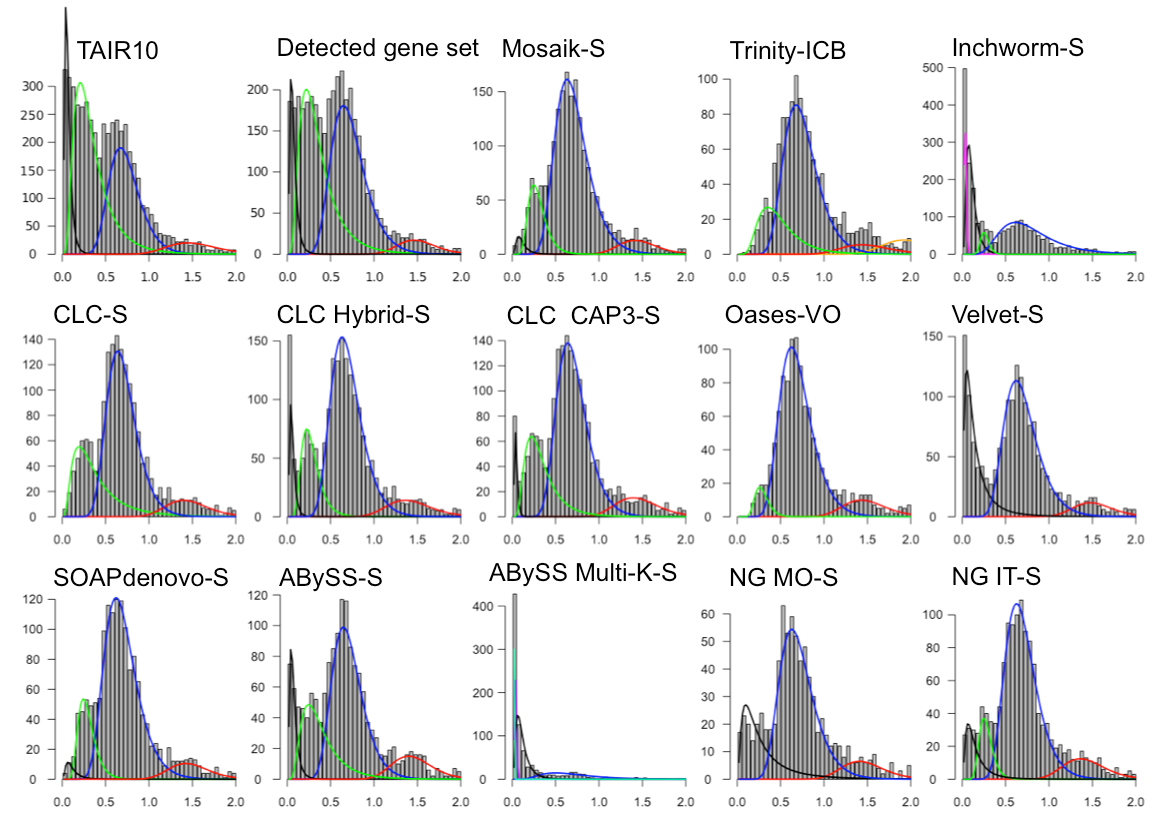

Supplement: S3 Fig — Gene pairs were identified by a reciprocal best BLASTn hit. Gene number is on the y axis, Ks value of pairs in on the x axis. Equivalent best-fit model components are identified by similar color. “TAIR10” pairs were identified from the comprehensive Arabidopsis cDNA collection. The “Detected gene set” pairs were identified from the detected gene set (at lest one tag from any sequencing data set). The remaining plots are of pairs identified from the indicated de novo assembly with sequences less than 300 bp removed. (TIFF) [file pone.0146062.s003.tiff]

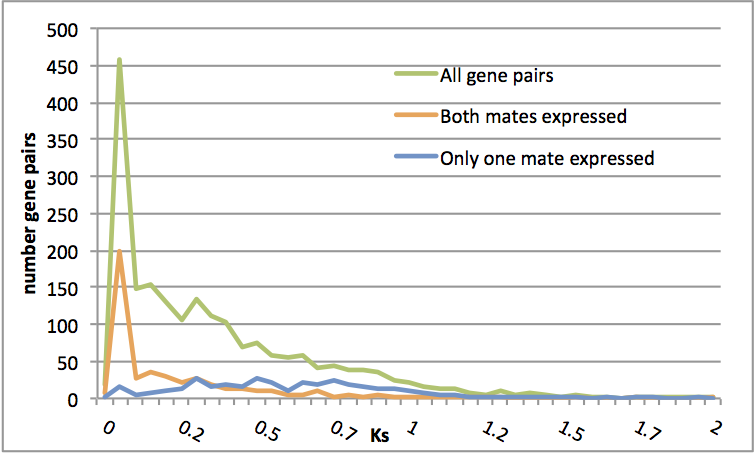

Supplement: S4 Fig — The frequency of pairs with increasing Ks values were plotted revealing that pairs with lower Ks values were more likely to have expression sufficient (BS >0.1) for assembly of both pairs. Yet pairs with higher Ks values were more likely to have one mate with reads insufficient for assembly. (TIFF) [file pone.0146062.s004.tiff]

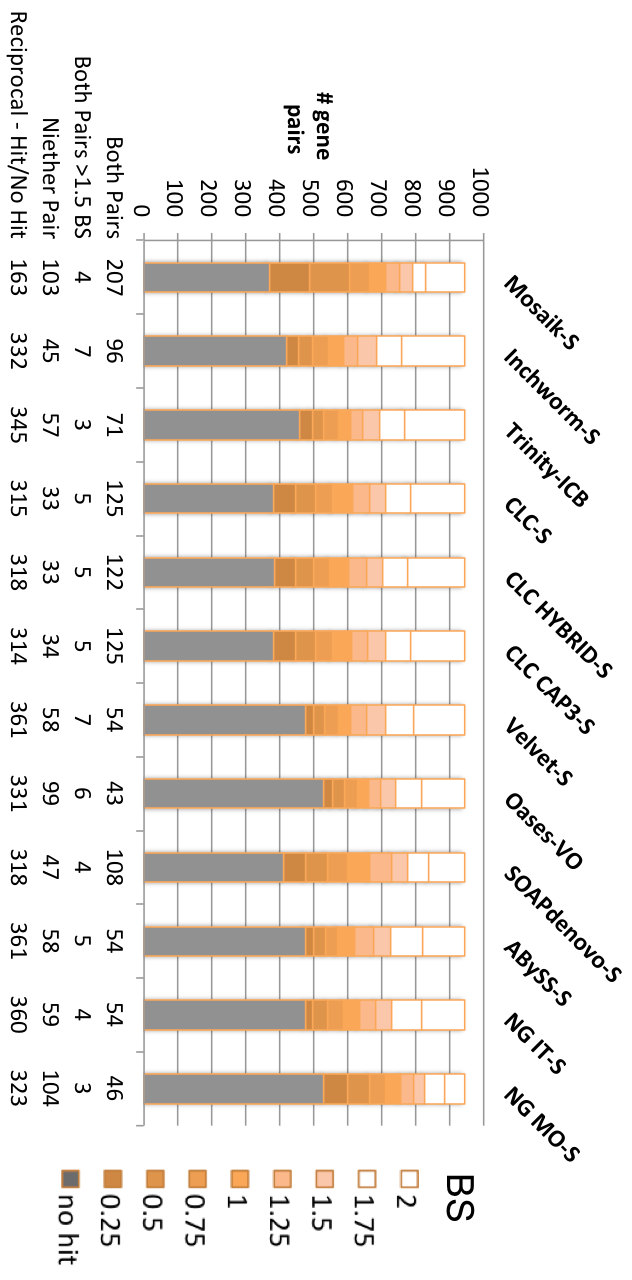

Supplement: S5 Fig — Bit Score (BS) frequency histogram and assembly summary table for Expressed Gene Pairs (EGPs). 473 gene pairs present in the Ks plot of the “Detected gene sets” (see S3 Fig) were absent in the Ks plot of the Mosaik assembly of BR1. For each assembly of BR1 the BS of each mate (946 genes) was plotted. Below the plot is a summary table of the fate of the 473 gene pairs absent in the Mosaik assembly that were present in the “Detected gene set” list. (TIFF) [file pone.0146062.s005.tiff]

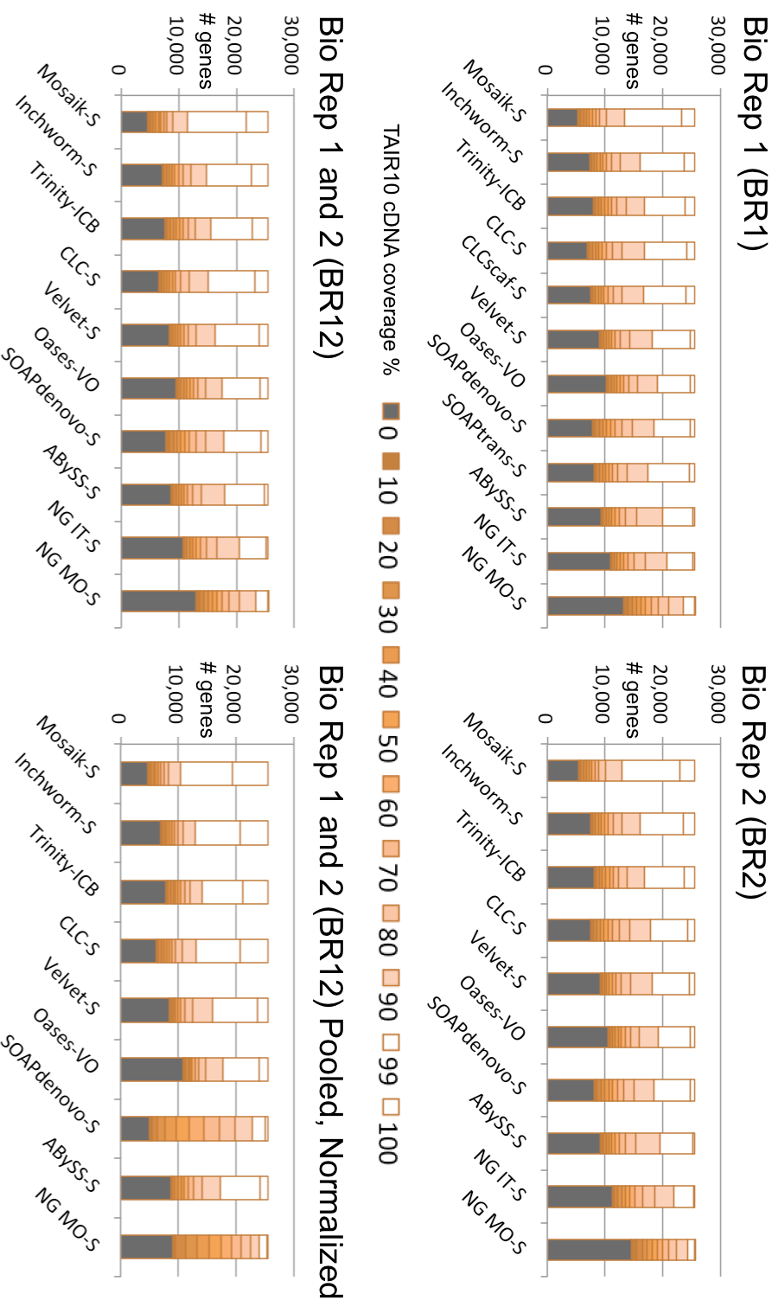

Supplement: S6 Fig — Unigenes were aligned to detected gene cDNAs to determine coverage, which was expressed as the percent of cDNA bases covered by assembled sequence. The darkest bar is 0% or “No Hit” and each progressively lighter bar is a bin containing genes covered in 10% increments, with the last two bars representing the number of genes covered at >90% and >99%, respectively. (TIFF) [file pone.0146062.s006.tiff]

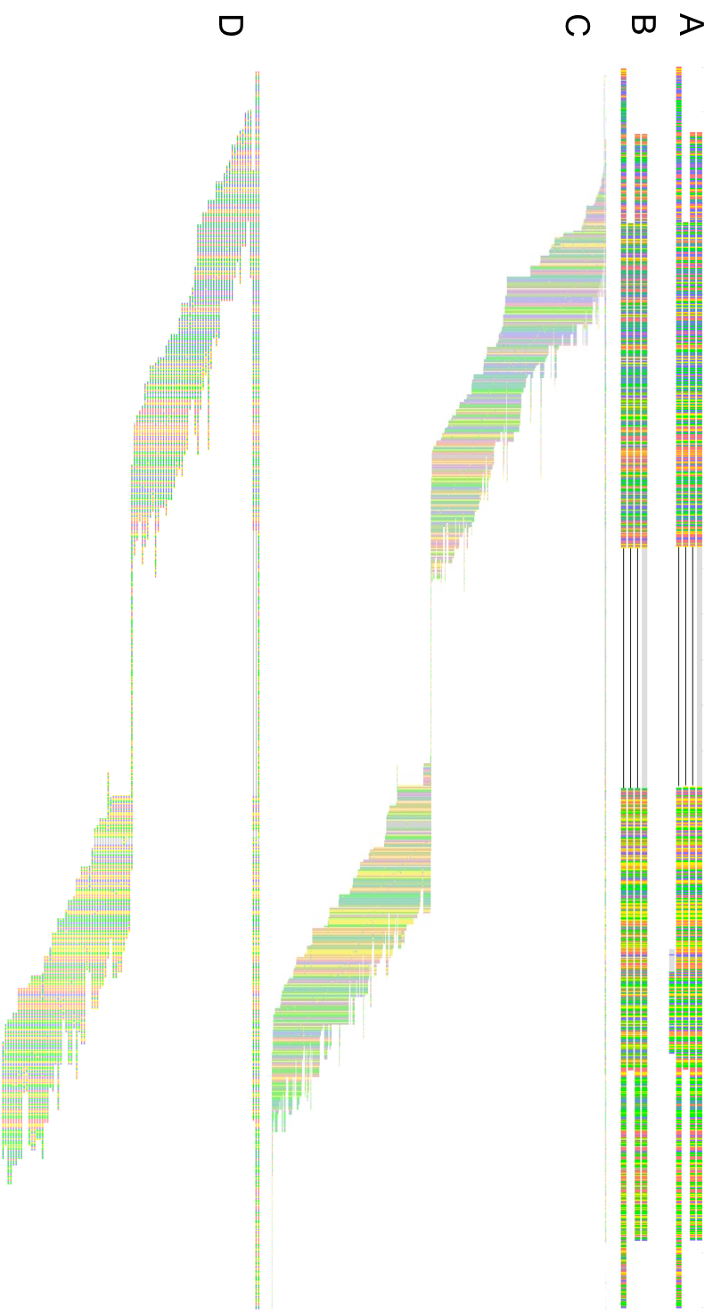

Supplement: S7 Fig — The Sequence order from the top in each alignment (A-D) is gDNA (with a single intron—colored gray), cDNA, CDS and then unigene(s). A) Alignment of AT1G31330 reference sequences and the Inchworm BR1 unigenes (x2) annotated as AT1G31330. B) Alignment of AT1G31330 reference sequences and the Trinity-ICB BR1 unigene sequence annotated as AT1G31330. C) Alignment of AT1G31330 reference sequences and the CLC BR1 unigenes (x623) annotated as AT1G31330. D) Alignment of AT1G31330 reference sequences and the CLC-S BR1 unigenes (x96) annotated as AT1G31330. For this highly expressed gene, Trinity is able to distill extensive variation into a single perfect unigene, whereas subsequences with minor differences (often single nucleotides) are maintained as numerous unigenes in the CLC primary and post-processed assemblies, including unigenes that seem to contain introns (middle portion of C and D). (TIFF) [file pone.0146062.s007.tiff]

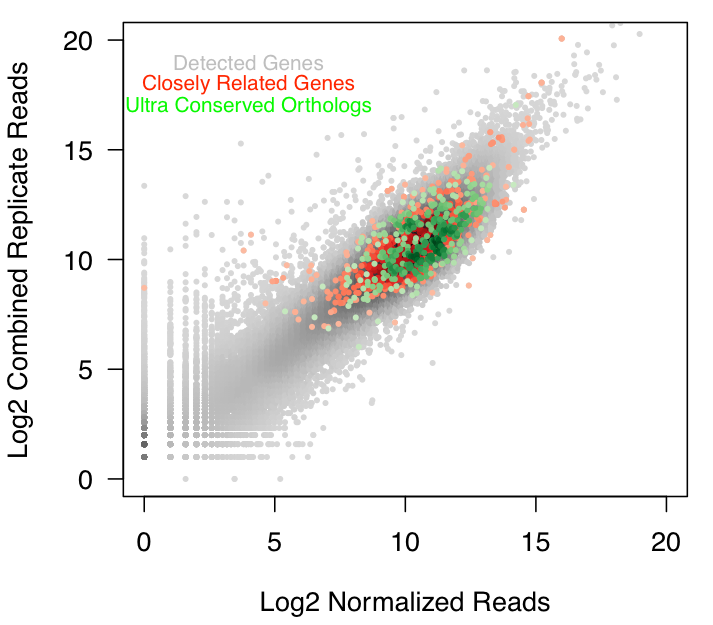

Supplement: S8 Fig — Scatter plot of read counts to the detected gene set of the Illumina biological replicates 1 and 2 and the normalized Illumina data set. The log2 read counts +1 (to avoid taking the log of zero) for each gene were calculated for the Illumina Normalized data set and the Combined (BR12) data set. The “detected gene set” are plotted in gray. The Ultra Conserved Orthologs (UCO, http://compgenomics.ucdavis.edu/.) are plotted in green. The closely related genes set (CRG) are plotted in red. (TIFF) [file pone.0146062.s008.tiff]

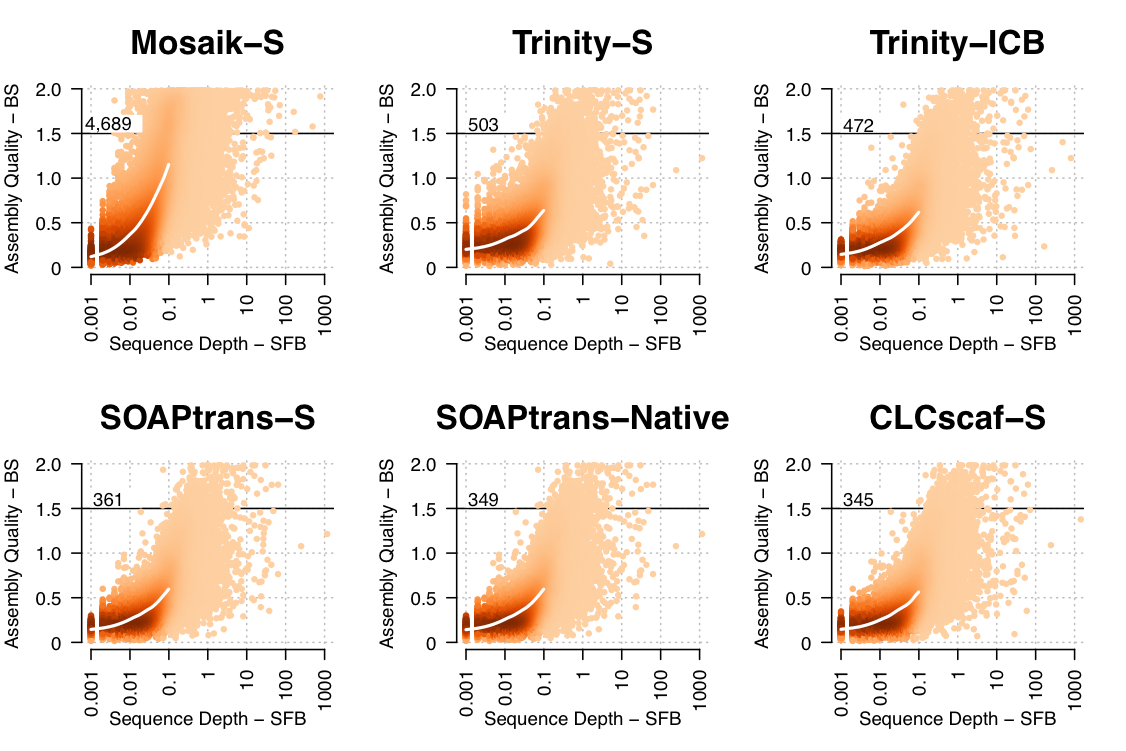

Supplement: S9 Fig — Publicly available data was retrieved from NCBI’s Sequence Read Archive and assembled with leading the reference based Mosaik and 3 leading de novo assemblers. While the data were insufficient to reconstruct a majority of the rice young leaf transcriptome, the inflection point at which higher quality transcripts accumulate is similar to that of the Arabidopsis BR1 dataset, indicating that the performance of leading assemblers is similar for rice and Arabidopsis. (TIFF) [file pone.0146062.s009.tiff]

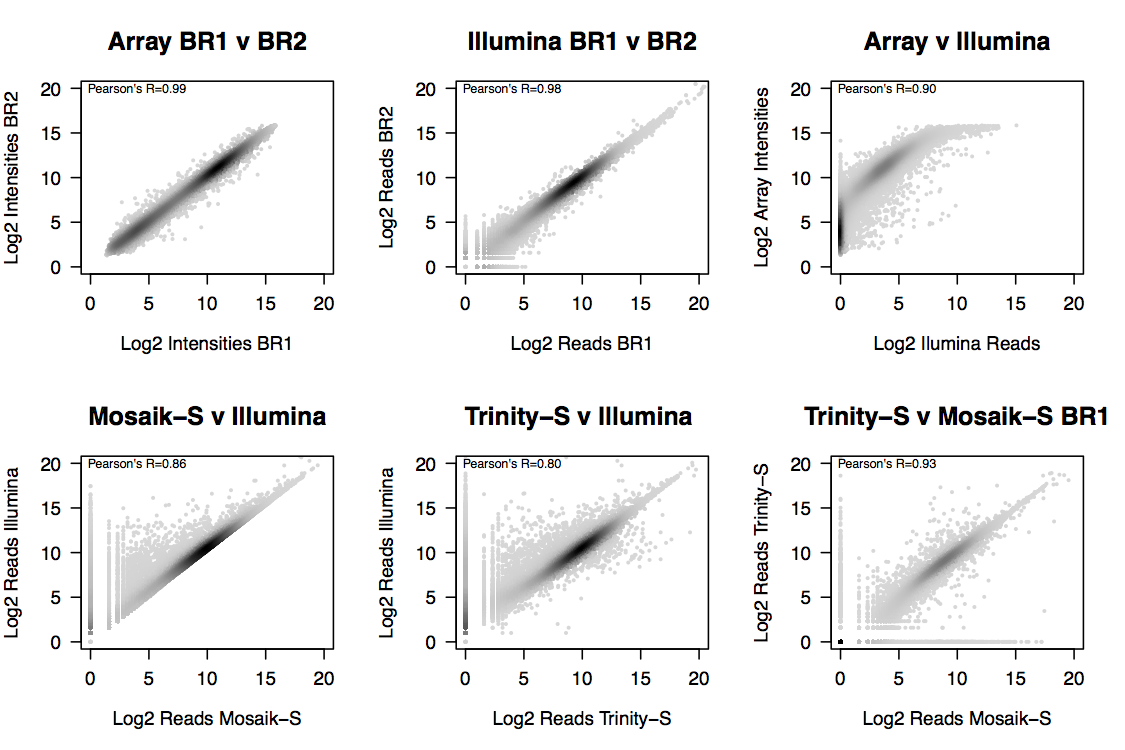

Supplement: S10 Fig — Array BR1 vBR2: correlation of background corrected, normalized array intensities for biological replicates 1 and 2. Illumina BR1 v BR2: correlation of log2 read counts (reads +1) from the Illumina biological replicates 1 and 2 mapped to TAIR10 cDNAs. Array v Ilumina: correlation of log2 read counts (reads +1) mapped at high stringency to the set of array probes and the average, background corrected, normalized array intensities from biological replicates 1 and 2. Mosaik v Illumina: correlation of average log2 read counts (reads +1) from biological replicates 1 and 2 mapped to the Mosaik-S assembly and the detected gene set. Trinity-S v Illumina: correlation of average log2 read counts (reads +1) from biological replicates 1 and 2 mapped to the Trinity-S assembly and the detected gene set. Inchworm-S v Mosaik-S: correlation of log2 read counts (reads +1) from biological replicate 1 mapped to the Trinity-S assembly and the Mosaik-S assembly. Pearson’s R is displayed in the upper left corner of each plot. (TIFF) [file pone.0146062.s010.tiff]

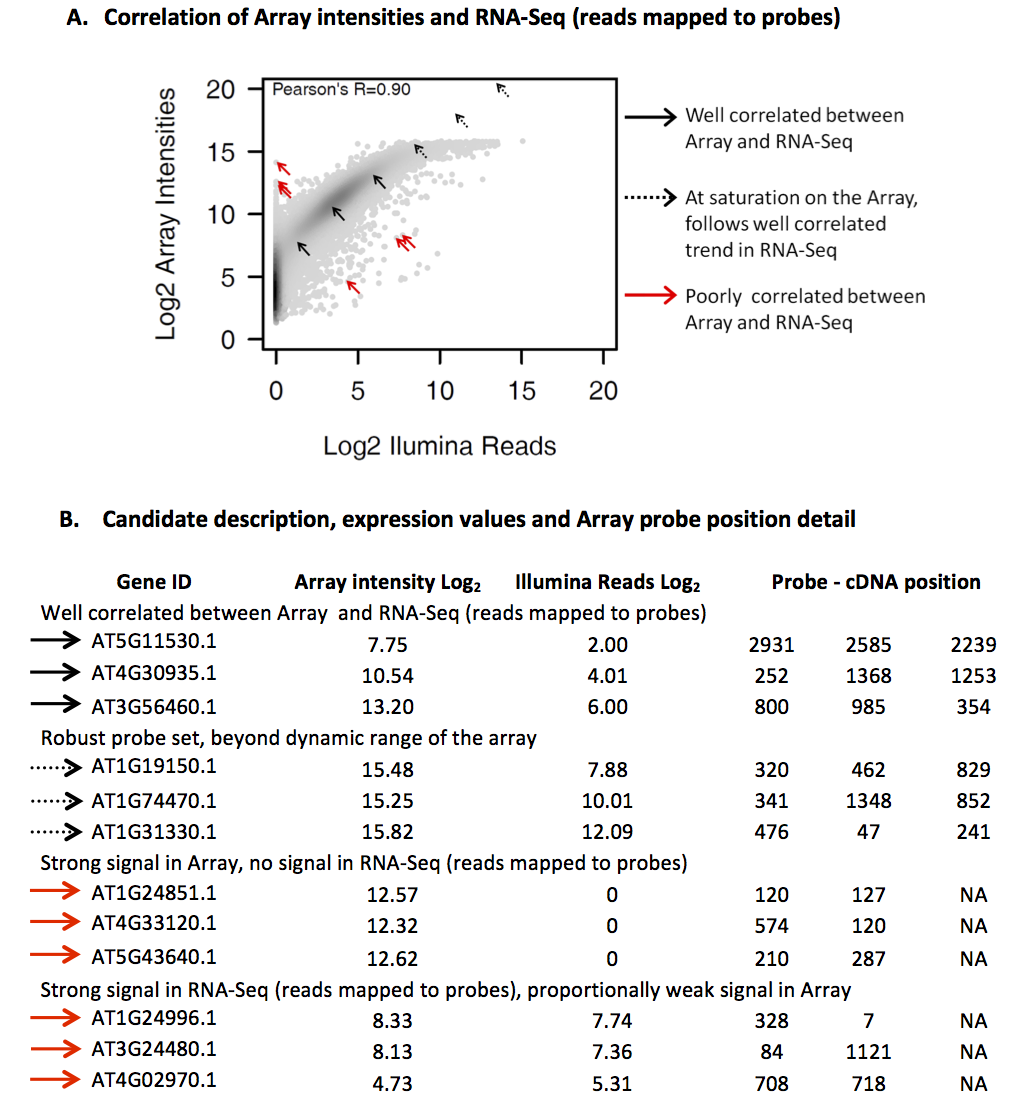

Supplement: S11 Fig — The arrows on the plot show the candidates that were chosen for this analysis. The “Probe–cDNA position” columns shows where on the reference cDNA the MicroArray probes hybridized. Generally, the poorly correlated candidates also had a poorer probe set, which may also have contributed to the aberrant signal on the array. (TIFF) [file pone.0146062.s011.tiff]

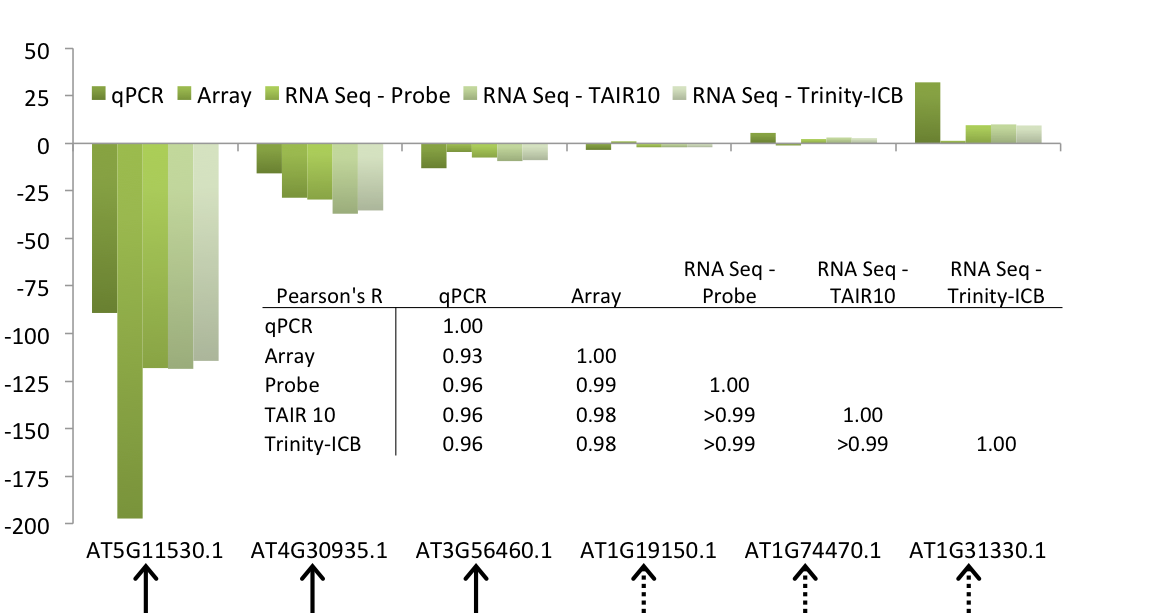

Supplement: S12 Fig — (see S11 Fig) Fold difference in expression relative to AtActin (AT3G18780.1) was determined for candidates indicated. Those within the linear portion the Array vs. RNA-Seq correlation with each method as appropriate (S11 Fig). Well correlated qRT-PCR candidates are indicated solid black arrows (S11 Fig). qRT-PCR candidates which extend beyond the range of the array but followed the linear trend are indicated by dashed black arrows (S11 Fig). (TIFF) [file pone.0146062.s012.tiff]

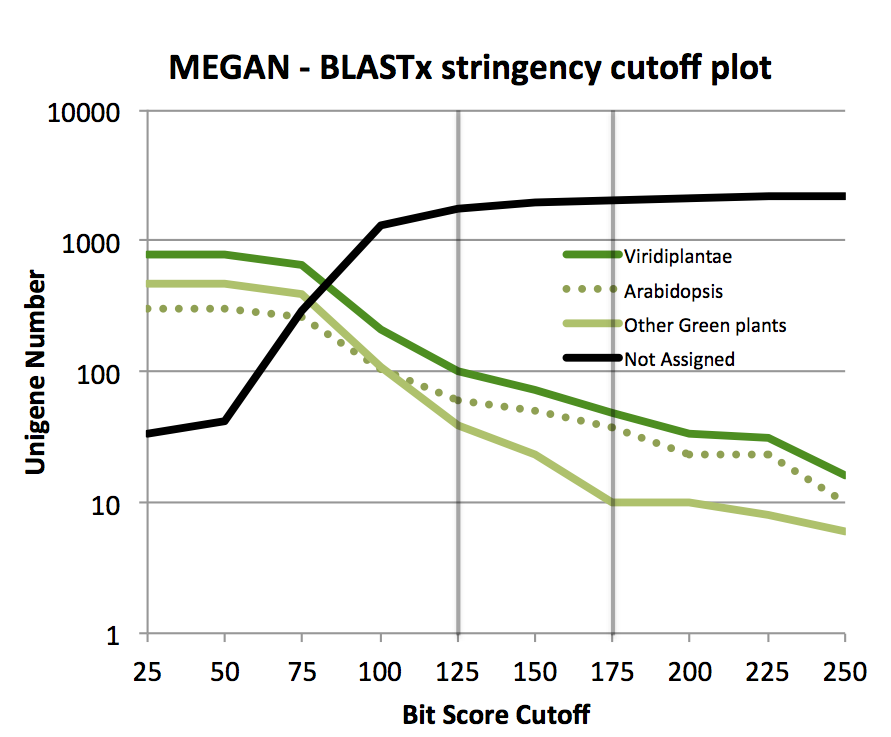

Supplement: S13 Fig — The increase of non-assignment from alignment scores of 125 to 175 is minimal yet the instance of hits to plant genes is also decreased from alignment scores of 125 to 175. Depending on the desired outcome, alignment scores >125 can be used with confidence to exclude erroneous classification while classifying more plant genes. (TIFF) [file pone.0146062.s013.tiff]

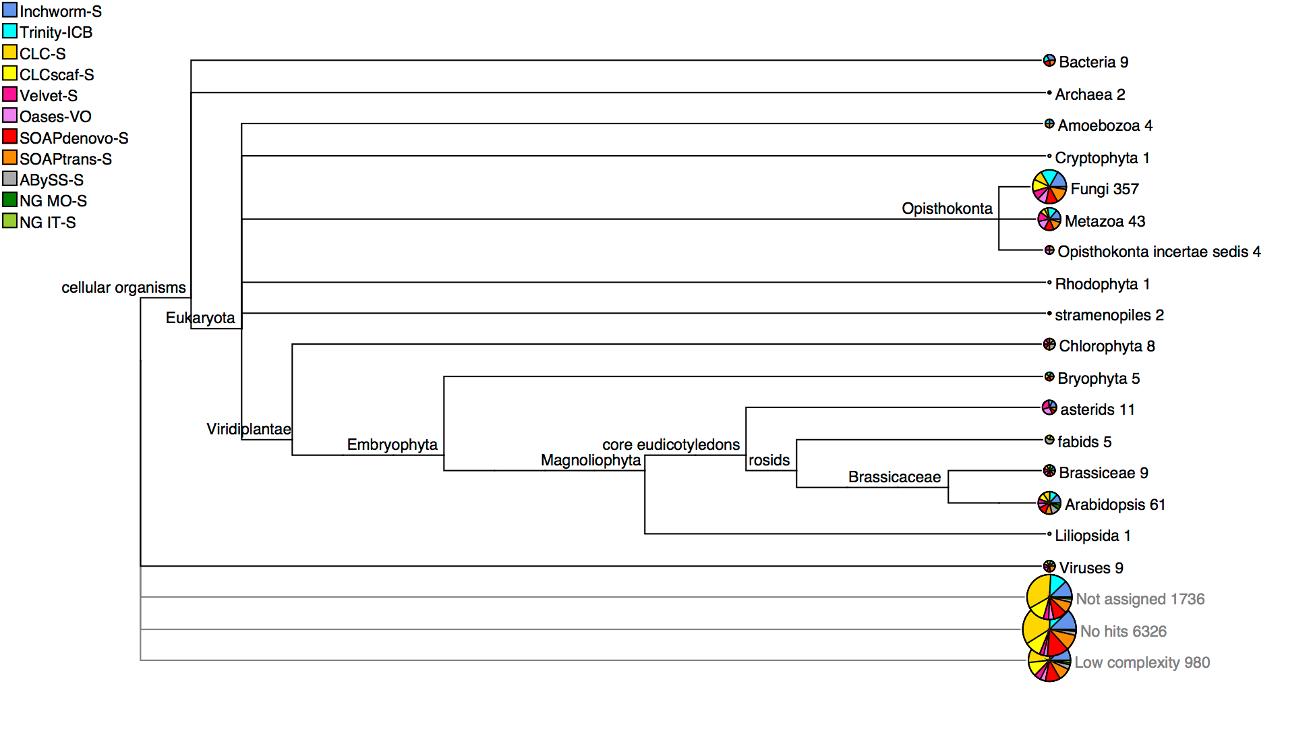

Supplement: S14 Fig — The classification was determined for unigenes that aligned to sequences in NR with a bit score >125. (TIFF) [file pone.0146062.s014.tiff]

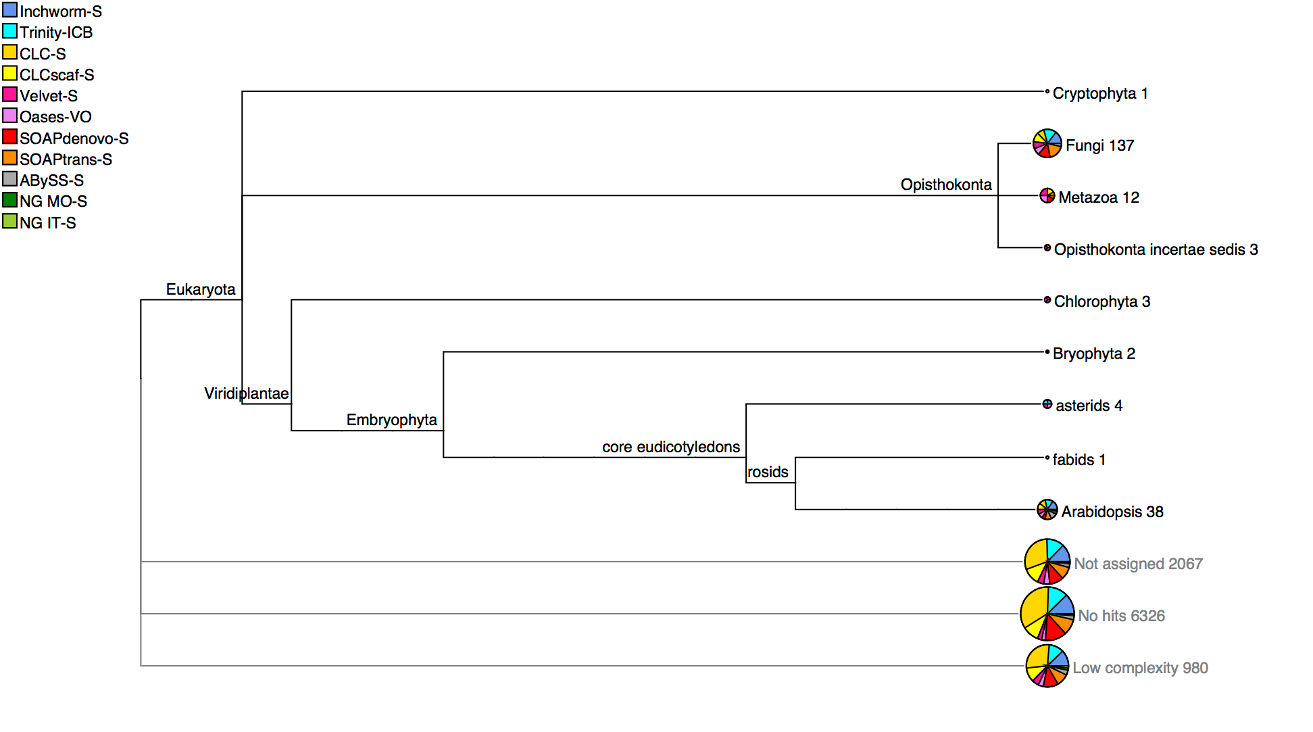

Supplement: S15 Fig — The classification was determined for unigenes that aligned to sequences in NR with a bit score ≥175. (TIFF) [file pone.0146062.s015.tiff]

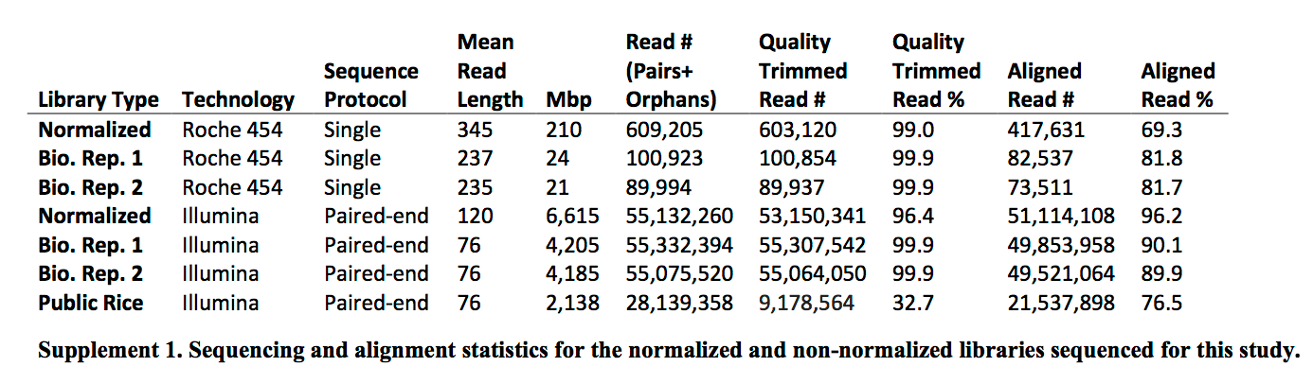

Supplement: S1 Table — *Percentage of raw reads aligned. (TIFF) [file pone.0146062.s022.tiff]

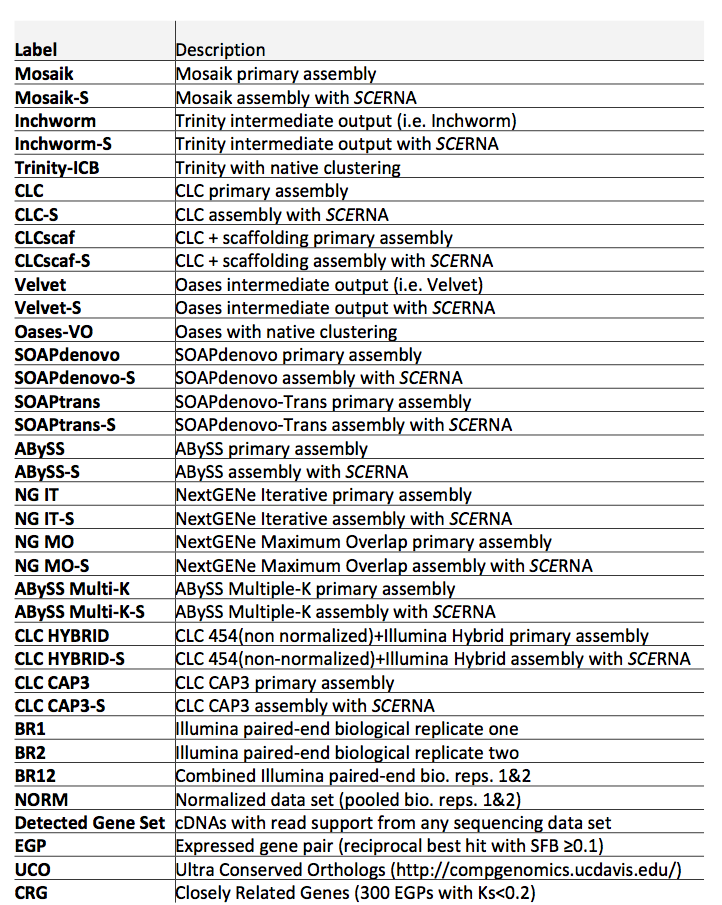

Supplement: S2 Table — (TIFF) [file pone.0146062.s023.tiff]

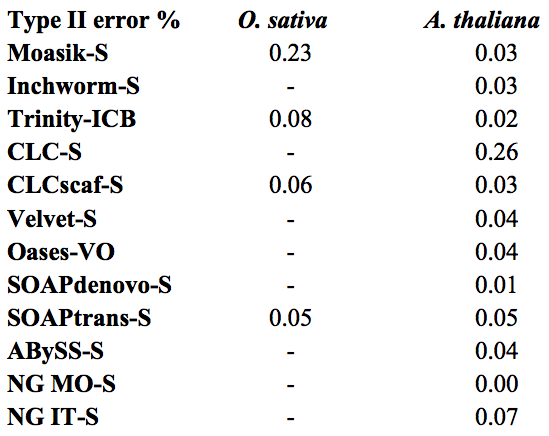

Supplement: S3 Table — See S1 Fig for an error diagram. Unigenes from all assemblies are aligned to reference sequences with BLAST to allow for an unbiased estimation of Type II error. (TIFF) [file pone.0146062.s024.tiff]
