# Supplementary material for: Selecting Superior De Novo Transcriptome Assemblies: Lessons Learned by Leveraging the Best Plant Genome
Source: PLoS One. 2016 Jan 5;11(1):e0146062. doi: 10.1371/journal.pone.0146062 (PMC4701411; doi:10.1371/journal.pone.0146062)
Supplement: S2 File — Only Type II Case 1 errors reliably identified true chimeras (see Training_data_BR1_CLCscaf.xlsx and S2_File_illustrations.pptx). Our follow-up analysis confirms that adjacent loci are co-assembled accurately and are not chimeric unigenes. Typically only a fraction of Type II Case 1 errors are true chimeras. (ZIP) [file pone.0146062.s017.zip › Supplemental_File2/S2_File_illustrations.pptx]

## Slide 1
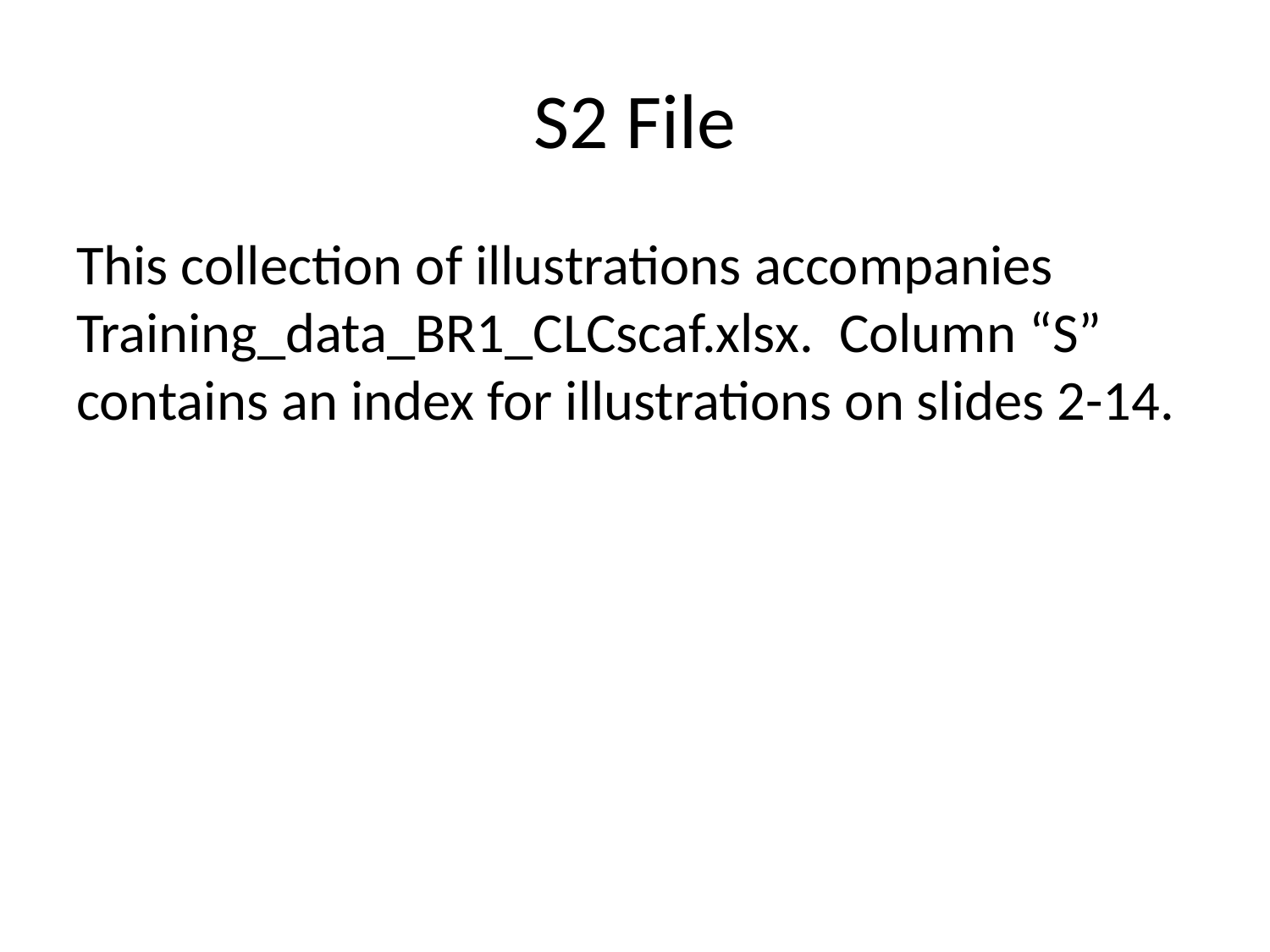

# S2 File
This collection of illustrations accompanies Training_data_BR1_CLCscaf.xlsx. Column “S” contains an index for illustrations on slides 2-14.

## Slide 2
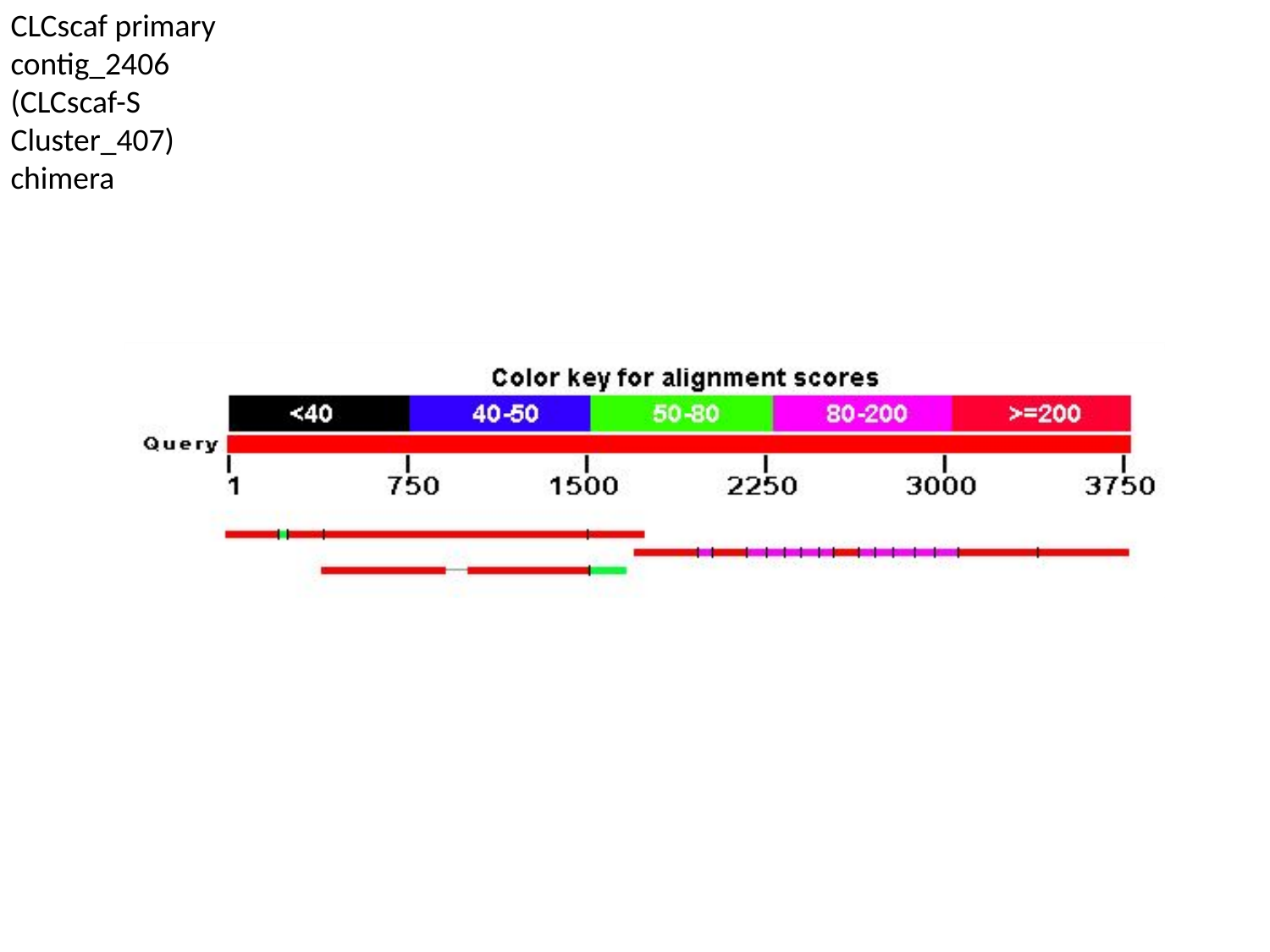

CLCscaf primary
contig_2406
(CLCscaf-S
Cluster_407)
chimera

## Slide 3
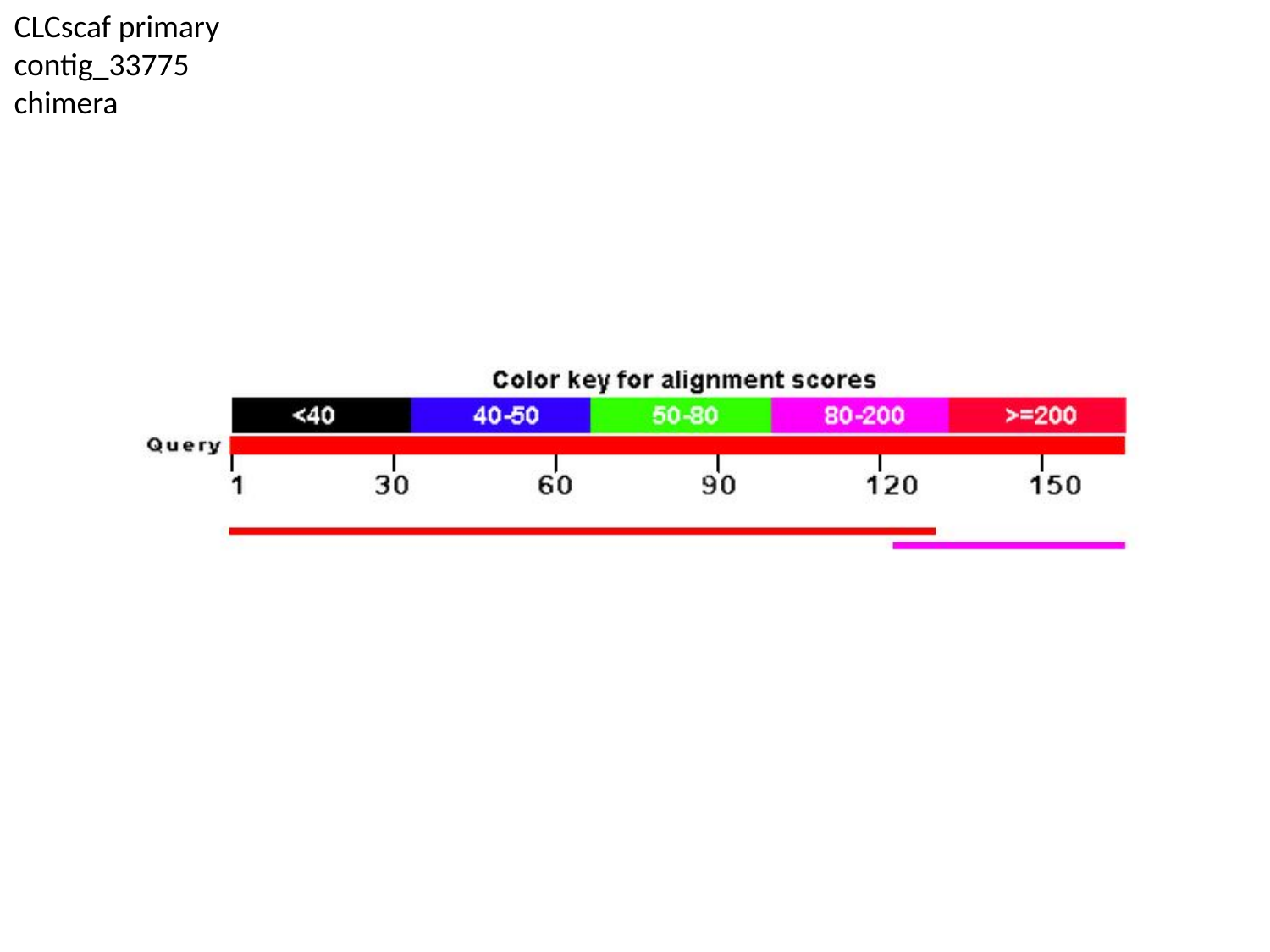

CLCscaf primary
contig_33775
chimera

## Slide 4
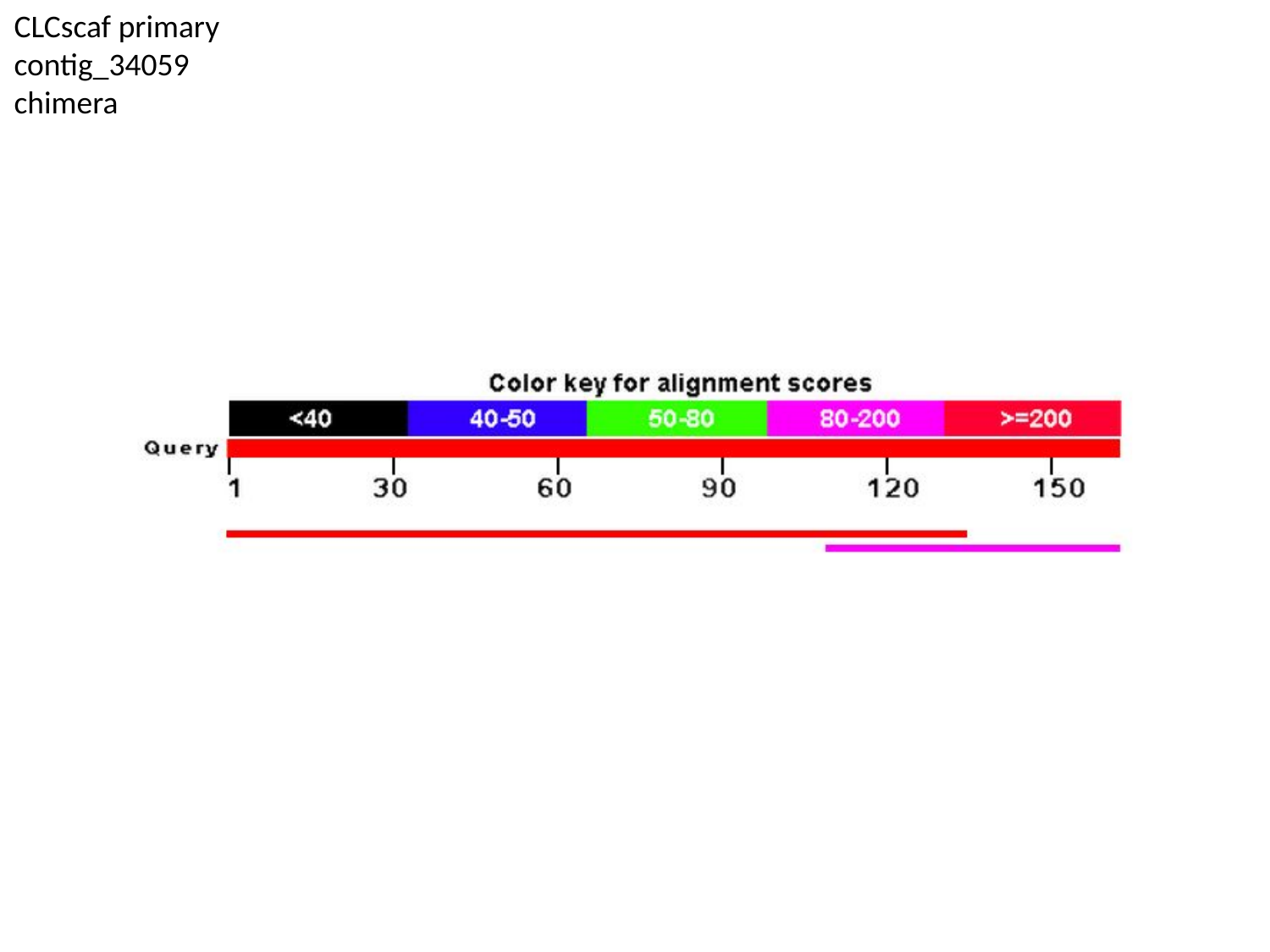

CLCscaf primary
contig_34059
chimera

## Slide 5
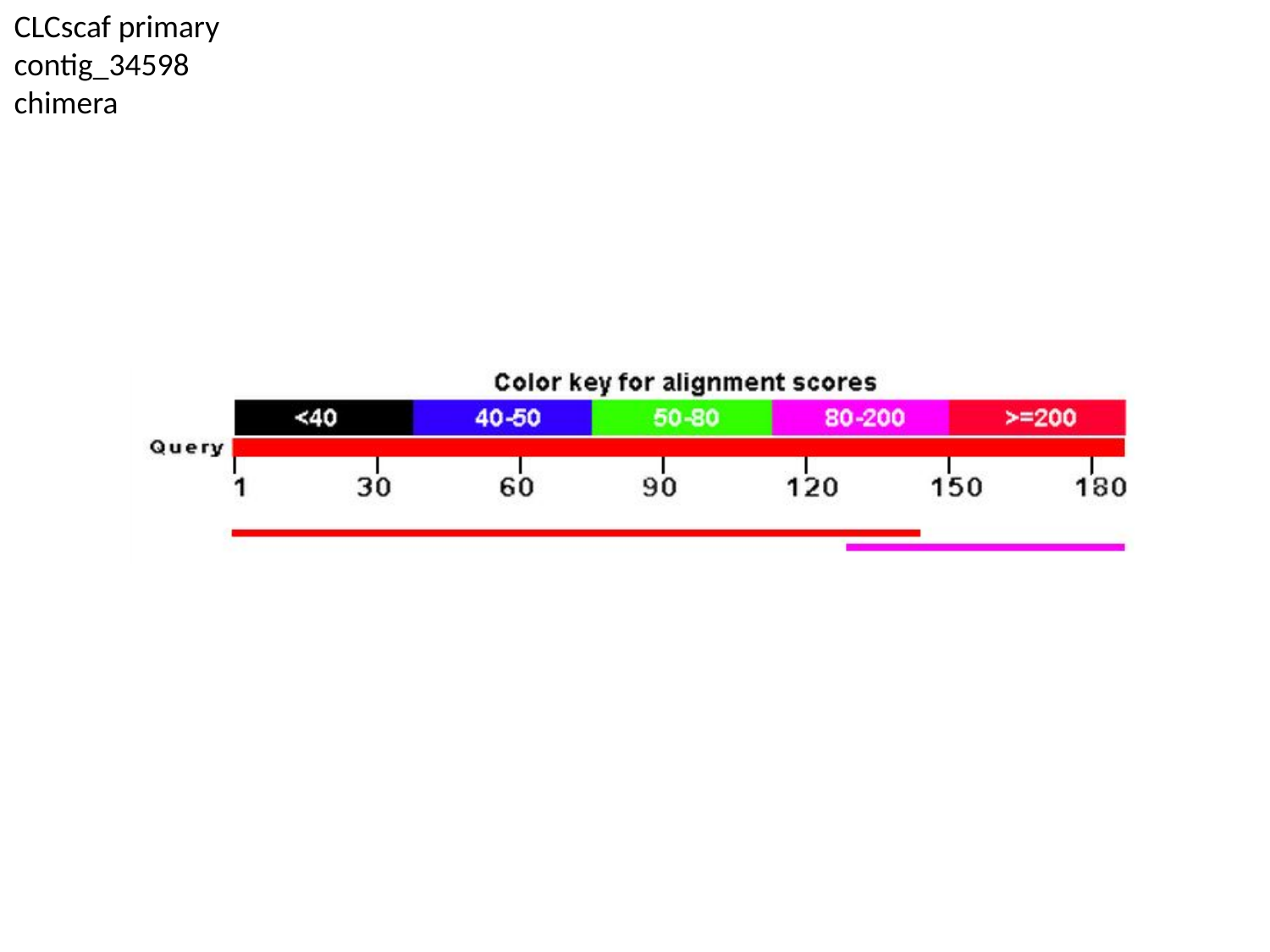

CLCscaf primary
contig_34598
chimera

## Slide 6
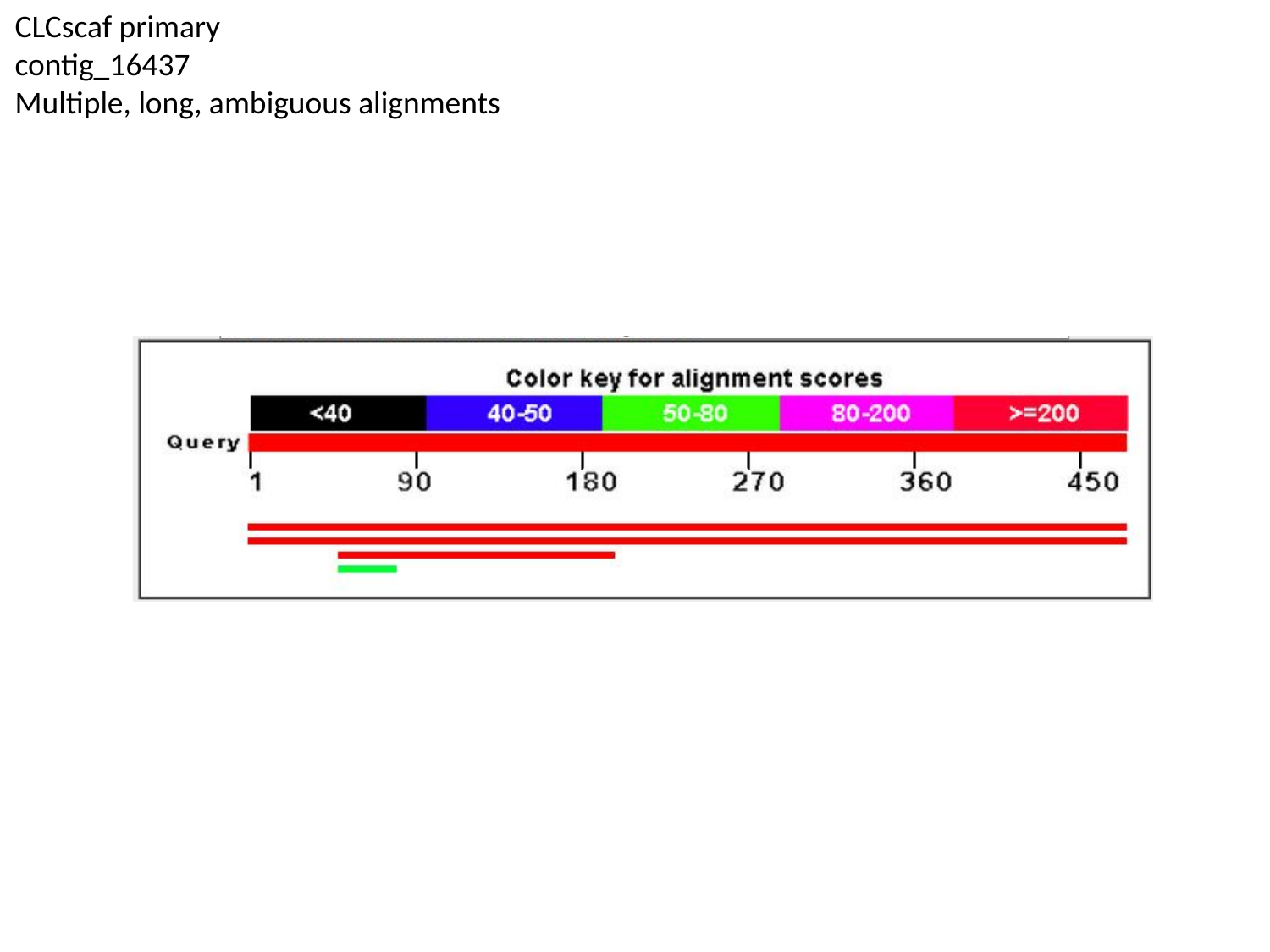

CLCscaf primary
contig_16437
Multiple, long, ambiguous alignments

## Slide 7
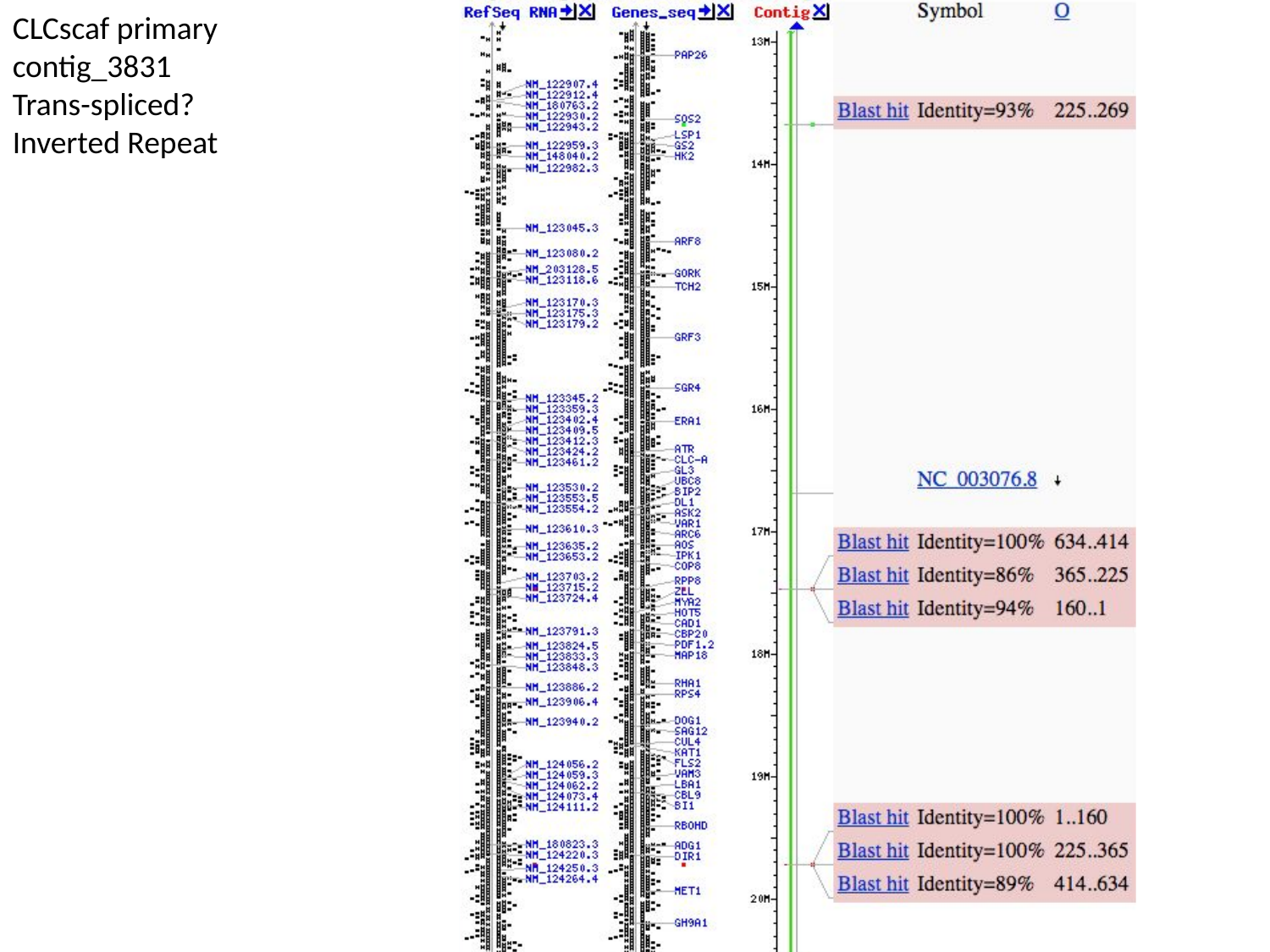

CLCscaf primary
contig_3831
Trans-spliced?
Inverted Repeat

## Slide 8
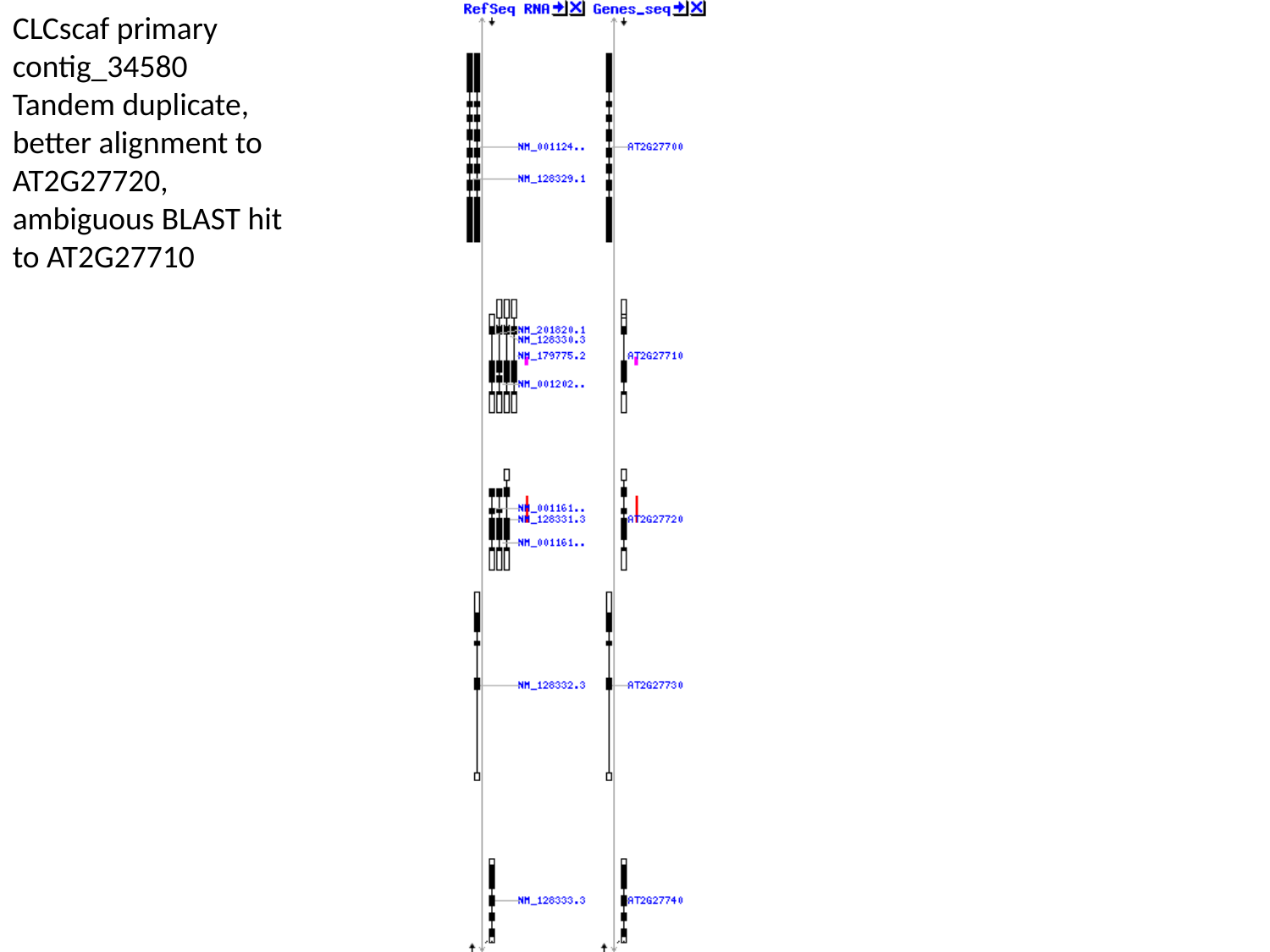

CLCscaf primary
contig_34580
Tandem duplicate, better alignment to AT2G27720, ambiguous BLAST hit to AT2G27710

## Slide 9
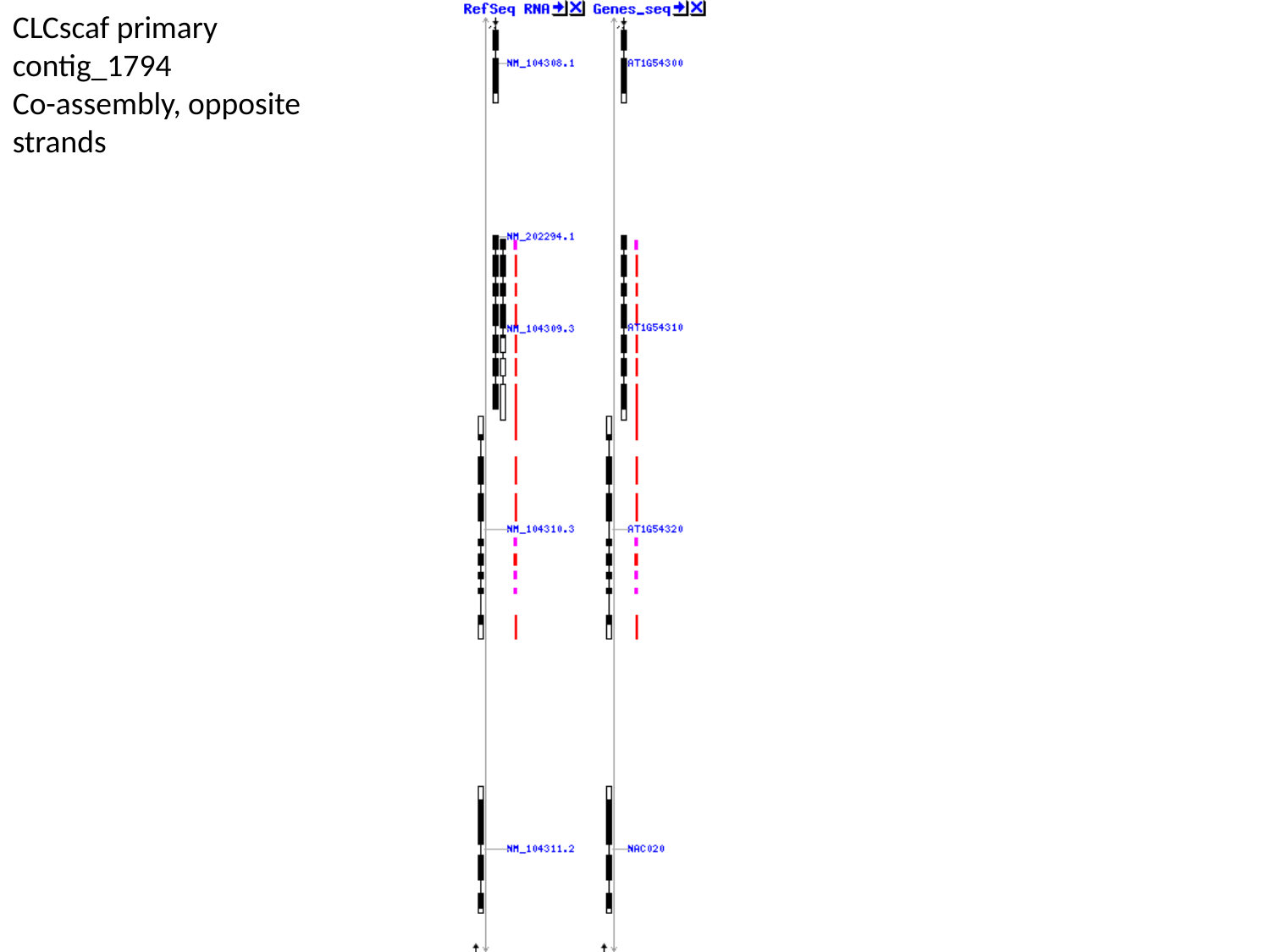

CLCscaf primary
contig_1794
Co-assembly, opposite strands

## Slide 10
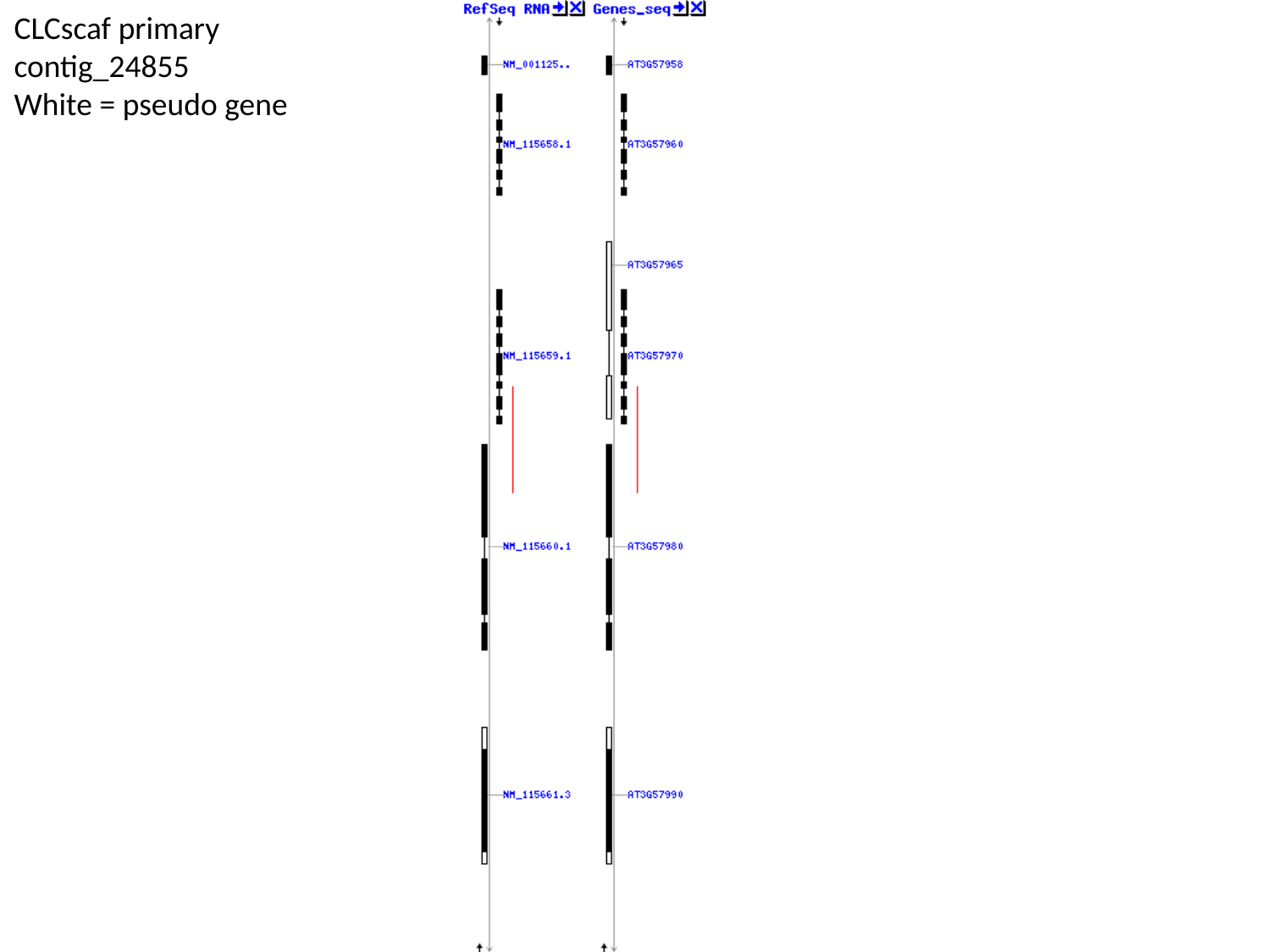

CLCscaf primary
contig_24855
White = pseudo gene

## Slide 11
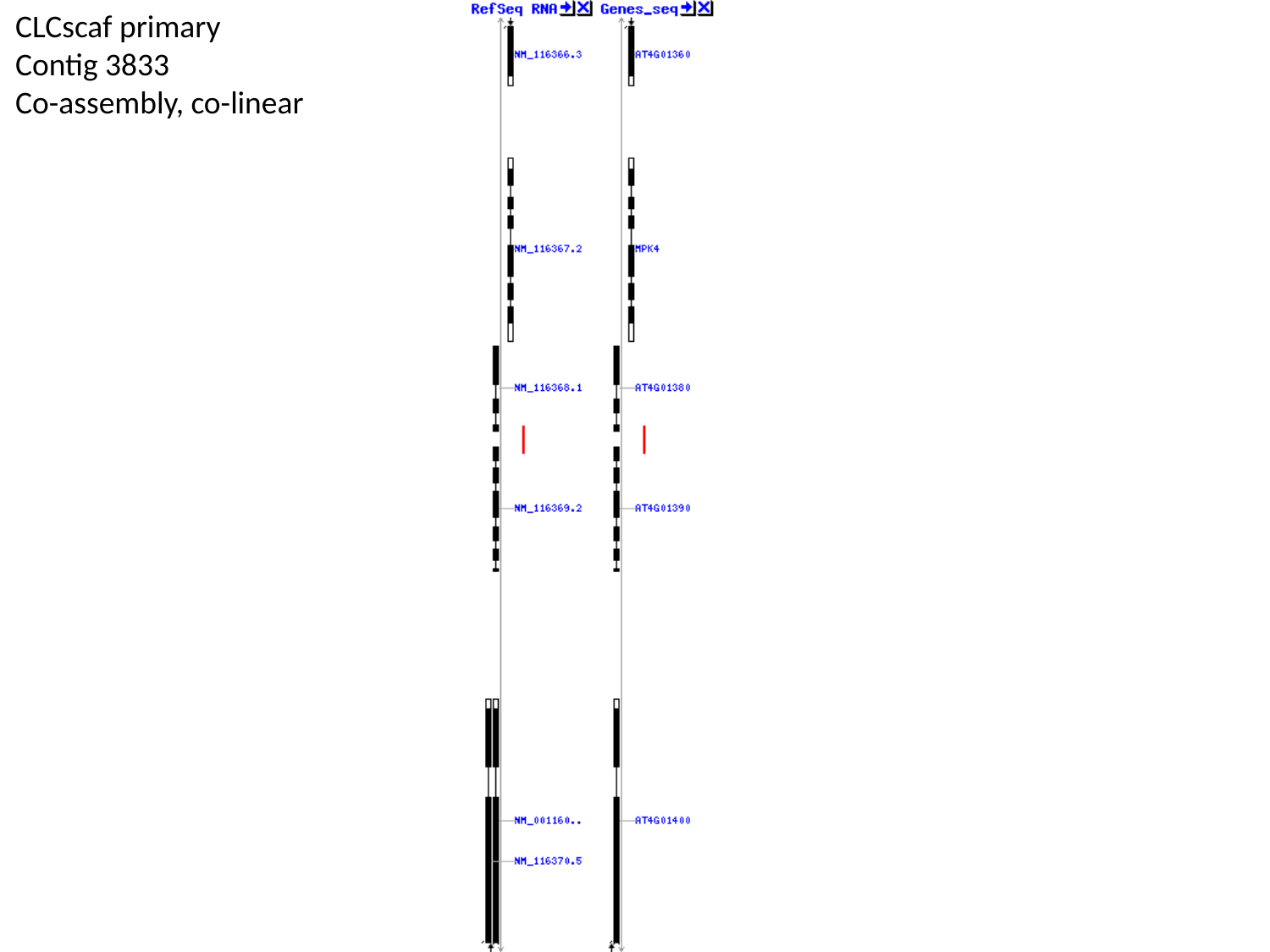

CLCscaf primary
Contig 3833
Co-assembly, co-linear

## Slide 12
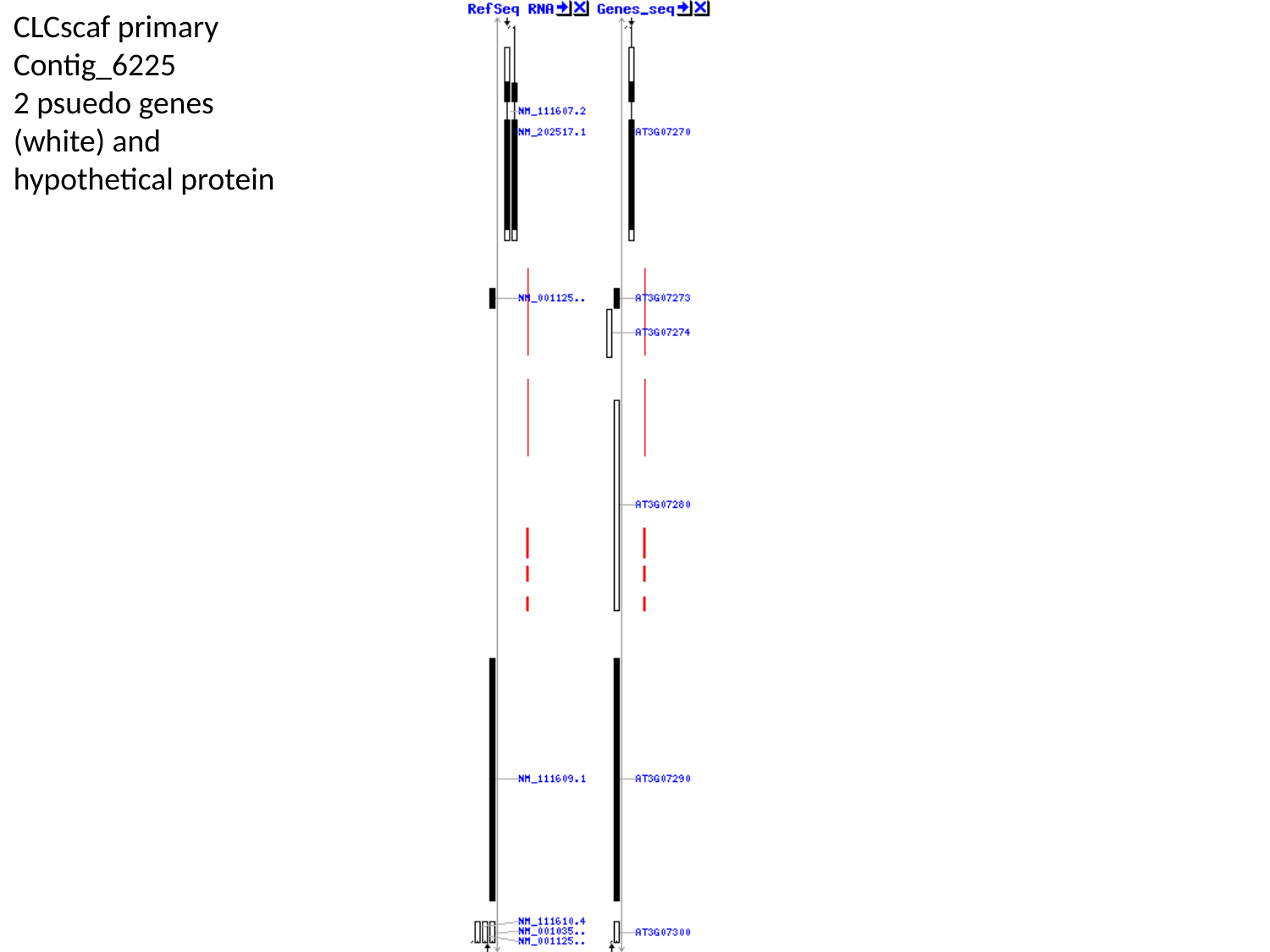

CLCscaf primary
Contig_6225
2 psuedo genes (white) and hypothetical protein

## Slide 13
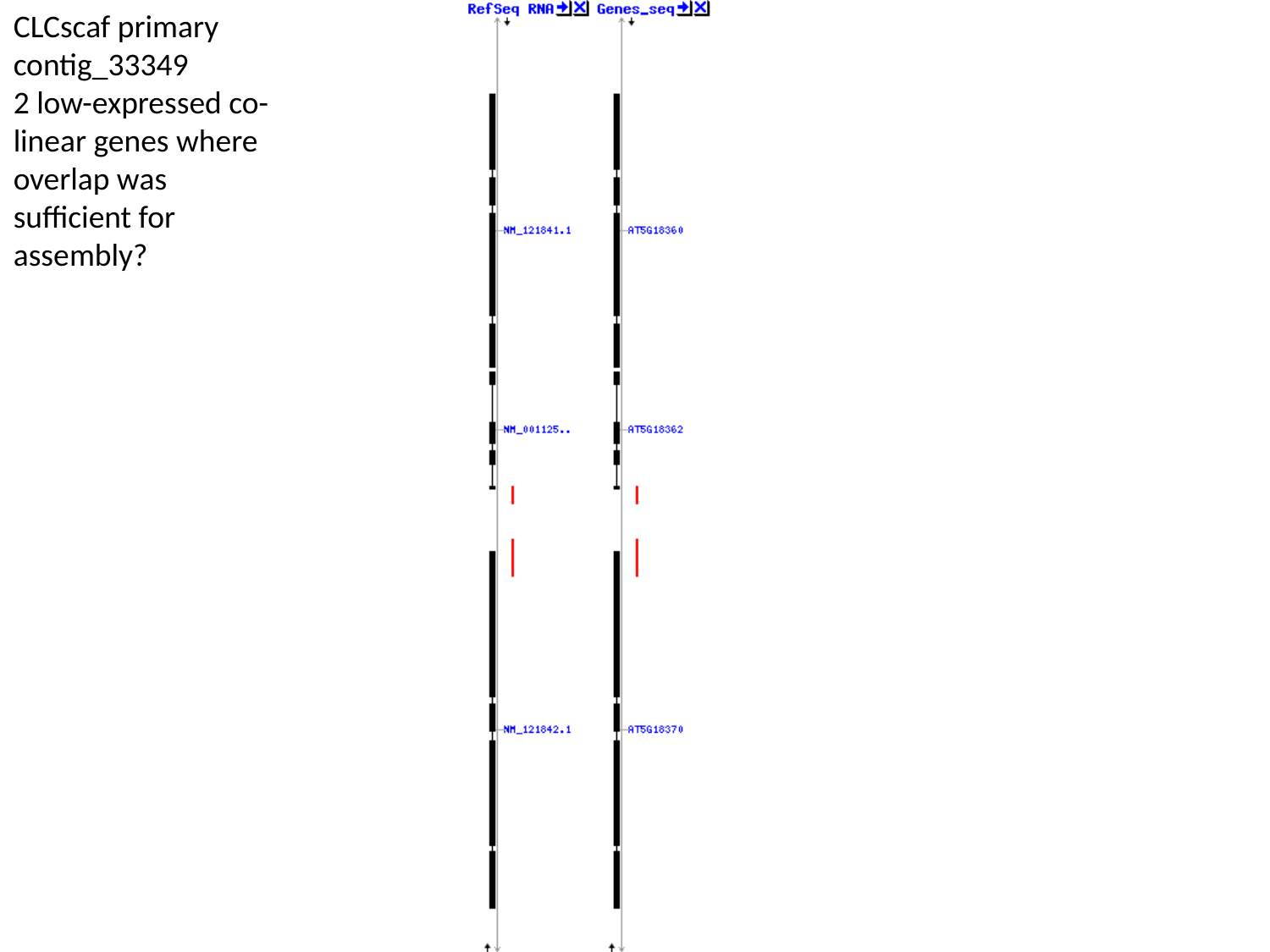

CLCscaf primary
contig_33349
2 low-expressed co-linear genes where overlap was sufficient for assembly?

## Slide 14
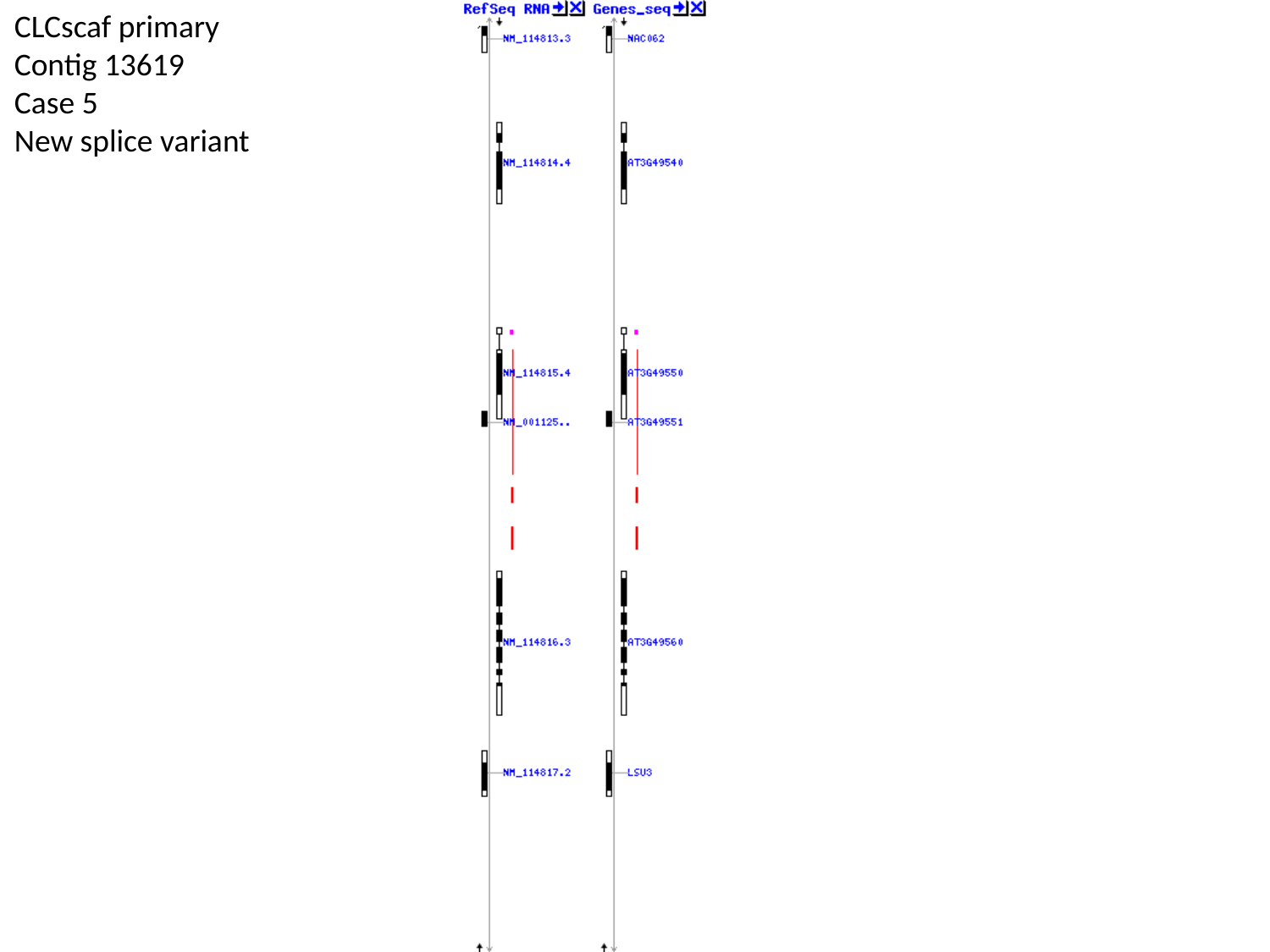

CLCscaf primary
Contig 13619
Case 5
New splice variant

## Slide 15
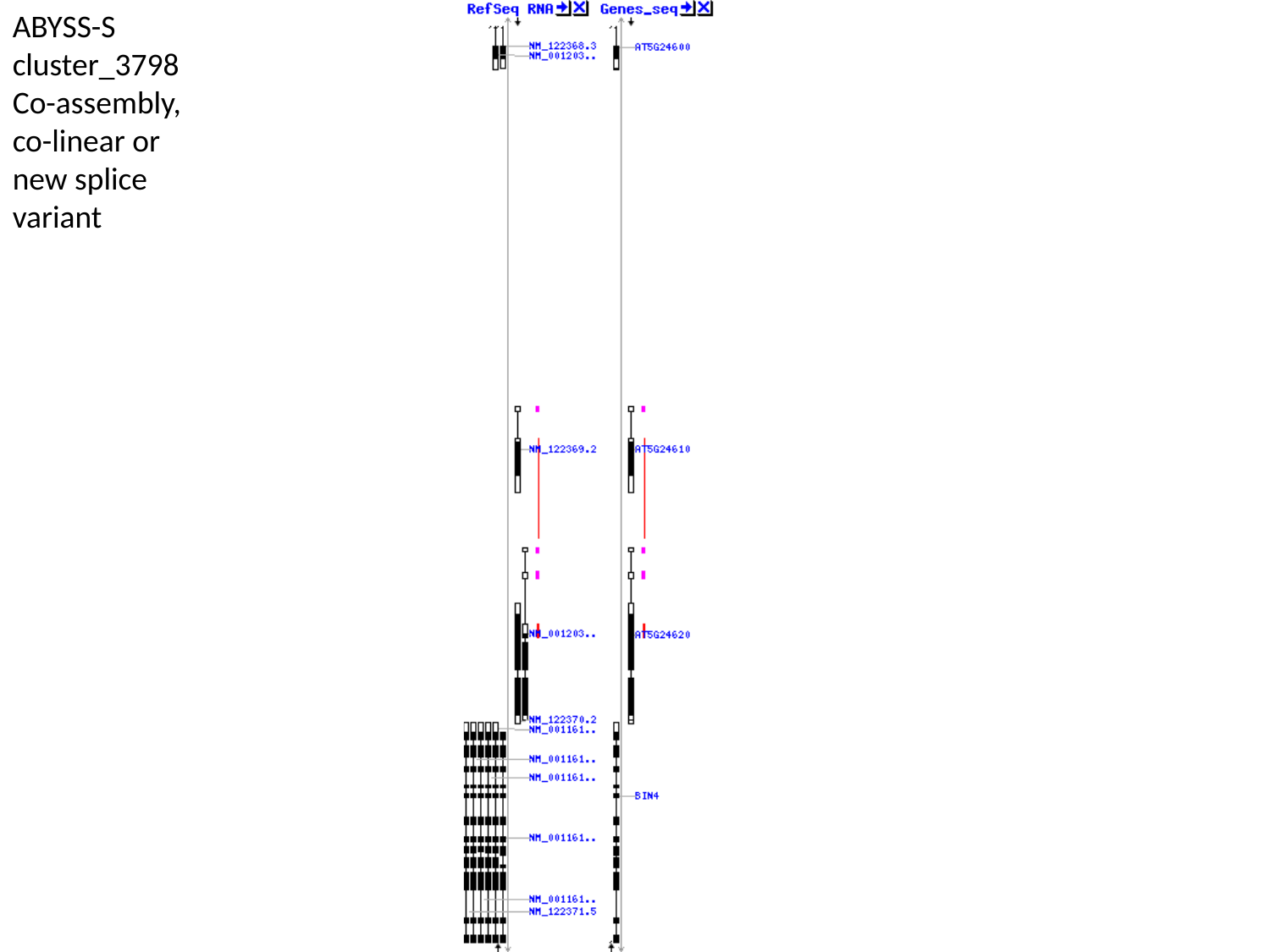

ABYSS-S cluster_3798
Co-assembly, co-linear or new splice variant

## Slide 16
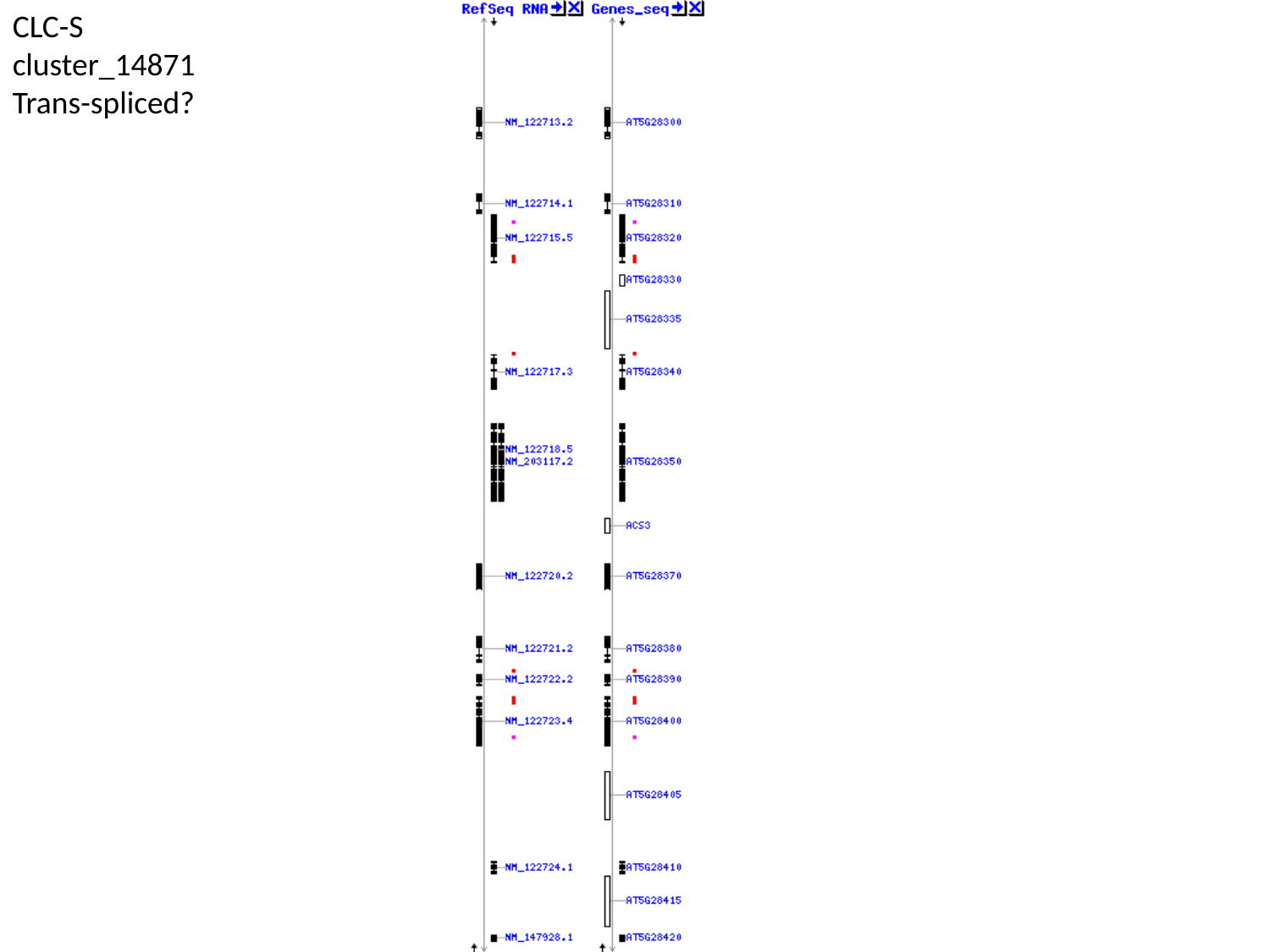

CLC-S cluster_14871
Trans-spliced?
