# Supplementary material for: Selecting Superior De Novo Transcriptome Assemblies: Lessons Learned by Leveraging the Best Plant Genome
Source: PLoS One. 2016 Jan 5;11(1):e0146062. doi: 10.1371/journal.pone.0146062 (PMC4701411; doi:10.1371/journal.pone.0146062)

## Slide 1
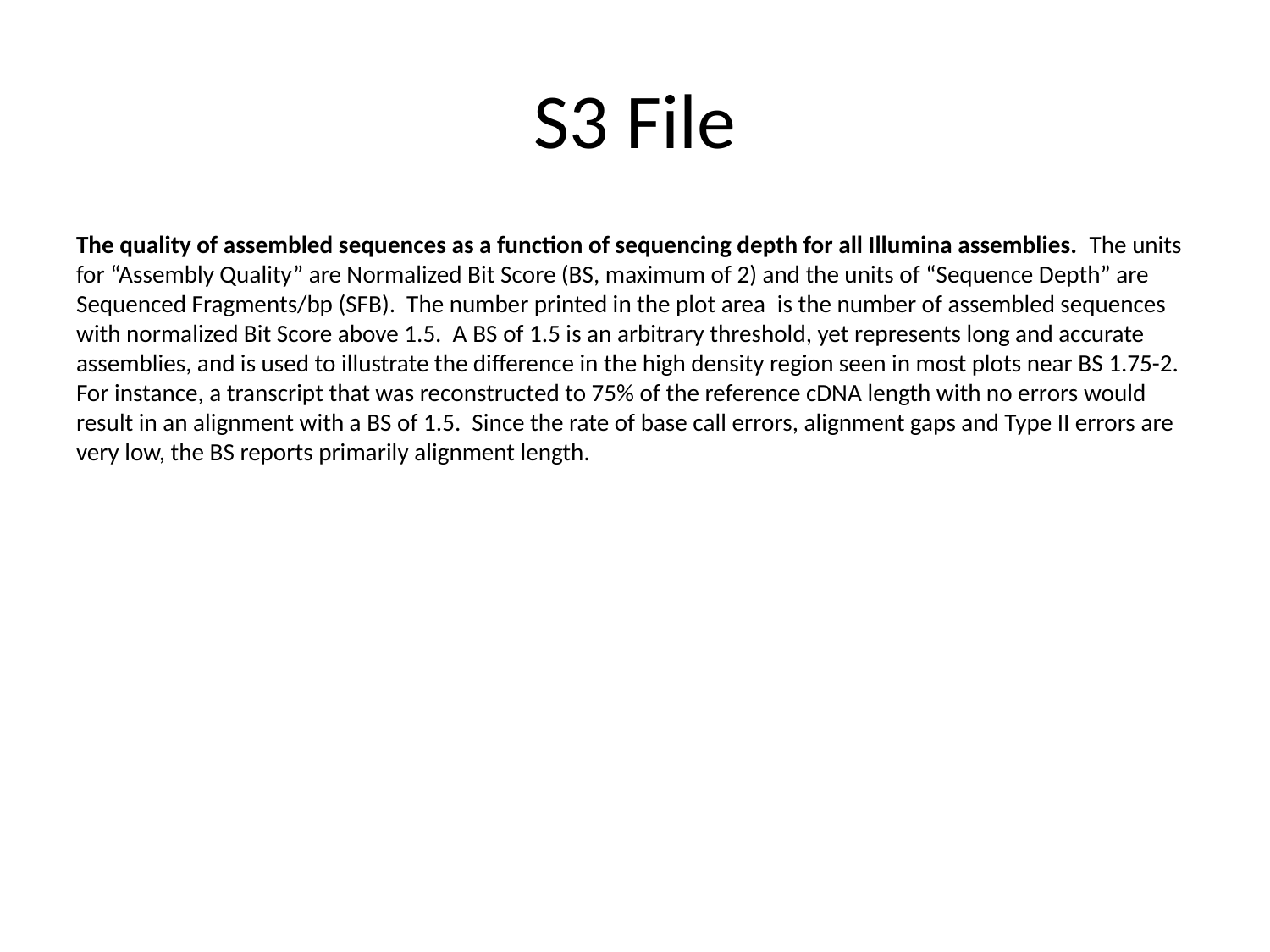

# S3 File
The quality of assembled sequences as a function of sequencing depth for all Illumina assemblies. The units for “Assembly Quality” are Normalized Bit Score (BS, maximum of 2) and the units of “Sequence Depth” are Sequenced Fragments/bp (SFB). The number printed in the plot area  is the number of assembled sequences with normalized Bit Score above 1.5. A BS of 1.5 is an arbitrary threshold, yet represents long and accurate assemblies, and is used to illustrate the difference in the high density region seen in most plots near BS 1.75-2. For instance, a transcript that was reconstructed to 75% of the reference cDNA length with no errors would result in an alignment with a BS of 1.5. Since the rate of base call errors, alignment gaps and Type II errors are very low, the BS reports primarily alignment length.

## Slide 2
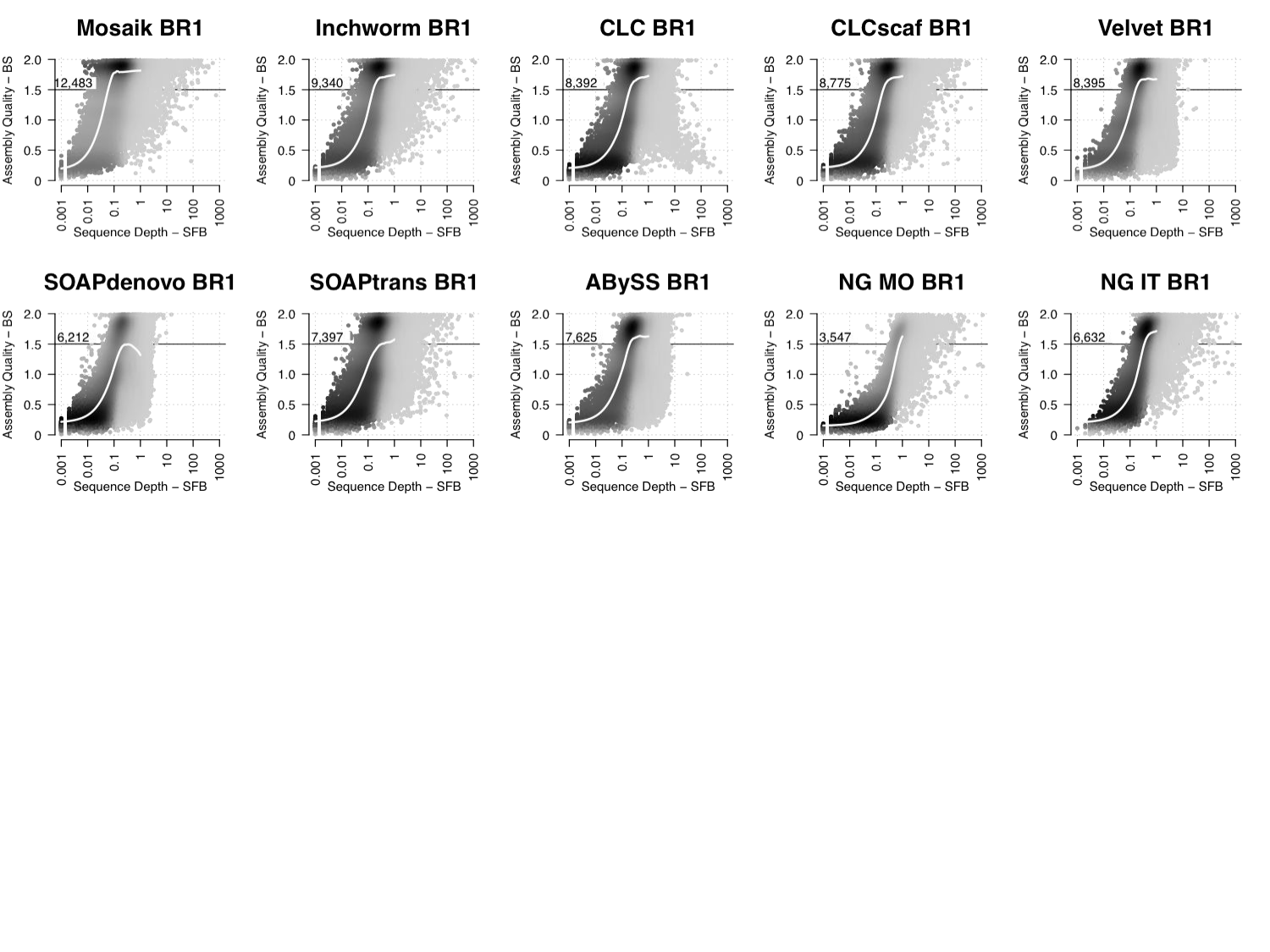

## Slide 3
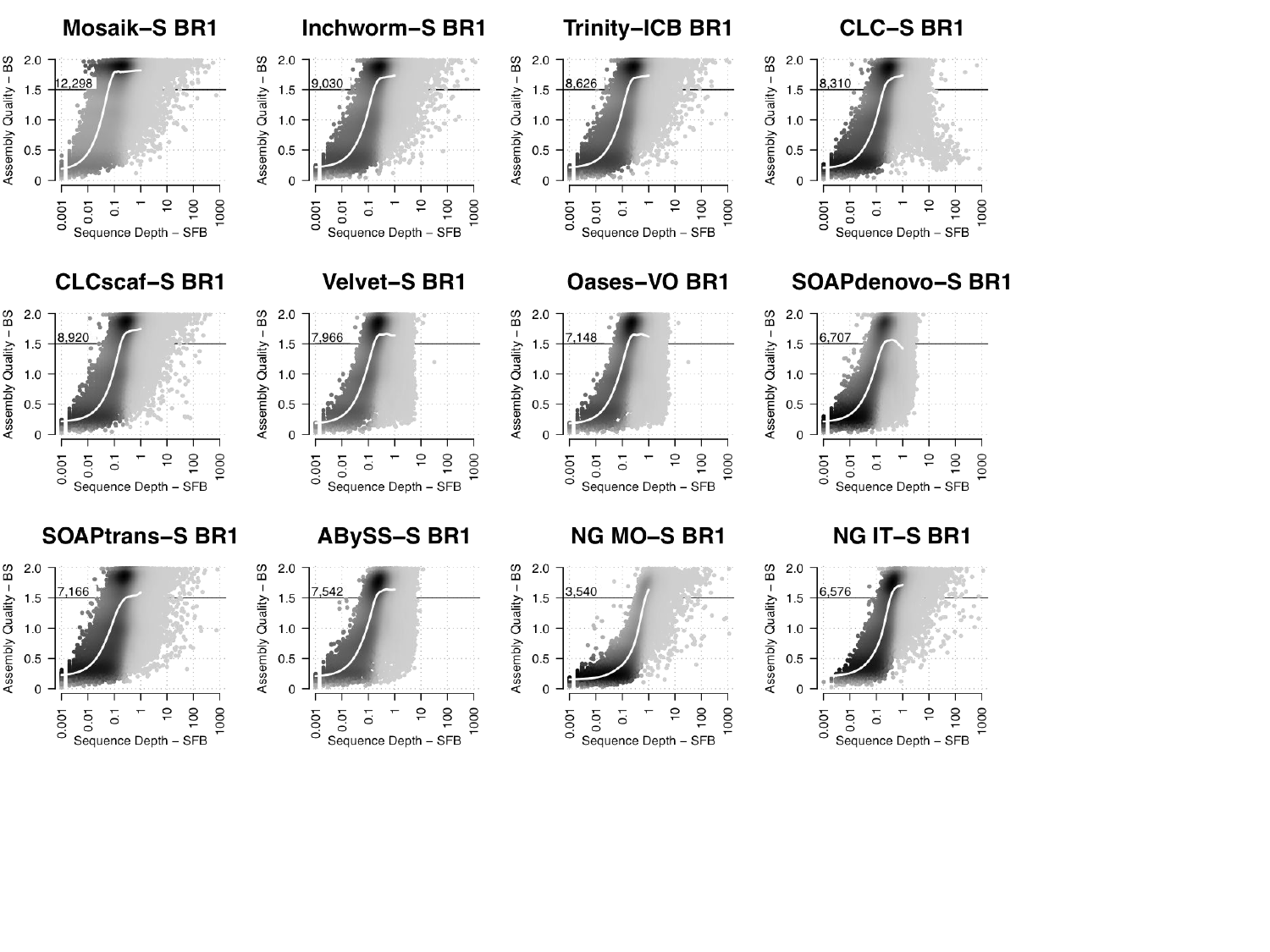

## Slide 4
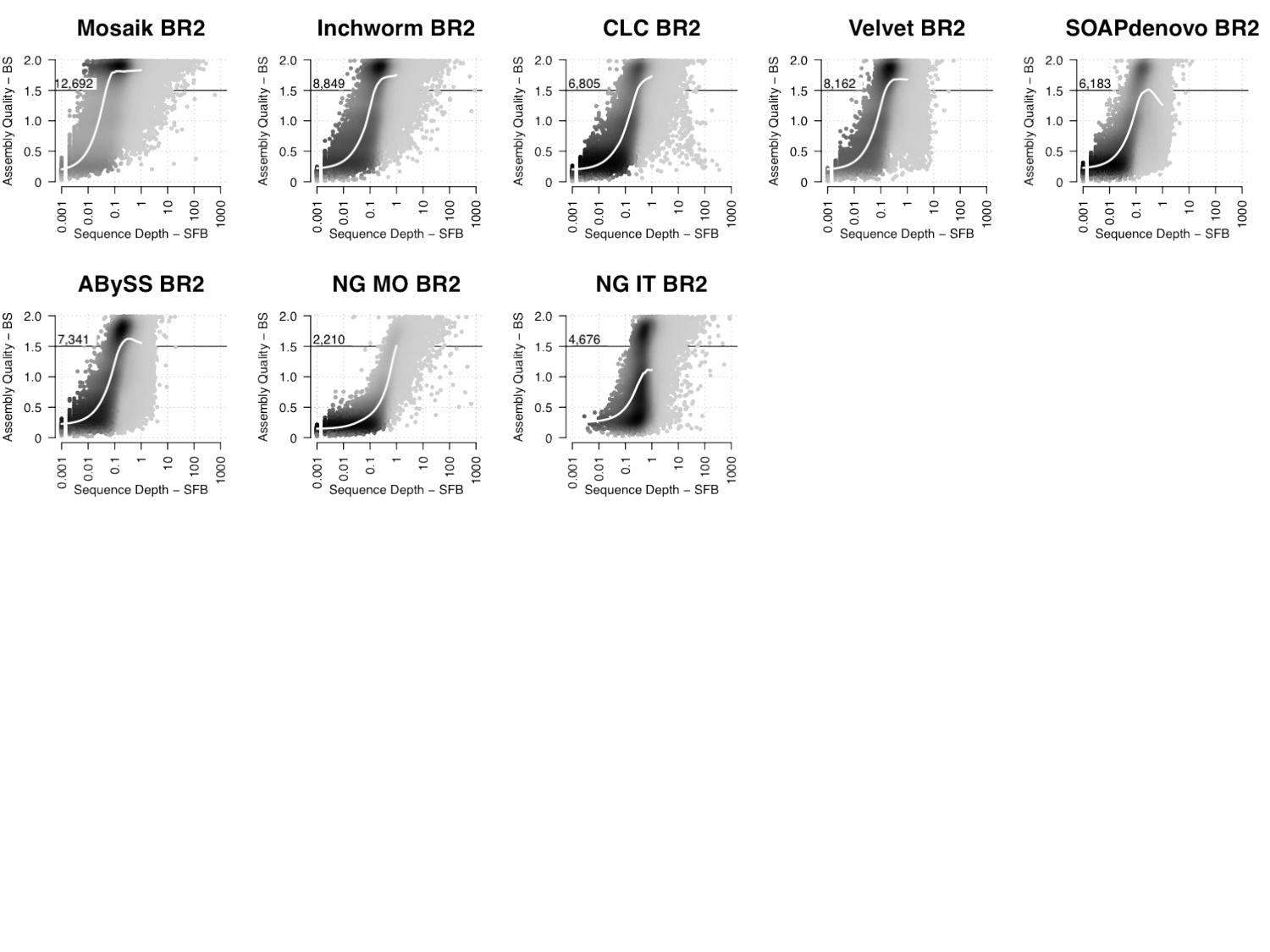

## Slide 5
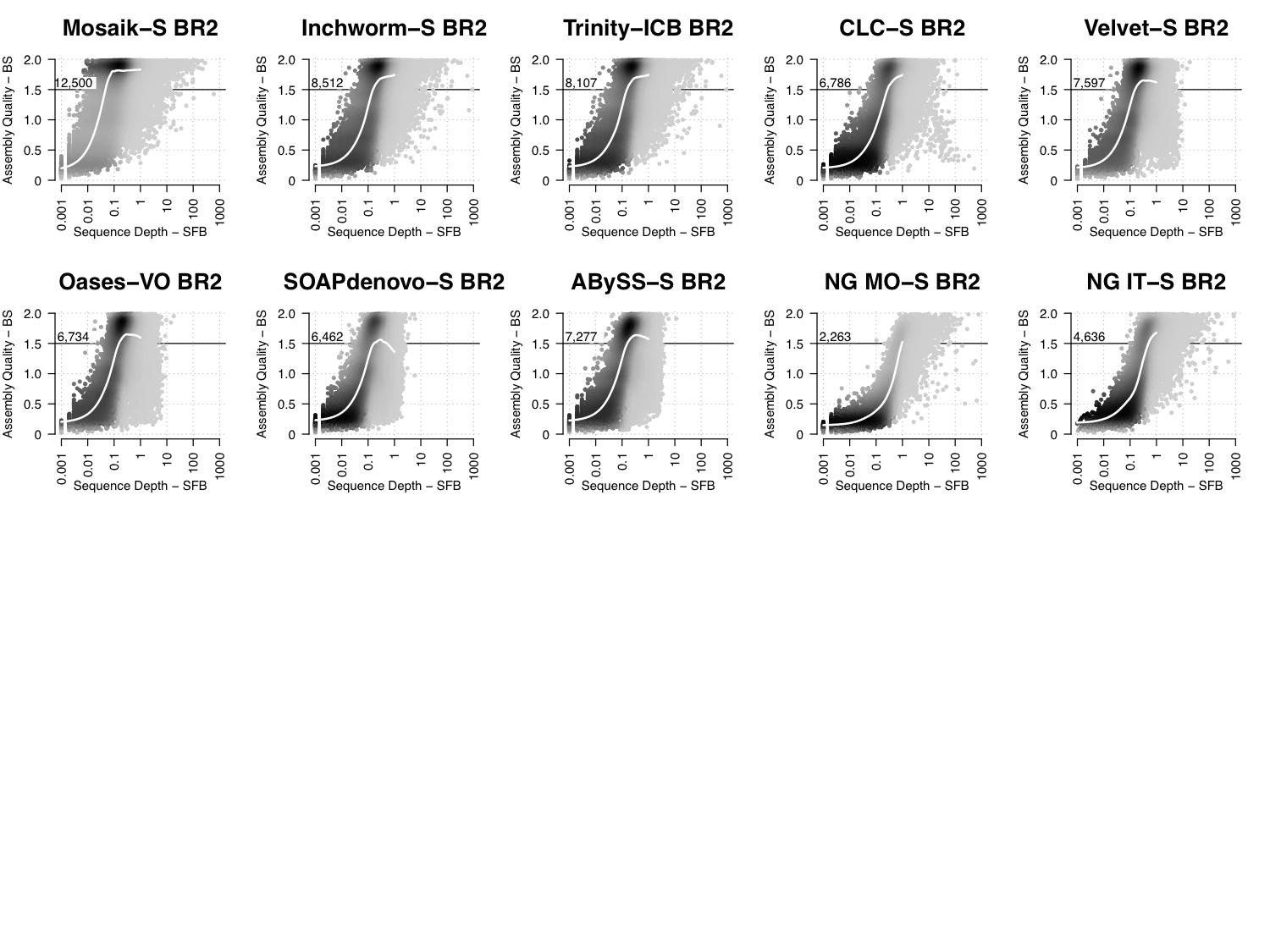

## Slide 6
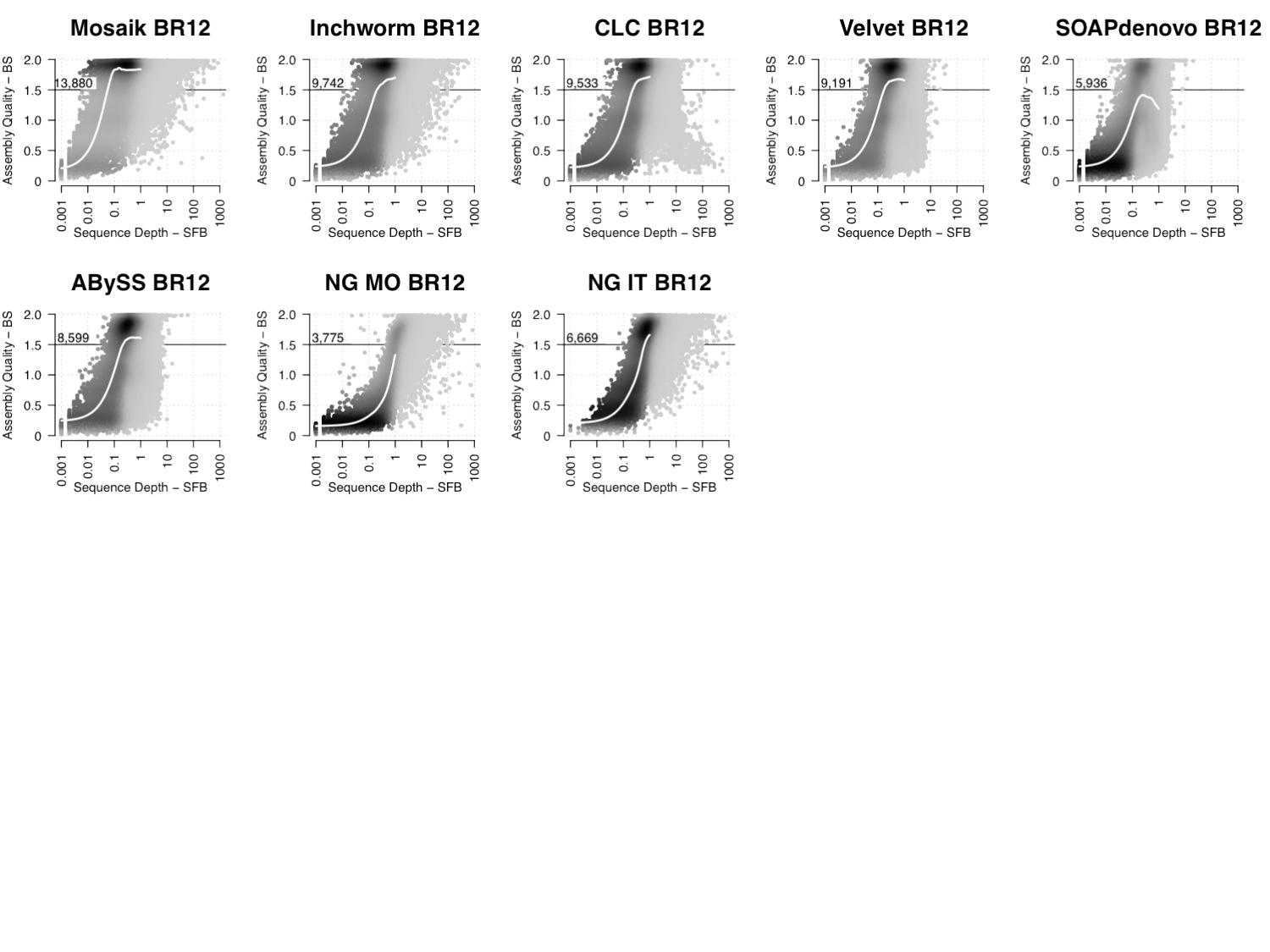

## Slide 7
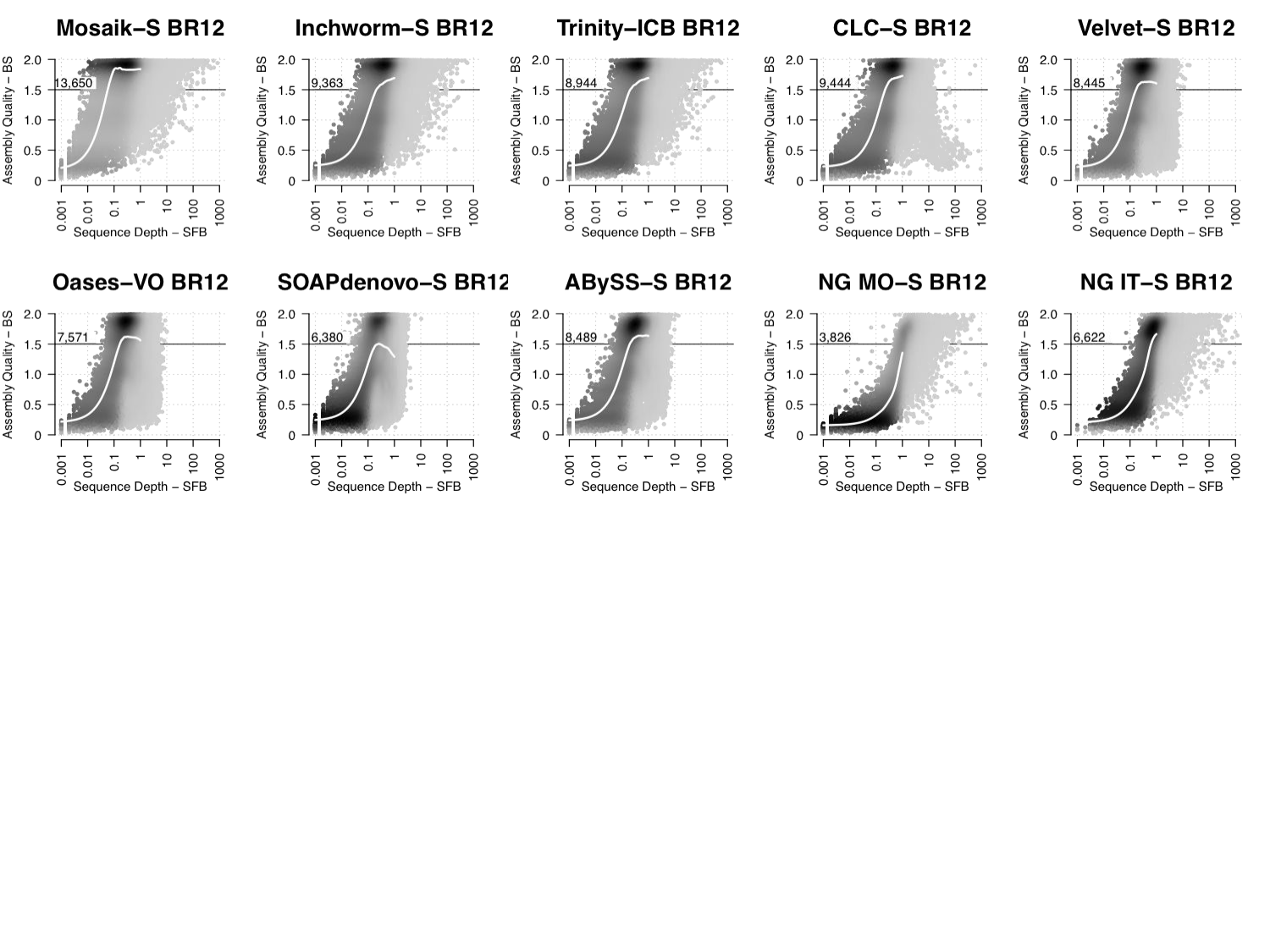

## Slide 8
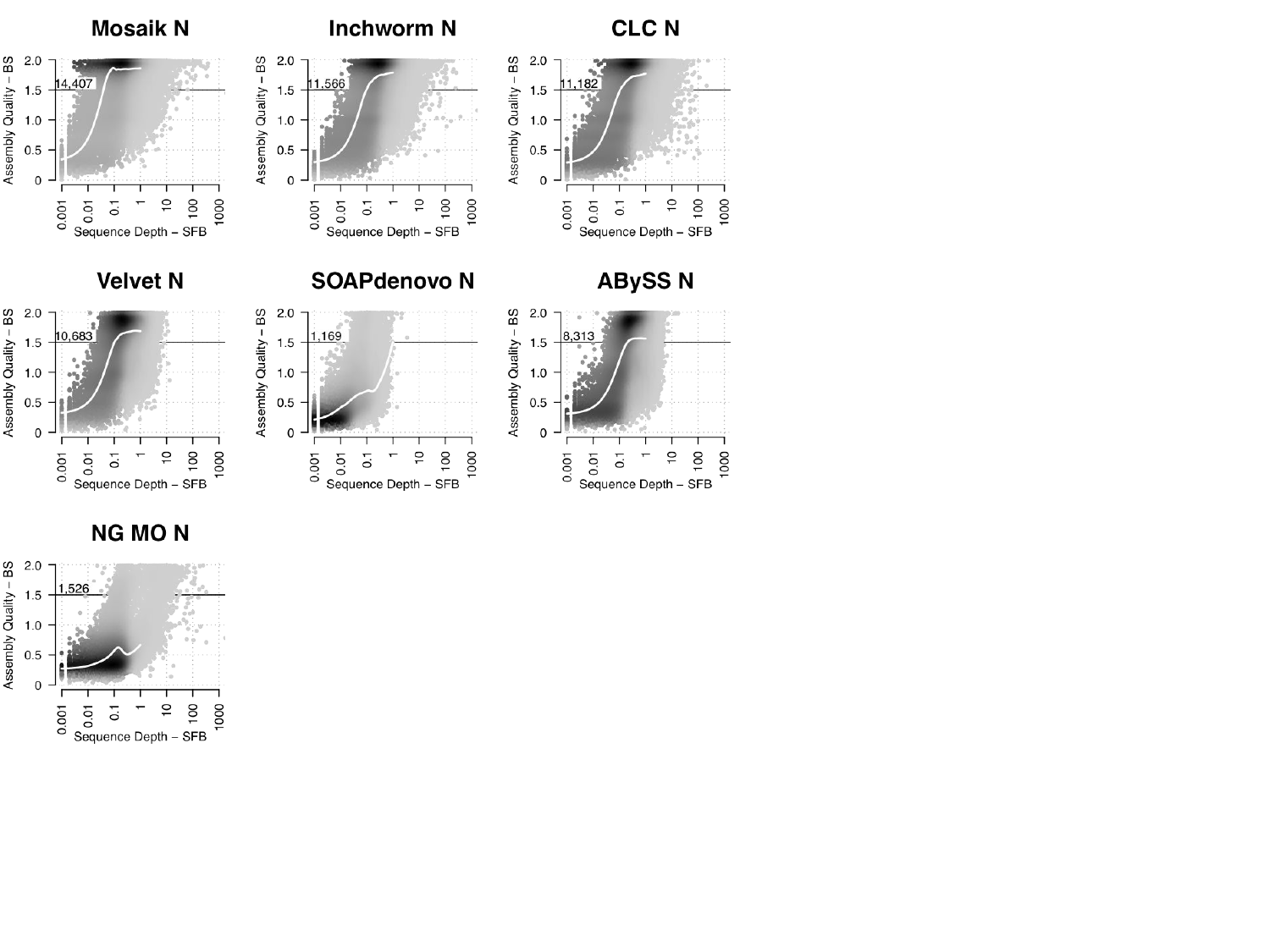

## Slide 9
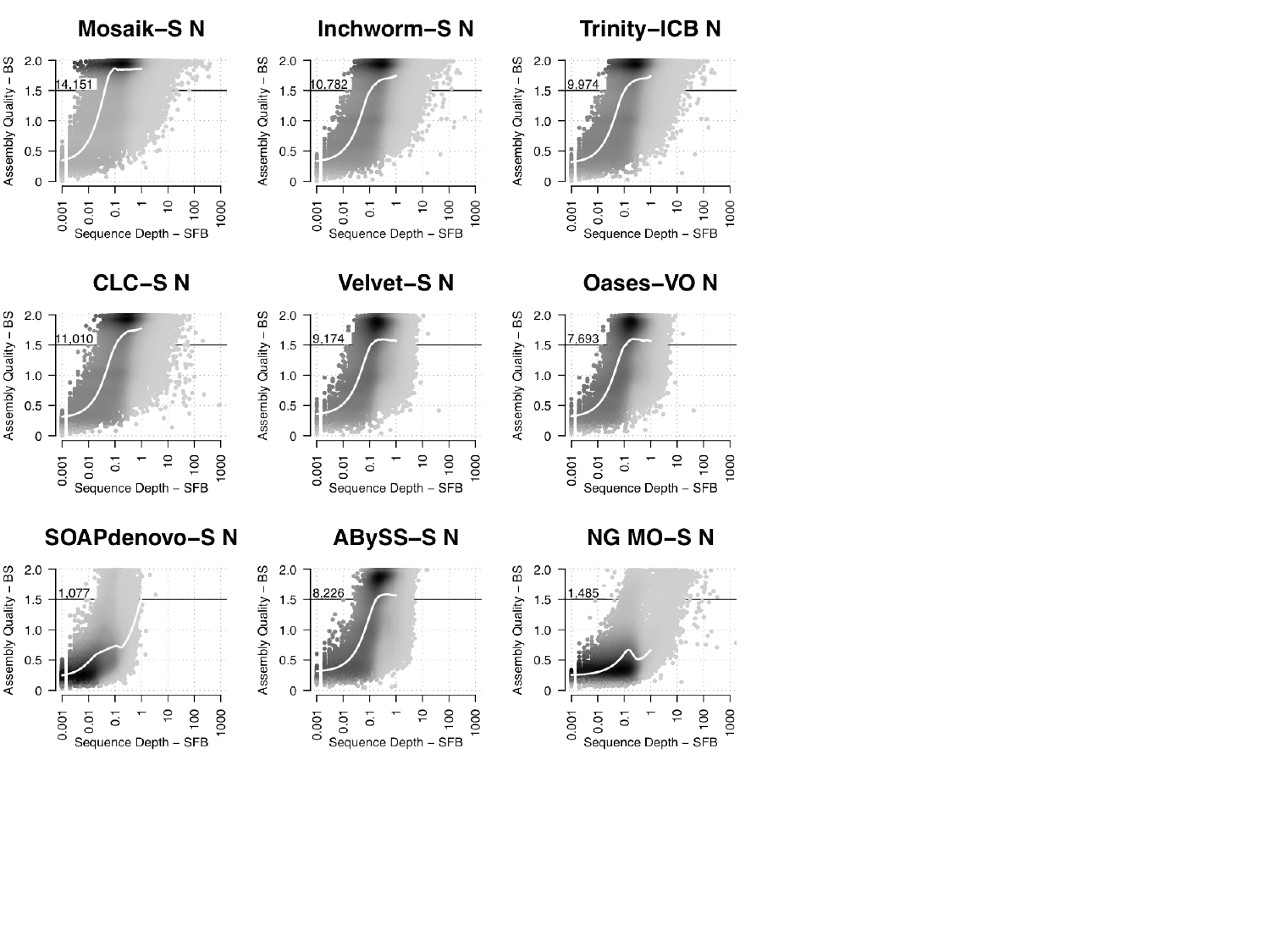

Supplement: S3 File — The units for “Assembly Quality” are Normalized Bit Score (BS, maximum of 2) and the units of “Sequence Depth” are Sequenced Fragments/bp (SFB). The number printed in the plot area is the number of assembled sequences with normalized Bit Score above 1.5. A BS of 1.5 is an arbitrary threshold, yet represents long and accurate assemblies, and is used to illustrate the difference in the high density region seen in most plots near BS 1.75–2. For instance, a transcript that was reconstructed to 75% of the reference cDNA length with no errors would result in an alignment with a BS of 1.5. Since the rate of base call errors, alignment gaps and Type II errors are low, the BS reports primarily alignment length. (PPTX) [file pone.0146062.s018.pptx]
