# Supplementary material for: Selecting Superior De Novo Transcriptome Assemblies: Lessons Learned by Leveraging the Best Plant Genome
Source: PLoS One. 2016 Jan 5;11(1):e0146062. doi: 10.1371/journal.pone.0146062 (PMC4701411; doi:10.1371/journal.pone.0146062)
Supplement: S4 File — This file details additional efforts to understand the reasons for disagreement between the two analyses. See also S10, S11 and S12 Figs. (DOCX) [file pone.0146062.s019.docx]

**The poorly correlated candidates summary – (See S10 Fig, S11 Fig, and S12 Fig)**

**Strong signal in Array, no signal in RNA-Seq (reads mapped to probes)**

**AT1G24851.1** – No reads mapped to the probes for this gene. qPCR failed to detect transcripts in either biological replicate. Low levels of expression were detected when reads were mapped to the complete collection of TAIR10 cDNAs, suggesting a false positive on the array. The moderate SFB (0.3) suggests that the transcript has sufficient reads for assembly and its absence from the *de novo* assemblies of biological replicates 1 and 2 is surprising and may indicate a innaccurate annotation in TAIR10. The SFB is higher in combined replicate and normalized assemblies, though is still absent from the assembly. This suggests that the locus annotation is inaccurate and its transcriptional product is poorly understood, therefore making an alignment-based annotation challenging.

**AT4G33120.1** - No reads mapped to the array probes for this gene. qPCR failed to detect transcripts in either biological replicate. The average fpkm (0.032) and SFB (1.32) suggest there are sufficient reads for assembly, though no assembler considered in this analysis produced a unigene that can be assigned to the locus. This suggests that the locus annotation is inaccurate and/or its transcriptional product is poorly understood, therefore making a reference based annotation challenging.

**AT5G43640.1** - No reads mapped to the array probes for this gene. qPCR failed to detect transcripts in either biological replicate. The average SFB for this gene is 4.75. Transcripts with SFB >1 are not necessarily assembled well, or even at all. Such is the case for this transcript, excessive reads and no RNA-Seq signal at the probe suggest that the locus annotation is inaccurate and its transcriptional product is poorly understood, therefore making an alignment-based annotation challenging.

**Strong signal in RNA-Seq (reads mapped to probes), proportionally weak signal in Array**

**AT1G24996.1** - A transcript representing this gene was present in the BR1 assembly by Oases and in assemblies by Oases and ABySS in the combined replicate assemblies. Roughly 4500 reads from each biological replicate mapped to the TAIR10 cDNA covering the entire reference transcript. This transcript had sufficient reads to put it well above the threshold of assembly for many of the assemblers. We used the qRT-PCR primers to amplify an approximately 100bp region from each of the 12 candidates using DNase treated RNA in a properly controlled PCR experiment. We annealed the primers at 60^o^C, allowed a 1 minute extension time and completed 40 cycles to verify a clean qRT-PCR product. Those primer pairs that yielded reliable qRT-PCR amplification for the 6 well correlated candidates, and for AT3G24480.1, yielded a clean product of expected size. Those primer pairs that yielded unreliable or undetectable results in the qRT-PCR assay yielded similarly weak or undectable signal save one: AT1G24996.1. The product of the PCR reaction using the qRT-PCR primers yielded a ~550bp product, not the expected 100bp product. The qRT-PCR primers were aligned to the TAIR10 cDNA for AT1G24996.1 revealing that they flanked the annotated intron and would yield a 551bp product if a partially processed transcript was the template (RT negative cDNA was used as a control for genomic DNA contamination and gave no signal). This result suggests that the predominant from of this transcript in young leaf tissue is immature, differentially spliced or partially processed.

**AT3G24480.1** - A single unigene from a primary CLC assembly that mapped to AT3G24480.1 revealed a novel ~800bp low complexity region and a single bp deletion in the array probe binding region, suggesting that the poor Microarray vs RNA-Seq correlation may be explained by an inaccurate gene model. We verified the presence of this novel 800bp region in other *de novo* assemblies considered in this study. We attempted to amplify this gene from cDNA and genomic DNA and were unsuccessful, though we did amplify a closely related gene. All supporting EST data (http://gbrowse.arabidopsis.org/) consisted of ESTs that mapped to the 3’ end of the TAIR10 cDNA. A single clone (GenBank: AI995373.1) from inflorescence tissue extended further 5’ than any other EST and was gapped in its alignment with the contig *and* the TAIR10 cDNA. Further, proteomics data retrieved from AtProteome (http://fgcz-atproteome.unizh.ch) revealed that the poly-peptide was only detected in cotyledons, buds, flowers and siliques and not in juvenile leaves. An intact reading frame was identified by considering the annotated start codon, novel indel, and gapped *de novo* EST. Together, this evidence suggests that we have detected an immature transcript in young leaf that is a product of a poorly annotated locus in the Arabidopsis genome. This transcript is represented in all assemblies save the SOAPdenovo assembly of the combined biological replicates.

**AT4G02970.1** - qPCR failed to detect transcripts in either biological replicate. A CLC unigene that represented AT4G02970.1 was identified and contained a region that aligned perfectly with an annotated intron suggesting a partially processed transcript was assembled *de novo.* A search of AtProteome (http://fgcz-atproteome.unizh.ch) revealed that the peptide has only been detected in dark grown cell cultures yet the available ESTs (http://gbrowse.arabidopsis.org/) support the annotated intron as part of mature transcript (GenBank: AV549571.1 & AA597599.1). This transcript is represented by the majority of *de novo* assemblies considered in this study.
